# Optimized immunosuppression to prevent graft failure in renal transplant recipients with HLA antibodies (OuTSMART): a randomised controlled trial

**DOI:** 10.1016/j.eclinm.2022.101819

**Published:** 2023-01-12

**Authors:** Dominic Stringer, Leanne Gardner, Olivia Shaw, Brendan Clarke, David Briggs, Judith Worthington, Matthew Buckland, Guilherme Danzi, Rachel Hilton, Michael Picton, Raj Thuraisingham, Richard Borrows, Richard Baker, Keith McCullough, John Stoves, Mysore Phanish, Sapna Shah, Kin Yee Shiu, Stephen B. Walsh, Aimun Ahmed, Waqar Ayub, Janet Hegarty, Rose Tinch-Taylor, Evangelos Georgiou, Natalie Bidad, Ayşenur Kılıç, Zoe Moon, Robert Horne, Paul McCrone, Joanna Kelly, Caroline Murphy, Janet Peacock, Anthony Dorling

**Affiliations:** a Biostatistics and Health Informatics, The Institute of Psychiatry, Psychology & Neuroscience, King's College London, London, UK; b King's Clinical Trials Unit, King's College London, London, UK; c Centre for Nephrology, Urology and Transplantation, Department of Inflammation Biology, King's College London, Guy's Hospital, Great Maze Pond, London, SE1 9RT, UK; d Clinical Transplantation Laboratory, Viapath Analytics LLP, London, UK; e Transplant Immunology, Level 09 Gledhow Wing, St James's University Hospital, Beckett Street, Leeds, LS9 7TF, UK; f NHSBT Birmingham, Vincent Drive, Edgbaston, Birmingham, B15 2SG, UK; g Transplantation Laboratory, Manchester Royal Infirmary, Oxford Road, Manchester, M13 9WL, UK; h Clinical Transplantation Laboratory, The Royal London Hospital, 2nd Floor, Pathology and Pharmacy Building, 80 Newark Street, London, E1 1BB, UK; i Renal Unit, Hospital das Clínicas da Universidade Federal de Pernambuco, Av. Prof. Moraes Rego, 1235 - Cidade Universitária, Recife - PE, 50670-901, Brazil; j Department of Nephrology and Transplantation, Guy's Hospital, Great Maze Pond, London, SE1 9RT, UK; k Department of Renal Medicine, Manchester Royal Infirmary, Oxford Road, Manchester, M13 9WL, UK; l Department of Renal Medicine and Transplantation, Barts Health NHS Trust, London, E1 1BB, UK; m Renal Unit, University Hospital Birmingham, Edgbaston, Birmingham, B15 2LN, UK; n Renal Unit, St James's University Hospital, Beckett Street, Leeds, LS9 7TF, UK; o Renal Unit, York Teaching Hospital NHS Foundation Trust, York, YO31 8HE, UK; p Renal Unit, Bradford Teaching Hospitals NHS Foundation Trust, Bradford, BD5 0NA, UK; q Renal Unit, Epsom and St Helier University Hospitals NHS Trust, Surrey, UK; r Renal Unit, King's College Hospital, London, SE5 9RJ, UK; s UCL Department of Renal Medicine, Royal Free London NHS Foundation Trust, London, NW3 2QG, UK; t Renal Unit, Lancashire Teaching Hospitals NHS Foundation Trust, Preston, PR2 9HT, UK; u Renal Unit, University Hospitals Coventry and Warwickshire NHS Trust, Coventry, CV2 2DX, UK; v Renal Unit, Salford Royal NHS Foundation Trust, Salford, M6 8HD, UK; w Centre for Behavioural Medicine, UCL School of Pharmacy, University College London, London, WC1H 9JP, UK; x Faculty of Education, Health and Human Sciences, University of Greenwich, London, UK; y School of Life Course and Population Sciences, King's College London, London, UK; z Department of Epidemiology, Geisel School of Medicine at Dartmouth, Dartmouth College, USA

**Keywords:** Kidney transplantation, HLA antibodies, Optimised immunosuppression, Stratified medicine, Kidney allograft failure

## Abstract

**Background:**

3% of kidney transplant recipients return to dialysis annually upon allograft failure. Development of antibodies (Ab) against human leukocyte antigens (HLA) is a validated prognostic biomarker of allograft failure. We tested whether screening for HLA Ab, combined with an intervention to improve adherence and optimization of immunosuppression could prevent allograft failure.

**Methods:**

Prospective, open-labelled randomised biomarker-based strategy (hybrid) trial in 13 UK transplant centres [EudraCT (2012-004308-36) and ISRCTN (46157828)]. Patients were randomly allocated (1:1) to unblinded or double-blinded arms and screened every 8 months. Unblinded HLA Ab+ patients were interviewed to encourage medication adherence and had tailored optimisation of Tacrolimus, Mycophenolate mofetil and Prednisolone. The primary outcome was time to graft failure in an intention to treat analysis. The trial had 80% power to detect a hazard ratio of 0.49 in donor specific antibody (DSA)+ patients.

**Findings:**

From 11/9/13 to 27/10/16, 5519 were screened for eligibility and 2037 randomised (1028 to unblinded care and 1009 to double blinded care). We identified 198 with DSA and 818 with non-DSA. Development of DSA, but not non-DSA was predictive of graft failure. HRs for graft failure in unblinded DSA+ and non-DSA+ groups were 1.54 (95% CI: 0.72 to 3.30) and 0.97 (0.54–1.74) respectively, providing no evidence of an intervention effect. Non-inferiority for the overall unblinded versus blinded comparison was not demonstrated as the upper confidence limit of the HR for graft failure exceeded 1.4 (1.02, 95% CI: 0.72 to 1.44). The only secondary endpoint reduced in the unblinded arm was biopsy-proven rejection.

**Interpretation:**

Intervention to improve adherence and optimize immunosuppression does not delay failure of renal transplants after development of DSA. Whilst DSA predicts increased risk of allograft failure, novel interventions are needed before screening can be used to direct therapy.

**Funding:**

The National Institute for Health Research Efficacy and Mechanism Evaluation programme grant (ref 11/100/34).

**Research in context**

**Evidence before this study**

In kidney transplant recipients, chronic immune-mediated injury, presenting as progressive graft dysfunction leading to graft failure, significantly limits the long-term survival of kidney transplants, resulting in tens of thousands of patients worldwide returning to dialysis each year. Prior to OuTSMART, the prognostic link between developing antibodies (Abs) to human leukocyte antigens (HLA) after transplantation and subsequent allograft failure had been established by small retrospective case control studies and later by several prospective cohort studies, particularly if the HLA Abs were donor specific Abs (DSA). A couple of recent systematic reviews, published after OuTSMART began, have confirmed this link. Prior to this trial only single centre observational or retrospective interventional studies had reported using development of DSA to guide treatment, some suggesting that increasing oral immunosuppression had a beneficial impact on graft survival.

**Added value of this study**

OuTSMART is the first RCT to test, in the context of a screening programme for development of HLA Ab, a structured intervention consisting of interview to convey the importance of medication adherence, followed by a patient-tailored optimisation of oral immunosuppression to a combination of tacrolimus, mycophenolate mofetil and prednisolone. Our data confirm that development of DSA, but not other HLA Ab, is associated with increased risk of allograft failure. However, despite evidence of increased adherence, a significant increase in the levels of immunosuppression taken by the treated population, and a reduction in biopsy-proven rejection, these interventions had no impact on allograft survival, nor on any of our other secondary endpoints.

**Implications of all the available evidence**

Screening for DSA does identify a population at increased risk of allograft failure. Enhancing the level of oral immunosuppression taken, through increased adherence and tailored changes in immunosuppressants, does not prevent allograft failure. Until we have an effective treatment for the chronic immune injury that accompanies DSA development, widespread routine screening for DSA is difficult to justify.

## Introduction

Kidney transplantation is the gold standard treatment for end stage renal failure, but transplants do not last for the natural lifespan of most recipients. 30–40% of transplants fail within 10 years^1^ (approximately 3% annually^2^) meaning that in the USA for instance, approximately 7000 of the ∼230,000 prevalent kidney transplant patients will return to dialysis every year.^3^ Of the various reasons why this happens, the single biggest cause is immune-mediated injury, primarily directed against mismatched donor human leukocyte antigens (HLA).

Circulating antibodies (Ab) against HLA have been validated as prognostic biomarkers of kidney transplant failure by case control,^4^ and prospective observational studies,^5^ though recent systematic reviews have consistently identified low/moderate quality evidence in this area.^6^^,^^7^ HLA Ab specific for donor HLA (donor specific antibodies—DSA) carry a higher risk of graft loss compared to those that are not donor-specific (non-DSA). A prevalent hypothesis is that inappropriately low levels of immunosuppression, either physician-led or due to patient non-adherence, is an important contributor to the initiation of immune-mediated damage.^8^ Several small scale trials have tested various treatments in patients with early stage, biopsy-proven chronic rejection associated with HLA Ab, including several that indicate optimised treatment with tacrolimus and mycophenolate mofetil (MMF) can stabilise transplant function,^9^, ^10^, ^11^, ^12^, ^13^, ^14^ but to date there have been no large scale trials testing the utility of intervening prior to graft dysfunction and none that have used graft failure as the primary endpoint.

In OuTSMART, we tested the hypothesis that routine surveillance for the development of HLA Ab, combined with an intervention to improve adherence, followed by tailored optimised oral treatment in those who became HLA Ab+, would prevent the kidney allograft failure.^15^^,^^16^

## Methods

### Study design

Investigator-led, open label marker-based strategy (hybrid) randomised trial^16^^,^^17^ conducted in 13 UK transplant units (Suppl Fig. S1). Study conduct and patient safety were monitored by independent data monitoring and trial steering committees. Clinical coordination by the chief investigator (CI) was supported by the UK NIHR Clinical Research Networks. The study was approved by the MHRA and by the National Research Ethics Service Committee London (Hampstead) (12/LO/1759) and was carried out in accordance with the declaration of Helsinki (1996) and Good Clinical Practice as defined in UK clinical trial regulations. All subjects gave written informed consent. The trial is registered with EudraCT (2012-004308-36) and ISRCTN (46157828).

### Participants

All renal transplant recipients aged 18–75 years, >12 months post-transplantation, with a sufficient grasp of English and an estimated glomerular filtration rate (eGFR) > 30 mL/min/1.73 m^2^ (by 4 variable Modification of Diet in Renal Disease equation) were potentially eligible. Exclusion criteria were recipients: 1) of cross-match positive transplants requiring HLA desensitization, 2) with known HLA Ab and previous specific treatment for that Ab 3) of additional solid organ transplants 4) with a history of non-skin limited malignancy within 5 years, 5) with known positive test for hepatitis B, C or HIV, 6) with history of acute rejection requiring treatment in previous 6 months, 7) enrolled in any other studies involving Investigational Medicinal Products (IMP), 8) with known hypersensitivity to the IMPs, 9) with known hereditary disorders of carbohydrate metabolism, 10) who were pregnant or breastfeeding, 11) who, if pre-menopausal refused to consent to using suitable contraception throughout.

The prevalent transplant population in each centre (Suppl Table S1) was screened by age, time since transplant, additional organ transplant and previous known HLA Ab treatment prior to a clinic appointment. Those meeting criteria were approached for written informed consent. Post consent screening was performed to exclude anyone with a positive HepBSAg, HepBcAb, HepCAb, HIV or HCG test.

### Randomisation and blinding

After final eligibility testing, blood was sent for determination of IgG HLA Ab status (see below and Supplementary text), and the results used in the randomisation process. Allocation to blinded standard care (SC) or unblinded biomarker led care (BLC) arms was assigned (1:1) by stratified block randomisation with randomly varying block sizes of 2 or 4, using a web-based randomisation service provided by the King's Clinical Trials Unit. All recruits were randomized, including HLA Ab-negatives, and the randomisation was stratified by a) HLA Ab status, to generate 3 groups within each arm (DSA+, non-DSA+ or HLA Ab-negative), b) current immunosuppression (to ensure balanced numbers specific immunosuppressives and c) site.

The randomisation allocation was initiated by staff within the tissue typing labs to maintain physician and patient blinding to HLA Ab status within the SC groups (A1, A2, C in Suppl. Fig. S1), in whom treatment decisions were made on clinical grounds, according to local protocols. Participants in the unblinded BLC groups (B1, B2, D in Suppl. Fig.S1), and their physicians, were unblinded to their HLA Ab status and the IMPs administered open-label. There was no blinding to trial arm (participants, investigators, outcome assessors or any other staff were not blinded to whether the participant was in the SC or BLC arm).

### HLA Ab determination

The Supplementary File contains a detailed description of methodology, and the standard operating procedure for antibody testing, but briefly, serum was first tested using mixed HLA class I and class II Ab screening beads (One Lambda, Canoga Park, CA through VH Bio, Gateshead UK). Serum with a positive result on mixed bead screening was analysed using single antigen coated class I or class II beads, with a positive defined as mean fluorescence intensity (MFI) of binding ≥2000.

### Procedures (Fig. 1 & Suppl Fig. S1)

Treatment for HLA Ab+ BLC patients began with an interview to explain the importance of medication adherence. Thereafter, all were asked to change to maximum tolerated doses of MMF/mycophenolic acid (MPA) and tacrolimus, targeting 12-h post-dose levels of 4−8 μg/L. Formulations of these drugs were dictated locally. In addition, all were encouraged to take a steroid ‘boost’, consisting of prednisolone 20 mg daily for two weeks, reducing by 5 mg every two weeks down to their previous maintenance dose or 5 mg od. All changes were individually tailored and refusal to change or inability to tolerate one or more aspects of the protocol was not classed as ‘failure’. Independently of whether changes were made, all tacrolimus, MMF/MPA or prednisolone administered to HLA Ab+ BLC recruits post-interview were treated as IMPs.

Screening for HLA Ab was repeated every 8 months in HLA Ab-negative recruits, until all had had at least one repeat sample post-enrolment. No further screening was undertaken in HLA Ab+ recruits until the sample taken at their last structured visit. Minimum follow-up was therefore 32 months. Recruits changing from HLA Ab negative to positive, at any time after enrolment were asked to complete a further 32 months follow-up. To maintain blinding, the randomisation system was programmed at each screening round to choose a random group of HLA Ab-negative SC recruits to complete a further 32 months follow-up, so that the maximum time under structured follow-up for any recruit was 64 months. All recruits were asked to give a further sample for HLA Ab testing at their last visit, at which point trial-specific visits and most data collection related to secondary endpoints ceased, but data related to graft failure or death were recorded for all patients up and until the end of the trial (see below), irrespective of the length of follow-up.

### Objectives and endpoints

The initial primary objective was to compare 3-year graft failure rates in HLA Ab+ patients in the SC vs. BLC arms.^15^ Graft failure was defined as re-starting dialysis or requiring a new transplant. After 16 months recruitment, the expected 9% prevalence and 3% incidence rates of DSA were found to be 5.8% and 1.6% respectively, so the primary endpoint was changed to time to graft failure in the two groups to preserve statistical power. The primary endpoint was to be evaluated remotely at a minimum of 43 months post-randomisation.^16^ However, in 2020, as the influence of the ‘first wave’ of the COVID-19 pandemic on UK transplant recipient death rates became obvious, clinical research teams were re-deployed and routine patient visits to all hospitals were cancelled, we had to re-define the primary endpoint as that obtained at the last follow-up prior to 16th March 2020. Other changes to the protocol are described in detail in the Supplementary Appendix.

### Secondary objectives and outcomes

The secondary objectives were to determine a) time to graft failure in all patients randomised to unblinded compared to blinded HLA Ab screening: b) patient survival (all-cause mortality); c) levels of graft dysfunction (presence of proteinuria (Protein Creatinine Ratio >50) or change in eGFR; d) presence of biopsy-proven acute rejection; e) adverse effect profiles in each group, in particular presence of culture- or polymerase chain reaction (PCR)-positive infection, biopsy-proven malignancy or diabetes mellitus; f) the cost effectiveness of this screening/treatment protocol; g) the impact of HLA Ab screening and treatment on the patients’ adherence to drug therapy and their perceptions of risk to the health of the transplant. All secondary outcomes except a) and b) were assessed in the HLA Ab+ groups at the end of the intensive follow-up period (month 32 post Ab detection) and for the overall BLC vs. SC comparison was month 32 post-recruitment (Suppl Fig. S2).

### Statistical analysis

Power calculations used the observed graft failure rates reported by Lachmann et al.,^5^ hypothesizing that BLC would reduce the rate of graft failures in DSA+ patients, from 30% to 16% (HR 0.489) and in non-DSA+ recruits from 16% to 6% (HR 0.351) over 3 years. Using a variable follow up design assuming minimum follow up of 43 months, recruiting 165 patients with DSA would allow observation of 23/83 (28%) graft losses in DSA+ BLC group (B1, Suppl Fig. S1), and 39/82 (47%) in the DSA+ SC group (A1, Suppl Fig. S1). Further recruiting at least 296 patients with non-DSA would allow observation of 8/149 (5.3%) graft losses in non-DSA+ BLC group and 21/147 (14%) in the non-DSA+ SC group, providing 80% power to demonstrate superiority for these hypotheses with 2-sided 5% type 1 error.

In HLA Ab-negative groups, at least 672 patients would allow observation of 22/337 (6.5%) graft losses in SC, and 32/335 (9.5%) in BLC, providing 90% power to demonstrate non-inferiority with an assumed HR of 1.4 under the null hypothesis, and a HR of 0.63 under the alternative one-sided 95% Confidence Interval of the HR estimated using a Cox regression model. Further details have been published previously^16^ and are in the Supplementary File.

Analyses followed the OUTSMART Statistical Analysis Plan (SAP) v2.4 090221 (Supplementary File) and used the intention to treat population unless otherwise stated. All analyses were reported as treatment estimates with 95% CIs, with results considered “statistically significant” at 5% significance. No formal adjustments were made for multiple testing for the secondary outcomes as these were considered to provide contributory information only.^18^

The primary outcome of time to graft failure was modelled using Cox proportional hazards regression models, with stratification factors as covariates (site and baseline immunosuppression). Time zero (origin and entry time) was i) time of randomisation for participants who were HLA Ab+ at randomisation, and ii) time of re-screening for participants who were HLA Ab+ at re-screening. Patients follow up time was used up until the pre-COVID-19 collection period (March 2020). The proportional hazards assumption was checked (overall and across strata) by examining Kaplan Meier and Log–log survival plots and by testing whether the log-hazard ratio is constant over time (see Supplementary methods). The secondary outcomes of time to graft failure in all SC vs. BLC participants and time to death (all-cause mortality) was modelled in the same way as the primary outcome, except time zero was time of randomisation for all (Suppl Fig. S2). A sensitivity analysis was carried out examining the impact of missing data (censoring) due to death on the primary outcome results using a competing risks analysis (see Supplementary methods).

The other binary secondary outcomes (at least one report of biopsy proven rejection, infection, malignancy, de novo diabetes mellitus) were analysed using logistic regression (again adjusting for stratification factors as covariates). Proteinuria and eGFR at month 32 were analysed using a logistic (longitudinal), and linear (longitudinal) mixed effects model respectively, with all observations included between randomisation (or re-screening as appropriate) and month 32, with a random intercept for participant (assumed normally distributed). As for the primary outcome, for the within DSA+ and non-DSA+ comparisons, time at risk started at time of randomisation or time of re-screening for participants who were HLA Ab+ at re-screening. Also similarly, for the overall blinded (SC) vs. unblinded (BLC) comparison, time of randomisation was used as start of time at risk for all participants.

Further details of the main analyses are provided in the Supplementary Appendix, with Suppl Fig. S2 further explaining the different observation periods used for the different comparisons.

### Health economic analysis

This involved combining service use data with appropriate unit costs and linking costs to quality-adjusted life years (QALYs). Service use was measured with the Client Service Receipt Inventory and included primary and secondary care services.^19^ QALYs were derived from the EQ-5D-5L using area under the curve methods.^20^ Costs (excluding screening and medication) were calculated by combining the service use with appropriate unit costs. An incremental cost-effectiveness ratio was calculated to show the extra cost incurred to produce one extra QALY in the event that one group had higher costs and better outcomes.

### Adherence to drug therapy and perceptions of risk to the health of the transplant

Health surveys, consisting of validated psychological measures adapted for this specific health context, were performed at baseline and 12 and 24 months post screening for HLA Ab+, and included the Medication Adherence Report Scale (MARS) questionnaire, completed for each medication. For tacrolimus, 12 h trough levels were also compared against the target trough levels (4–8 μg/L) and a composite adherence scale based on combining MARS scores with trough levels was developed. Concern about the risk of transplant failure was measured using the Brief Illness Perceptions Questionnaire (BIPQ).^21^

Analysis of questionnaires was performed separately to the main trial data by the team at UCL. Mann Whitney U or Chi Squared tests were used to compare scores or proportions across patients in the DSA+ BLC compared to DSA+ SC groups, and non-DSA+ BLC compared to non-DSA+ SC groups. Further details can be found in the Supplementary Appendix.

### Role of the funding source

The funder of the study had no role in study design, data collection, data analysis, data interpretation, or writing of the report.

## Results

**Fig. 1:**
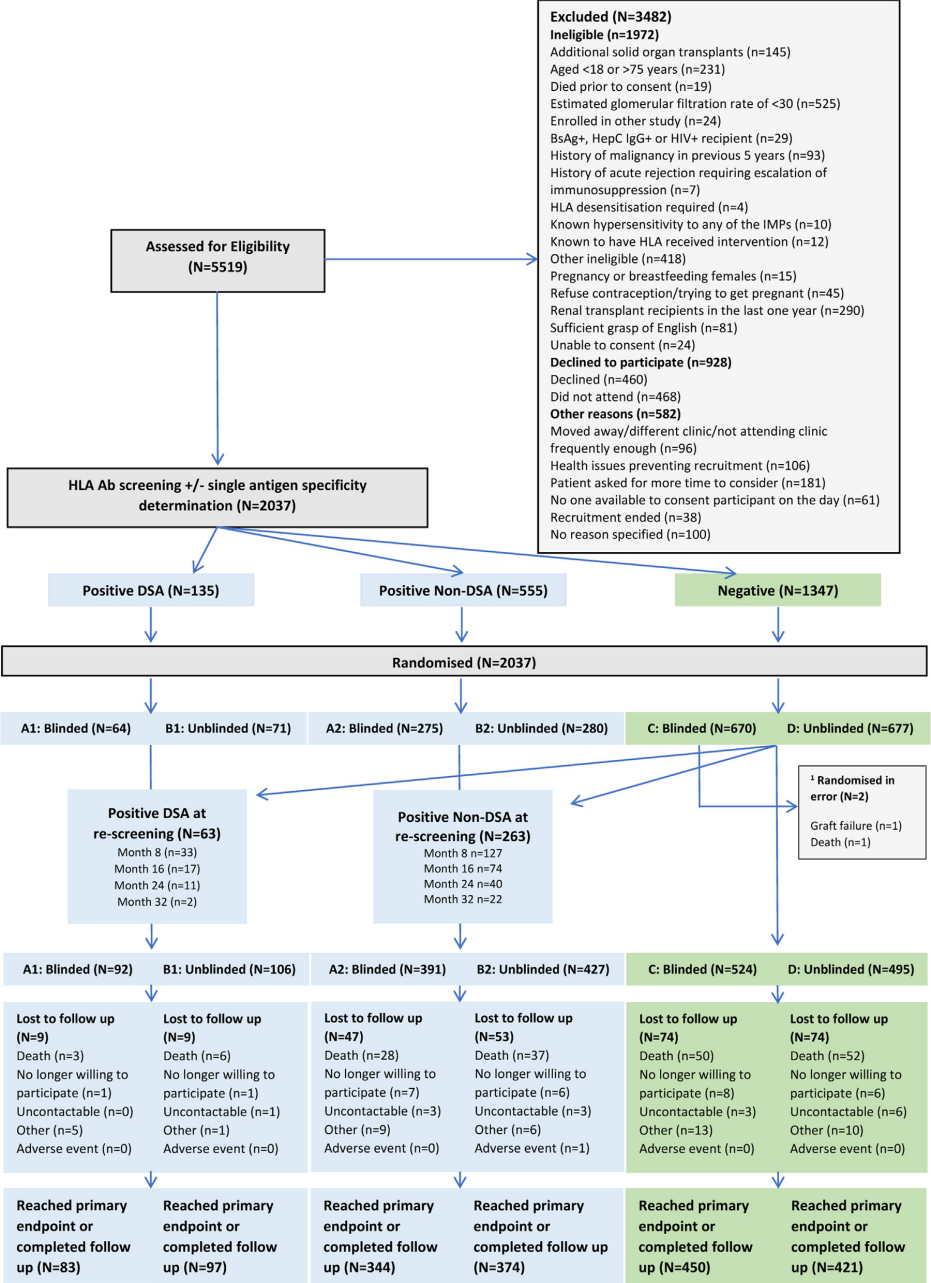
CONSORT diagram for OuTSMART. ^1^ Two patients were randomised in error to blinded HLA-Ab-negative standard care group. These were excluded from the intention to treat analysis. All other participants were included in the analysis. Refer to Supplementary File for further information.

Between 11th September 2013 and 27th October 2016, 5519 renal transplant recipients (Fig. 1 and Supplementary File), representing approximately 50% of the prevalent population available (Suppl. Table S1), were assessed for eligibility of which 2094 were enrolled. Reasons for non-enrolment are shown in Fig. 1. Fifty seven were found to be ineligible after post-consent checks so 2037 were randomised. Two patients from the HLA Ab-negative group subsequently found to be ineligible were excluded from the analysis (Fig. 1). There was generally good balance in baseline characteristics between Ab+ and Ab-groups (Table 1). The DSA+ BLC group had a higher proportion of males, longer time from transplant and higher proportion with previous transplants. Screening of the HLA Ab-negative groups finished in June 2017, at which time a further 63 with DSA (28 SC, 35 BLC) and 263 non-DSA (116 vs. 147) were identified, leaving 1019 who remained HLA Ab-negative through screening (524 vs. 495). There were no obvious imbalances in baseline variables after re-screening (Table 1).

**Table 1:** Characteristics of recruits in the six groups at point of randomisation and after all rounds of HLA antibody re-screening.

Group	DSA+				Non-DSA+				No HLA Ab				Total (at Randomisation)
	Blinded (SC)		Unblinded (BLC)		Blinded (SC)		Unblinded (BLC)		Blinded (SC)		Unblinded (BLC)		
	A1^a^		B1		A2		B2		C		D		
	Randomisation (N = 64)	Post-Screening^b^ (N = 92)	Randomisation (N = 71)	Post-Screening (N = 106)	Randomisation (N = 275)	Post-Screening (N = 391)	Randomisation (N = 280)	Post-Screening (N = 427)	Randomisation (N = 670)	Post-Screening (N = 526)	Randomisation (N = 677)	Post-Screening (N = 495)	
Age (years) *Mean (SD)*	49.5 (12.0)	48.1 (13.7)	47.0 (14.6)	46.8 (14.0)	50.0 (11.9)	49.4 (12.7)	50.6 (12.6)	50.3 (12.6)	50.3 (13.30)	51.1 (12.7)	50.5 (13.2)	51.0 (13.3)	50.2 (13)
Male (%)	66%	72%	80%	81%	56%	61%	59%	59%	73%	72%	72%	75%	69%
Ethnicity (%)													
Asian	9.4%	9.9%	14%	12%	13%	12%	13%	14%	11%	11%	13%	13%	12%
Black	19%	16%	14%	12%	7.6%	10%	11%	12%	11%	9.5%	9.7%	8.7%	10%
White	69%	72%	70%	74%	76%	74%	72%	71%	75%	76%	75%	76%	74%
Mixed	1.1%	1.1%	0%	0%	1.5%	1.5%	1.4%	0.9%	0.6%	0.4%	0.1%	0.2%	0.7%
Other	1.6%	1.1%	1.4%	1.9%	2.5%	2.0%	1.8%	1.9%	2.4%	2.9%	2.5%	2.6%	2.3%
Site [N (%)]^c^													
Leeds	8 (2.7%)	11 (3.8%)	8 (2.7%)	12 (4.1%)	41 (14%)	70 (24%)	40 (14%)	76 (26%)	96 (33%)	64 (22%)	98 (34%)	58 (20%)	291 (14%)
Royal London	6 (4.6%)	8 (6.2%)	5 (3.8%)	8 (6.2%)	11 (8.5%)	17 (13%)	12 (9.2%)	18 (14%)	48 (37%)	40 (31%)	48 (37%)	39 (30%)	130 (6.4%)
Guy's	21 (4.0%)	32 (6.0%)	24 (4.5%)	34 (6.4%)	69 (13%)	105 (20%)	72 (14%)	121 (23%)	170 (32%)	123 (23%)	173 (33%)	114 (22%)	529 (26%)
Manchester	8 (2.6%)	12 (3.8%)	8 (2.6%)	9 (2.9%)	44 (14%)	50 (16%)	47 (15%)	54 (17%)	103 (33%)	93 (30%)	102 (33%)	94 (30%)	312 (15%)
Birmingham	3 (1.4%)	5 (2.3%)	2 (0.9%)	12 (5.5%)	31 (14%)	47 (22%)	27 (12%)	42 (19%)	77 (36%)	59 (27%)	77 (36%)	52 (24%)	217 (11%)
King's College Hospital	6 (4.2%)	8 (5.6%)	4 (2.8%)	5 (3.5%)	21 (15%)	29 (20%)	21 (15%)	28 (20%)	44 (31%)	34 (24%)	47 (33%)	39 (27%)	143 (7.0%)
York	2 (3.8%)	4 (7.5%)	2 (3.8%)	4 (7.5%)	6 (11%)	9 (17%)	7 (13%)	16 (30%)	18 (34%)	13 (25%)	18 (34%)	7 (13%)	53 (2.6%)
Coventry	0 (0.0%)	0 (0.0%)	1 (1.9%)	2 (3.8%)	6 (11%)	8 (15%)	7 (13%)	12 (23%)	18 (34%)	16 (30%)	21 (40%)	15 (28%)	53 (2.6%)
Preston	1 (1.5%)	2 (3.1%)	4 (6.2%)	5 (7.7%)	11 (17%)	13 (20%)	8 (12%)	12 (19%)	21 (32%)	18 (28%)	20 (31%)	15 (23%)	65 (3.2%)
Salford	1 (1.9%)	1 (1.9%)	1 (1.9%)	1 (1.9%)	6 (12%)	8 (15%)	8 (15%)	8 (15%)	19 (37%)	17 (33%)	17 (33%)	17 (33%)	52 (2.6%)
Bradford	3 (6.2%)	3 (6.2%)	5 (10%)	7 (15%)	8 (17%)	8 (17%)	9 (19%)	12 (25%)	12 (25%)	12 (25%)	11 (23%)	6 (13%)	48 (2.4)
Royal Free	5 (4.0%)	5 (4.0%)	6 (4.8%)	6 (4.8%)	18 (14%)	24 (19%)	19 (15%)	22 (18%)	38 (30%)	32 (26%)	39 (31%)	36 (24%)	125 (6.1)
St Helier	0 (0.0%)	1 (5.3%)	1 (5.3%)	1 (5.3%)	3 (16%)	3 (16%)	3 (16%)	6 (32%)	6 (32%)	5 (26%)	6 (32%)	3 (16%)	19 (0.9%)
Cause of renal failure [N (%)]													
DM	4 (6.9%)	5 (6.0%)	2 (3.4%)	7 (8.0%)	7 (2.9%)	17 (5.1%)	13 (5.4%)	22 (5.9%)	38 (6.7%)	27 (6.0%)	40 (6.8%)	26 (6.1%)	104 (6.0%)
GN	22 (38%)	28 (34%)	19 (33%)	30 (34%)	93 (39%)	128 (38%)	94 (39%)	147 (40%)	216 (38%)	175 (39%)	224 (38%)	160 (38%)	668 (38%)
PKD	7 (12%)	10 (12%)	9 (16%)	12 (14%)	32 (13%)	45 (14%)	34 (14%)	54 (15%)	105 (19%)	89 (20%)	100 (17%)	77 (18%)	287 (16%)
Hypertension	6 (10%)	7 (8.4%)	6 (10%)	7 (8.0%)	20 (8.3%)	28 (8.4%)	22 (9.2%)	34 (9.2%)	43 (7.6%)	34 (7.6%)	47 (8.0%)	34 (8.0%)	144 (8.2%)
Congenital	7 (12%)	13 (16%)	7 (12%)	10 (11%)	31 (13%)	41 (12%)	22 (9.2%)	34 (9.2%)	66 (12%)	50 (11%)	47 (8.0%)	32 (7.6%)	180 (10%)
Obstructive	8 (14%)	12 (15%)	10 (17%)	16 (18%)	38 (16%)	50 (15%)	34 (14%)	48 (13%)	54 (9.5%)	38 (8.5%)	80 (14%)	60 (14%)	224 (13%)
Other	4 (6.9%)	8 (9.6%)	5 (8.5%)	6 (6.7%)	19 (7.8%)	25 (7.5%)	20 (8.3%)	31 (8.4%)	45 (8.1%)	35 (7.7%)	46 (7.9%)	34 (7.9%)	139 (8.1%)
Previous transplants [N (%)]													
0	48 (76%)	71 (78%)	52 (73%)	85 (80%)	193 (71%)	301 (77%)	198 (71%)	337 (79%)	613 (92%)	482 (92%)	633 (94%)	461 (94%)	1737 (86%)
1	12 (19%)	17 (19%)	18 (25%)	20 (19%)	71 (26%)	79 (20.%)	65 (23%)	73 (17%)	55 (8.2%)	42 (8%)	35 (5.2%)	25 (5.1%)	256 (12%)
2	3 (4.8%)	3 (3.3%)	1 (1.4%)	1 (0.9%)	8 (2.9%)	8 (2.1%)	13 (4.7%)	13 (3.1%)	0 (0%)	0 (0%)	5 (0.7%)	5 (1.0%)	30 (1.5%)
3	0 (0%)	0 (0%)	0 (0%)	9 (0%)	1 (0.4%)	1 (0.3%)	3 (1.1%)	3 (0.7%)	0 (0%)	0 (0%)	0 (0%)	0 (0%)	4 (0.2%)
Time (years) since Tx Median (IQR)	6.6 (3.0–12.0)	5.9 (3.0–11.9)	9.7 (3.9–14.3)	6.7 (3.0–12.4)	5.7 (2.2–10.9)	5.4 (2.2–9.8)	4.9 (2.3–11.2)	5.1 (2.4–10.8)	5.4 (2.4–9.2)	5.4 (2.4–9.6)	5.1 (2.4–9.7)	5.1 (2.4–9.8)	5.4 (2.4–10.3)
Immunosuppression													
CsA [N (%)]	17 (27%)	26 (28%)	18 (25%)	22 (21%)	49 (18%)	69 (18%)	49 (18%)	74 (17%)	121 (18%)	90 (17%)	120 (18%)	89 (18%)	374 (18%)
Mean Dose [mg (SD)]	170.3 (49.8)	187.3 (62.8)	199.4 (68.5)	199.6 (63.6)	168.6 (65.0)	174.4 (62.5)	168.7 (60.4)	160.6 (58.9)	180.7 (67.9)	176.3 (67.8)	168.7 (63.0)	174.7 (62.9)	174.1 (64.4)
Mean trough level [μg/L (SD)]	72.3 (34.8)	89.3 (56.2)	80.9 (55.3)	80.7 (51.5)	102.8 (84.8)	101.2 (79.8)	88.6 (56.1)	87.3 (52)	100 (71.4)	91.9 (52.3)	109.6 (88.5)	116.4 (97.2)	99.9 (76)
Tac [N (%)]	39 (61%)	56 (64%)	41 (58%)	67 (64%)	205 (75%)	296 (76%)	205 (73%)	313 (73%)	499 (74%)	392 (75%)	501 (74%)	366 (74%)	1490 (73%)
Mean dose [mg (SD)]	6.14 (6.72)	6.18 (5.97)	4.01 (2.24)	4.62 (3.33)	5.08 (3.51)	5.14 (3.66)	5.60 (4.60)	5.41 (3.73)	5.50 (4.12)	5.44 (4.13)	4.89 (3.65)	4.70 (3.15)	5.23 (4.02)
Mean trough level [μg/L (SD)]	6.49 (2.64)	6.56 (2.86)	5.65 (2.06)	5.83 (2.18)	6.95 (2.93)	6.88 (2.74)	6.86 (2.29)	6.68 (2.21)	6.91 (2.31)	6.93 (2.26)	6.71 (2.47)	6.72 (2.52)	6.79 (2.46)
MMF [N (%)]	40 (63%)	59 (64%)	41 (58%)	62 (59%)	177 (64%)	254 (65%)	176 (63%)	271 (63%)	460 (69%)	361 (69%)	471 (70%)	351 (71%)	1365 (67%)
Mean dose [mg (SD)]	1156 (476)	1165 (482)	1098 (422)	1145 (399)	1131 (450)	1134 (457)	1117 (483)	1112 (472)	1155 (490)	1147 (495)	1136 (466)	1136 (473)	1138 (473)
Aza [N (%)]	15 (2.0%)	19 (2.0%)	19 (7.0%)	26 (25%)	52 (19%)	66 (17%)	39 (14%)	61 (14%)	90 (13%)	71 (13%)	94 (14%)	69 (14%)	309 (15%)
Mean dose [mg (SD)]	88.3 (45.2)	90.8 (43.5)	69.7 (33.9)	76.9 (32.3)	76.7 (43.3)	78.2 (40.8)	86.5 (39.3)	88.5 (39.4)	85.3 (34.7)	85.2 (33.4)	85.1 (35.1)	83.6 (35.9)	83.1 (37.5)
Sirolimus [N (%)]^d^	2 (3.1%)	2 (2.2%)	5 (7.0%)	6 (5.7%)	10 (3.6%)	10 (2.6%)	4 (1.4%)	6 (1.4%)	17 (2.5%)	16 (3.0%)	25 (3.7%)	18 (3.6%)	63 (3.1%)
Mean dose [mg (SD)]	2.5 (0.71)	2.5 (0.71)	1.6 (0.55)	1.5 (0.55)	2 (0.82)	2 (0.82)	2 (0.82)	2 (0.89)	1.65 (0.70)	1.62 (0.72)	2 (0.91)	2.06 (0.8)	1.89 (0.81)
Prednisolone [N (%)]	37 (58%)	53 (58%)	38 (54%)	62 (59%)	153 (56%)	210 (54%)	154 (55%)	227 (53%)	369 (55%)	295 (56%)	372 (55%)	274 (55%)	1123 (55%)
Mean dose [mg (SD)]	4.97 (1.72)	5.16 (1.81)	4.97 (2.13)	5.1 (1.87)	4.99 (1.45)	5.01 (1.39)	4.99 (1.62)	5.13 (1.53)	5.08 (1.67)	5.11 (1.75)	5.2 (1.62)	5.11 (1.43)	5.08 (1.57)
Taking Tac/MMF/Pred [N (%)]	13 (20%)	19 (21%)	13 (18%)	24 (23%)	82 (30%)	114 (29%)	70 (25%)	106 (25%)	192 (29%)	152 (29%)	189 (28%)	139 (28%)	559 (23%)
Renal function Creatinine (μmol/L) [Mean (SD)]	128.97 (40.32)	129.09 (39.30)	124.96 (37.29)	126.06 (38.25)	123.23 (35.42)	124.08 (35.23)	122.61 (35.81)	121.17 (35.25)	126.17 (38.78)	126.02 (39.71)	126.73 (36.76)	129.07 (36.96)	125.52 (37.26)
eGFR (mls/min/1.73 m^2^) [Mean (SD)]	52.31 (15.36)	52.93 (15.23)	56.27 (17.70)	56.16 (18.01)	52.12 (16.54)	52.80 (16.39)	52.89 (16.32)	54.12 (17.30)	53.77 (15.90)	53.59 (15.95)	53.76 (17.26)	52.82 (16.57)	53.46 (16.55)
PCR^e^ (mg/mmol) [Median (IQR)]	26.50 (15.50–48.25)	26.50 (13.75–49.75)	16.50 (10.75–39.25)	23.50 (13.00–49.50)	18.00 (8.00–37.25)	18.00 (8.00–38.00)	20.00 (9.00–42.50)	19.00 (9.00–37.25)	17.00 (9.00–41.25)	17.00 (9.00–39.00)	21.00 (10.00–41.00)	21.00 (10.00–43.00)	19.00 (9.00–41.00)
ACR (mg/mmol) [Median (IQR)]	1.90 (1.40–1.95)	2.00 (1.90–45.60)	5.30 (2.75–7.85)	2.30 (0.80–8.00)	2.80 (1.30–6.30)	2.80 (1.20–7.70)	7.05 (3.13–15.10)	6.40 (2.82–20.10)	3.20 (1.20–9.22)	3.20 (1.35–9.22)	3.30 (0.95–10.20)	2.55 (0.90–8.75)	3.30 (1.30–9.60)

DSA = donor specific antibody; HLA = human leukocyte antigen; Ab = antibody; SC = standard care; BLC = biomarker led care; SD = standard deviation; GSTT = Guy's & St Thomas' NHS Foundation Trust; DM = Diabetes mellitus; GN = glomerulonephritis; PKD = polycystic kidney disease; IQR = interquartile range; CsA = ciclosporin; Tac = tacrolimus; MMF = mycophenolate mofetil; Aza = azathioprine; eGFR = estimated glomerular filtration rate (by 4 value Modification of Diet in Renal Disease formula); PCR = urinary protein creatinine ratio; ACR = albumin creatinine ratio.

a Group nomenclature refers to Supplementary Fig. S1.

b Post-screening refers to status following movement from HLA Ab-negative to the HLA Ab+ groups; with reference to the immunosuppression data for groups B1 and B2, this table shows values prior to optimization.

c For full names of recruiting NHS trusts see Supplementary File.

d Three patients in the blinded SC group were taking Everolimus, with a mean ± SD dose of 2.33 ± 0.58 mg/L. These patients are not included here.

e According to centre preference, patients had either PCR or ACR measured, not both.

HLA Ab status at time of transplant was known for 1863/2037 (91%) recruits. From this we can infer that >75% recruits in DSA+ groups had developed de novo DSA (Table 2). Approximately 45% of recruits had DSA directed against HLA DQB, and 15–26% had DSA against HLA A (Table 3). 43 of 389 (11%) non-DSA+ SC recruits and 42 of 425 (9.8%) non-DSA+ BLC recruits were classified as non-DSA+ because of insufficient information on donor-recipient mismatches (Table 2).

**Table 2:** HLA Ab status.

	DSA+						Non-DSA+						No HLA Ab					
	Blinded (SC) A1			Unblinded (BLC) B1			Blinded (SC) A2			Unblinded (BLC) B2			Blinded (SC) C			Unblinded (BLC) D		
	Time of Tx	Post-screening	End	Time of Tx	Post-screening	End	Time of Tx	Post-screening	End	Time of Tx	Post-screening	End	Time of Tx	Post-screening	End	Time of Tx	Post-screening	End
Number of Ab+ (%)	21 (22.8)	92 (100)	34 (37∗)	23 (21.7)	106 (100)	38 (35.8)	158 (40.4)	389 (99.5)	106 (27.1)	153 (35.8)	425 (99.5)	99 (23.1)	37 (7)	2 (0.4)	9 (1.7)	33 (6.7)	0 (0)	8 (1.6)
*Definite DSA*	–	*91 (98.9)*	*18 (19.6)*	*–*	*103 (97.2)*	*25 (23.6)*	*–*	*0 (0)*	*13 (3.3)*	*–*	*0 (0)*	*3 (0.7)*	–	*0 (0)*	*2 (0.4)*	–	*0 (0)*	*1 (0.2)*
*Definite non-DSA*	–	*1*^a^*(1.1)*	*13 (14.1)*	*–*	*1*^a^*(0.9)*	*10 (9.4)*	*–*	*346 (88.5)*	*86 (22)*	*–*	*383 (89.7)*	*92 (21.5)*	–	*0 (0)*	*7 (1.3)*	*–*	*0 (0)*	*5 (1)*
*Unknown whether DSA*	–	*0 (0)*	*3 (3.3)*	*–*	*2*^a^*(1.8)*	*3 (2.8)*	–	*43 (11)*	*7 (1.8)*	*–*	*42 (9.8)*	*4 (0.9)*	–	*2*^b^*(0.6)*	*0 (0)*	*–*	*0 (0)*	*2 (0.4)*
Number of Ab – (%)	64 (69.6)	0 (0)	35 (38)	72 (67.9)	0 (0)	40 (37.7)	192 (49.1)	0	191 (48.8)	230 (53.9)	1^c^ (0.2)	210 (49.2)	449 (85.4)	521 (99)	437 (83.1)	431 (87.1)	489 (98.8)	402 (81.2)
Missing data	7 (10.5)	0 (0)	23 (25)	11 (10.4)	0 (0)	28 (26.4)	41 (10.5)	2 (0.5)	94 (24)	44 (10.3)	1 (0.2)	118 (27.6)	40 (7.6)	3 (0.6)	80 (15.2)	31 (6.3)	6 (1.2)	85 (17.2)
Total	92 (100)	92 (100)	92 (100)	106 (100)	106 (100)	106 (100)	391 (100)	391 (100)	391 (100)	427 (100)	427 (100)	427 (100)	526 (100)	526 (100)	526 (100)	495 (100)	495 (100)	495 (100)

Patients grouped according to post-screening HLA Ab status; table shows details of their HLA Ab status at time of transplant and end of trial, and how their trial antibody status was classified. HLA Ab classified as ‘uncertain whether DSA’ were classified this way when not enough data on donor recipient mismatches was available: these HLA Ab were included in non-DSA groups for analysis.

[NB, during re-screening of HLA Ab-negative groups, upon becoming antibody positive, recruits not tested again until ‘end’ (= last trial visit)].

a These patients were incorrectly identified as having DSA—refer to Supplementary File for details.

b These patients developed Ab at the second round of screening (month 16) but were incorrectly maintained in the HLA Ab-negative group by mistake.

c This patient was incorrectly randomised as having a non-DSA antibody.

**Table 3:** HLA mismatches, and type and specificities of DSA.

HLA	DSA+				Non-DSA+				No HLA Ab-			
	Blinded (SC) A1 (N = 92)		Unblinded (BLC) B1 (N = 106)		Blinded (SC) A2 (N = 391)		Unblinded (BLC) B2 (N = 427)		Blinded (SC) C (N = 526)		Unblinded (BLC) D (N = 495)	
	Donor MM N (%): ∗assumed	DSA % [median MFI]	Donor MM N (%) ∗assumed	DSA % [median MFI]	Donor MM N (%) ∗assumed	DSA % [median MFI]	Donor MM N (%) ∗assumed	DSA % [median MFI]	Donor MM N (%) ∗assumed	DSA % [median MFI]	Donor MM N (%) ∗assumed	DSA % [median MFI]
A	78 (85%) ∗0	26% [3998]	87 (82%) ∗1	15% [3630]	273 (70%) ∗0	0	285 (67%) ∗0	0	402 (76%) ∗0	0	352 (71%) ∗0	0
B	84 (91%) ∗0	9.8% [2424]	93 (88%) ∗0	13% [5990]	287 (73%) ∗0	0	300 (70%) ∗0	0	435 (83%) ∗0	0	385 (78%) ∗1	0
C	73 (79%) ∗1	16% [3733]	80 (76%) ∗2	10% [3401]	246 (63%) ∗1	0	258 (60%) ∗1	0	364 (69%) ∗3	0	322 (65%) ∗1	0
DRB1	65 (71%) ∗1	6.5% [2645]	89 (84%) ∗7	18% [3155]	200 (51%) ∗2	0	220 (52%) ∗2	0	307 (58%) ∗1	0	284 (57%) ∗2	0
DRB3	16 (17%) ∗3	4.3% [3148]	17 (16%) ∗2	3.8% [4290]	38 (9.7%) ∗0	0	35 (8.2%) ∗0	0	58 (11%) ∗0	0	65 (13%) ∗2	0
DRB4	17 (19%) ∗0	2.2% [13850]	25 (24%) ∗1	7.5% [6373]	42 (11%) ∗1	0	49 (12%) ∗2	0	58 (11%) ∗0	0	82 (17%) ∗0	0
DRB5	7 (7.6%) ∗0	1.1% [5326^1^]	8 (7.5%) ∗0	0.94% [5568^1^]	28 (7.2%) ∗0	0	28 (6.6%) ∗0	0	50 (9.5%) ∗0	0	49 (9.9%) ∗1	0
DQA	8 (8.7%) ∗2	4.3% [12005]	9 (8.5%) ∗6	4.7% [12845]	9 (2.3%) ∗5	0	14 (3.3%) ∗2	0	10 (1.9%) ∗2	0	8 (1.6%) ∗1	0
DQB	66 (72%) ∗5	44% [6947]	78 (74%) ∗5	46% [6279]	161 (41%) ∗5	0	189 (44%) ∗6	0	281 (53%) ∗0	0	265 (54%) ∗4	0
DPB	11 (12%) ∗5	3.3% [5623]	9 (8.5%) ∗4	0.94% [5177^a^]	31 (7.9%) ∗15	0	34 (8%) ∗15	0	35 (6.7%) ∗6	0	33 (6.7%) ∗18	0

Table shows the number and percentage of recruits in each group with a mismatched (MM) HLA on their donor kidney, according to the type of mismatch, HLA class I (A–C) or HLA class II (DRB1-5, DQA, DQB or DPB).

In some cases, a mismatch at 1 locus can be assumed by the strong linkage disequilibrium with other loci. The number of recruits where this happens is represented by ∗. Also shown is the percentage of recruits in each group with a DSA against these HLA, followed by the median mean fluorescence intensity (MFI) of the DSA on Luminex analysis using single antigen beads. More recruits in the blinded group had antibodies against HLA A, whereas more recruits in the unblinded group had antibodies against HLA DRB1 and DRB4.

a Only one observation for these values and therefore median is just the observed value.

The majority of recruits were taking tacrolimus (73%) or MMF (67%) and prednisolone (55%) at randomisation; 27% were taking all 3 drugs (Table 1). Baseline immunosuppression use was balanced across groups (Table 1). 515/532 (97%) HLA Ab+ BLC recruits adhered to the intervention, which was defined as having an optimisation interview. 33% with DSA, and 24% with non-DSA underwent steroid boost (Table 4). The proportion taking each of the IMPS increased in the HLA Ab+ BLC groups. For instance, in the BLC DSA+ group, the proportion taking tacrolimus rose from 64% at time of DSA detection to 82% at the last visit, those taking MMF rose from 59% to 73%, and those taking prednisolone rose from 59% to 76%. The proportion taking all three IMPs increased from 23% immediately post-screening to 50% immediately post-optimisation in the DSA+ BLC group, and from 25% to 42% in the non-DSA+ BLC group. These changes were sustained to the last visit and all were statistically significant (Table 4). There were no changes in proportions taking either tacrolimus, MMF or prednisolone, or all three drugs in the SC groups (Table 4) post-screening to the last visit. The number and proportion taking various combinations of drugs in the BLC HLA Ab+ groups is shown In Suppl Table S2. Table 1, Table 4 and Suppl Table S2 contain detailed information on drug doses and trough levels in each of the groups at enrolment, post-screening, and at the end. The last person, last visit occurred in March 2020, with the remote primary endpoint collection moved as described above.

**Table 4:** Optimisation of IS summary.

Group	DSA+		Non-DSA+		HLA Ab Negative	
	Blinded (SC)	Unblinded (BLC)	Blinded (SC)	Unblinded (BLC)	Blinded (SC)	Unblinded (BLC)
	A1 (N = 92)	B1 (N = 106)^d^	A2 (N = 391)	B2 (N = 427)^e^	C (N = 526)	D (N = 495)
Had Optimization interview N (%)	0 (0%)	102 (96%)	0 (0%)	413 (97%)	0 (0%)	0 (0%)
*Taking Tac*						
***Post-screening***^a^***N (%****)*	56 (61%)	68 (64%)	296 (76%)	313 (73%)	392 (75%)	366 (74%)
*Mean dose mg (SD)*	6.2 (6)	4.6 (3.3)	5.1 (3.7)	5.4 (4.4)	5.4 (4.1)	4.7 (3.2)
*Mean level (SD)*	6.6 (2.9)	5.8 (2.2)	6.9 (2.7)	6.7 (2.2)	6.9 (2.3)	6.7 (2.5)
***At last visit***^b^***N (%)***	58 (63%)	87 (82%)	301 (77%)	355 (85%)	387 (74%)	368 (74%)
*Mean dose mg (SD)*	6.2 (4.4)	5.2 (3.7)	4.8 (3.3)	5.2 (3.7)	5.1 (3.8)	4.6 (3.2)
*Mean level (SD)*	6.6 (2.6)	6.8 (2.4)	6.5 (2.3)	6.7 (2.3)	6.6 (2.2)	6.4 (2.0)
*Taking MMF*						
***Post-screening***^a^***N (%****)*	59 (64%)	62 (59%)	254 (65%)	271 (63%)	361 (69%)	351 (71%)
*Mean dose mg (SD)*	1165 (482)	1145 (399)	1134 (457)	1112 (472)	1147 (495)	1136 (473)
***At last visit***^b^***N (%)***	59 (64%)	77 (73%)	246 (63%)	305 (72%)	246 (63%)	338 (68%)
*Mean dose mg (SD)*	1178 (470)	1237 (450)	1082 (442)	1149 (457)	1088 (440)	1098 (438)
*Taking Prednisolone*						
***Post-screening***^a^***N (%****)*	53 (58%)	62 (59%)	210 (54%)	227 (53%)	295 (56%)	274 (55%)
*Mean dose mg (SD)*	5.2 (1.8)	5.1 (1.9)	5.0 (1.4)	5.1 (1.6)	5.1 (1.8)	5.1 (1.4)
***At last visit***^b^***N (%)***	55 (60%)	81 (76%)	212 (54%)	268 (63%)	303 (58%)	273 (55%)
*Mean dose mg (SD)*	5.7 (3.7)	5.3 (2.1)	5.2 (1.9)	5.2 (1.8)	5.7 (4.2)	5.1 (1.5)
*Given Prednisolone boost N (%)*	0 (0%)	34 (33%)	0 (0%)	101 (24%)	0 (0%)	0 (0%)
*Taking Tac/MMF/Pred N (%)*						
*Post-screening*	19 (21%)	24 (23%)	114 (29%)	106 (25%)	152 (29%)	139 (28%)
*Immediately post-optimization*^c^	–	53 (54%)	–	178 (44%)	–	–
*At last visit*^b^	20 (22%)	51 (48%)	114 (29%)	172 (40%)	142 (27%)	129 (26%)

Table shows the number and proportion of patients in each group who were given/taking aspects of the optimisation process, as well as the average doses of each drug.

a For HLA Ab+ patients this is the time point immediately after Ab+ status identified (post-randomisation if Ab+ at recruitment or post-re-screening if Ab-negative at recruitment).

b At the last intensive follow-up visit (up until 32 months, or potentially 64 months for rescreens) that the participant attended.

c Percentages are out of those participants who had non-missing immunosuppression data immediately post-optimisation (98 in group B1, 405 in group B2).

d McNemar's test for change in use over time (all increases) in DSA+ unblinded (BLC) group: increase in proportion taking Tac (p < 0.001), MMF (p = 0.02) and Prednisolone (p < 0.001) as well as taking all three drugs (p < 0.001) from post-screening to last visit.

e As for^d^: McNemar's test for non-DSA+ unblinded (BLC) group: increase in proportion taking Tac (p < 0.001), MMF (p < 0.001) and Prednisolone (p < 0.001) as well as taking all three drugs (p < 0.001) from post-screening to last visit.

**Fig. 2:**
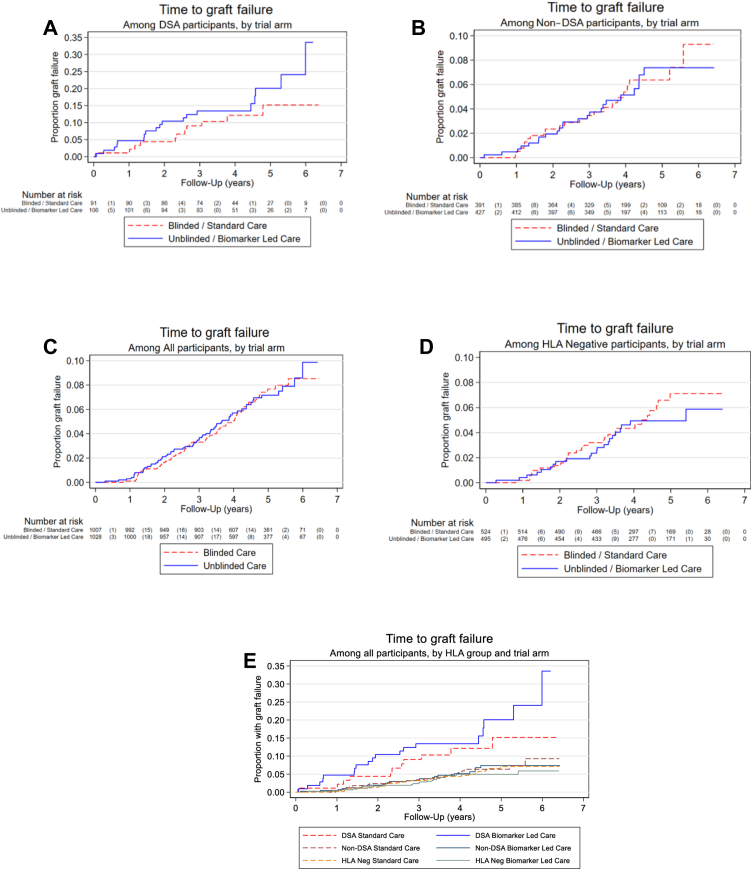
Kaplan Meier curves comparing time to graft failure in the DSA+ groups (A), non-DSA+ groups (B), all biomarker led care (BLC) vs. all standard care (SC) participants (C), HLA Ab neg groups (D) and all 6 groups (E). In A-D, Blue (unbroken) line = patients in unblinded, BLC arm. Red (broken) line = patients in blinded SC arm. The number at risk of graft failure at each time point is shown beneath the graph, followed by (in brackets) the number of graft failures. NB. One HLA-Ab-negative participant in the blinded (SC) group who developed DSA on re-screening was not included in this analysis as the graft failed prior to re-screening, so they were not at risk for the purpose of this analysis.

Median follow up time was 3.9 years (3.2–5.1 IQR) for the DSA+ groups comparison, 3.9 years (3.3–5.1 IQR) in the non-DSA+ comparison, and 4.4 years (3.5–5.3 IQR) in the overall comparison. There were 34 graft failures in the HLA Ab+ groups within the SC arm (12 DSA+, 22 non-DSA+) compared to 42 in BLC (19 DSA+, 23 non-DSA+). There was no evidence for superiority of the unblinded BLC strategy compared to the SC strategy, with 95% CIs that included the null hazard ratio, in the DSA+ group [ HR 1.54 (95% CI 0.72 to 3.30)] or non-DSA+ group [HR 0.97 (0.54–1.74)] (Fig. 2A and B, Table 5) and these effect sizes were not considered clinically significant. None of the pre-planned sensitivity analyses showed any appreciable differences from the primary analysis (Suppl Table S3). Post-hoc sensitivity analyses adjusting additionally for factors considered unbalanced at baseline (sex and time since transplant), or only looking at unblinded BLC recruits that underwent ‘best’ optimisation had no impact on the effect estimates (Suppl Table S3).

**Table 5:** Primary and secondary outcome results.

Group/Comparison		95% CI	P-value
**Primary Outcome – Time to Graft Failure**	Hazard Ratio		
DSA (N = 197^a^)	1.54	0.72–3.30	0.27
Non-DSA (N = 818)	0.97	0.54–1.74	0.91
All participants (N = 2035^b^)	1.02	0.72–1.44	0.93
**Secondary Outcome measures**			
**Death**	Hazard Ratio		
DSA (N = 197)	2.33	0.57–9.57	0.24
Non-DSA (N = 818)	1.24	0.76–2.02	0.40
All participants (N = 2035)	1.14	0.85–1.54	0.38
**Biopsy Proven rejection**	Odds Ratio^c^		
DSA (N = 198)	0.35	0.10–1.17	0.09
Non-DSA (N = 818)	0.57	0.18–1.78	0.32
All participants (N = 2035)	0.50	0.27–0.94	0.03
**Confirmed infection**	Odds Ratio^c^		
DSA (N = 197)	1.75	0.89–3.44	0.10
Non-DSA (N = 809)	1.09	0.79–1.50	0.62
All participants (N = 2010)	1.08	0.88–1.33	0.46
**Malignancy**	Odds Ratio^c^		
DSA (N = 198)	1.08	0.36–3.28	0.89
Non-DSA (N = 810)	0.93	0.57–1.52	0.77
All participants (N = 2015)	0.92	0.65–1.31	0.65
**Diabetes Mellitus**	Odds Ratio^c^		
DSA (N = 198)	0.99	0.19–5.21	0.99
Non-DSA (N = 818)	0.56	0.25–1.26	0.16
All (N = 2015)	0.75	0.41–1.37	0.34
**Proteinuria**	Odds Ratio^c^		
DSA (N = 184)	0.28	0.05–1.59	0.15
Non-DSA (N = 788)	1.47	0.61–3.53	0.39
All participants (N = 1972)	0.80	0.47–1.37	0.42
**eGFR**	**Mean difference**		
DSA (N = 192)	0.91	−2.83–4.65	0.63
Non-DSA (N = 805)	0.24	−1.50–1.98	0.78
All participants (N = 2015)	−0.46	−1.98–1.05	0.55

Table compares primary and secondary outcome measures in patients with either DSA, non-DSA or all patients in the unblinded BLC group vs. those in blinded SC group.

a One HLA-Ab-negative participant in the blinded (SC) group who developed DSA on re-screening was not included in this analysis as the graft failed prior to re-screening, so they were not at risk for the purpose of this analysis.

b Although 2037 randomised, 2 patients in the HLA Ab-negative group were excluded from the analysis—see text and Fig. 2.

c Where secondary outcomes report odds ratios, we have also reported risk differences from the same models in Supplementary Table S8 to aid interpretation.

Overall there were 62 graft failures in the SC arm (including 28 HLA Ab-negatives) compared to 64 in BLC (including 22 in the HLA Ab-negative groups), providing insufficient evidence for non-inferiority of the unblinded BLC strategy with the upper 95% confidence limit for the hazard ratio exceeding the pre-specified threshold of 1.4 (HR 1.02, 95% CI 0.72 to 1.44) (Fig. 2C). Time to graft failure in the HLA Ab-negative groups only is shown in Fig. 2D and time to graft failure in all participants split by group is shown in Fig. 2E.

Patient survival was 92.7% in the SC arm and 92.2% in the BLC arm with no significant differences between arms in any of the specified comparisons (Table 5).

The number of recruits with biopsy-proven rejection is shown in Table 6. The odds of biopsy proven rejection were lower in both DSA+ BLC and non-DSA+ BLC, compared to the respective SC groups but were not significant (Table 6). However, the odds of biopsy proven rejection in the whole BLC arm were lower than in SC (0.5, 95% CI 0.27 to 0.94) and this was statistically significant (p = 0.03). The diagnostic features of all biopsies performed in the DSA+ patients are reported in Suppl Table S4.

**Table 6:** Biopsy proven rejection, confirmed infections, malignancies and diabetes mellitus.

	DSA+		Non-DSA+		HLA Ab-negative		Total
	SC (N = 92)	BLC (N = 106)	SC (N = 391)	BLC (N = 427)	SC (N = 525)	BLC (N = 495)	
**Biopsy proven rejection**							
Total Biopsy proven rejection	11	5	8	6	12	5	47
*Included in formal analysis of rejection in HLA Ab*+ *groups*^a^	*9 (9.8%)*	*4 (3.8%)*	*8 (2.0%)*	*5 (1.2%)*	*NA*	*NA*	*26*
*Included in formal analysis of rejection in overall BLC vs. SC comparison*^b^	*10 (10.9%)*	*5 (4.7%)*	*7 (1.8%)*	*5 (1.2%)*	*12 (2.3%)*	*5 (1.0%)*	*44*
**Culture/PCR confirmed infections**							
Total confirmed infections	21	32	95	109	115	107	479
*Included in formal analysis of infection in HLA Ab*+ *groups*^a^	*18 (19.6%)*	*32 (30.2%)*	*92 (23.5%)*	*106 (24.8%)*	*NA*	*NA*	*248*
*Included in formal analysis of infection in overall BLC vs. SC comparison*^b^	*21 (22.8%)*	*32 (30.2%)*	*87 (22.3%)*	*100 (23.4%)*	*115 (21.9%)*	*107 (21.6%)*	*462*
**Malignancies**							
Total malignancies	6	10	35	38	36	25	150
*Included in formal analysis of malignancies in HLA Ab*+ *groups*^a^	*6 (6.5%)*	*8 (7.5%)*	*35 (9.0%)*	*37 (8.7%)*	*NA*	*NA*	*86*
*Included in formal analysis of malignancies in overall BLC vs. SC comparison*^b^	*5 (5.4%)*	*10 (9.4%)*	*29 (7.4%)*	*31 (7.3%)*	*36 (6.9%)*	*25 (5.0%)*	*136*
**Diabetes Mellitus**							
Total Diabetes	4	3	17	10	7	9	50
*Included in formal analysis of diabetes in HLA Ab*+ *groups*^a^	*3 (3.3%)*	*3 (2.8%)*	*16 (4.1%)*	*10 (2.3%)*	*NA*	*NA*	*32*
*Included in formal analysis of diabetes in overall BLC vs. SC comparison*^b^	*3 (3.3%)*	*3 (2.8%)*	*15 (3.8%)*	*7 (1.6%)*	*7 (1.3%)*	*9 (1.8%)*	*44*

Table shows total number of biopsy-proven rejections, confirmed infections and malignancies. Total events were defined as those recorded across the whole period of intensive follow-up, which was at least 32 months for everyone, but for some who had ‘clock reset’ was longer, up to 64 months.

a The formal analysis of secondary endpoints for the HLA Ab+ groups excluded events that occurred prior to developing the HLA Ab in recruits that entered the trial as HLA Ab-negative (i.e. prior to rescreening). Refer to Supplementary Fig. S2.

b The formal analysis of secondary endpoints for the overall comparison of BLC vs. SC outcomes excluded events that occurred beyond 32 months in all recruits, irrespective of HLA Ab status or ‘clock reset’. Refer to Supplementary Fig. S2. For calculation of percentages, the denominator is the number within each of the groups.

There were no statistically significant or clinically significant differences between groups for any adverse effects recorded as secondary outcomes (Table 5). The number of proven infections in each group is shown in Table 6 and details of specific infections are in Suppl Table S5. The number of malignancies in each group is shown in Table 6 and details of specific malignancies are given in Suppl Table S6. The number of cases of diabetes mellitus in each group is shown in Table 6.

The odds of developing proteinuria in DSA+ BLC group were 0.28 times the odds of developing proteinuria in the DSA+ SC group but the confidence intervals were wide and included the null value. Mean eGFR at Month 32 was similar between the BLC DSA+ group (53.1 SD = 19.8) and SC DSA+ group (56.1 SD = 22.7) and there was no significant difference in mean eGFR for any of the comparisons (Table 5). Descriptions of all other AEs, changes in DSA at the final visit and outcomes associated with these changes are reported in Table 2 and the Supplementary File.

Of the HLA Ab+ cases, health economic data were available for 173 SC and 189 BLC recruits. The mean healthcare costs for 12 months prior to baseline assessment were £2287 for BLC group and £3600 for SC group. In the 12 months up to the 16-month follow-up the, mean costs were £3137 and £1672 respectively. Adjusting for baseline, the mean costs for the BLC group were £1522 higher than for the SC group at follow-up but this was not statistically significant (95% CI, -£839 to £3883). Over the follow-up period, the BLC group accrued 1.07 QALYs and the SC group accrued 1.05 QALYs. Adjusting for baseline quality of life, the BLC group gained 0.00 more QALYs (2 decimal places) which was not statistically significant (95% CI, −0.02 to 0.02). The incremental cost-effectiveness ratio was £1,778,245 per QALY for BLC compared to SC. This is substantially higher than the threshold used by the National Institute for Health and Care Excellence in England and Wales to determine cost-effectiveness.

Self-reported adherence, assessed by MARS was no different at any time point for tacrolimus in HLA Ab+ patients in BLC vs. SC arms (Table 7). Assessment of adherence based on tacrolimus levels only suggested better adherence at 12 months in the DSA+ BLC group compared to the DSA+ SC group (p = 0.02), though this was non-significant when using a composite score combining MARS with tacrolimus levels. In contrast, self-reported adherence at 12 months was significantly higher in the DSA+ BLC group for both MMF (p = 0.03) and prednisolone (p = 0.04) than in the DSA+ SC group (Table 3). There were no significant differences across any treatment or screening groups on self-reported concern about the risk of transplant failures.

**Table 7:** The impact of biomarker screening and treatment on patients’ adherence to drug therapy and their concern about risk of transplant failure.

	DSA+				Comparison	Non-DSA+				Comparison	HLA Ab-Negative			
	Unblinded BLC		Blinded SC			Unblinded BLC		Blinded SC			Unblinded BLC		Blinded SC	
	*n*_*1*_	Mean (SD)	*n*_*2*_	Mean (SD)		*n*_*3*_	Mean (SD)	*n*_*4*_	Mean (SD)		*n*_*5*_	Mean (SD)	*n*_*6*_	Mean (SD)
**MARS Tac**														
T0	47	4.87 (0.18)	39	4.76 (0.64)		234	4.88 (0.15)	222	4.88 (0.22)		258	4.89 (0.21)	285	4.88 (0.17)
T12	28	4.89 (0.16)	16	4.88 (0.14)	*P* = 0.53	125	4.86 (0.21)	100	4.89 (0.20)	*P* = 0.29	100	4.90 (0.14)	101	4.87 (0.16)
T24	46	4.88 (0.19)	26	4.86 (0.17)	*P* = 0.57	184	4.86 (0.22)	157	4.89 (0.13)	*P* = 0.46	195	4.88 (0.16)	203	4.87 (0.23)
**% adherent on Tac trough levels**														
T0	51	88%	41	91%		260	96%	252	97%		303	97%	321	96%
T12	39	100%	19	86%	*P* = *0*.02	151	94%	130	96%	*P* = *0*.79	129	95%	139	95%
T24	60	92%	48	96%	*P* = *0*.41	270	95%	237	97%	*P* = *0*.17	280	95%	306	97%
**% adherent to Tac on composite adherence measure**														
T0	37	84%	28	78%		187	85%	185	88%		215	88%	225	85%
T12	23	82%	14	88%	*P* = *0*.64	94	79%	82	86%	*p* = *0*.21	81	87%	76	79%
T24	35	81%	20	80%	*P* = *0*.89	142	82%	135	91%	*P* = *0*.02	161	86%	160	84%
**MARS MMF**														
T0	40	4.89 (0.32)	39	4.76 (0.65)		212	4.89 (0.19)	190	4.88 (0.23)		259	4.90 (0.16)	255	4.88 (0.19)
T12	26	4.94 (0.11)	25	4.79 (0.32)	*P* = 0.03	114	4.86 (0.24)	94	4.86 (0.28)	*P* = 0.96	94	4.91 (0.13)	103	4.88 (0.17)
T24	44	4.85 (0.26)	30	4.87 (0.13)	*P* = 0.25	167	4.89 (0.16)	143	4.87 (0.16)	*P* = 0.13	186	4.89 (0.13)	190	4.89 (0.17)
**MARS Prednisolone**														
T0	32	4.86 (0.36)	28	4.80 (0.28)		178	4.90 (0.16)	151	4.91 (0.14)		187	4.86 (0.36)	209	4.88 (0.22)
T12	26	4.83 (0.40)	20	4.72 (0.34)	*P* = 0.04	97	4.87 (0.27)	68	4.93 (0.14)	*P* = 0.16	83	4.91 (0.14)	92	4.86 (0.25)
T24	44	4.86 (0.26)	25	4.83 (0.18)	*P* = 0.06	144	4.90 (0.20)	113	4.90 (0.13)	*P* = 0.51	138	4.90 (0.14)	154	4.87 (0.27)
**Concern about the risk of transplant failure**														
T0	73	7.27 (2.67)	67	6.75 (3.18)		338	7.38 (2.88)	306	7.30 (2.87)		380	8.0 (2.92)	411	8.01 (3.0)
T12	34	6.88 (2.80)	34	6.91 (2.66)	*P* = 0.98	148	6.91 (3.06)	127	7.25 (2.87)	*P* = 0.42	139	7.71 (2.87)	146	8.13 (2.76)
T24	62	6.97 (2.92)	42	6.64 (3.14)	*P* = 0.67	224	7.20 (2.69)	218	6.83 (2.94)	*P* = 0.24	264	7.78 (2.89)	285	7.72 (3.01)

T0 = Baseline; T12 = 12 months; T24 = 24 months; BLC = Biomarker led care; SC = Standard Care; MARS = Medication Adherence Rating Scale; Tac = tacrolimus; MMF = mycophenolate mofetil. Comparisons were made using Mann–Whitney U or Chi Squared tests.

## Discussion

OuTSMART is the first randomized double-blinded study in transplantation to test a stratified medicine approach to post-transplant care, based on HLA Ab status, and using graft failure as the primary endpoint. The results confirm the prognostic value of monitoring DSA, but find no evidence to support our hypothesis that intervening with optimised oral immunosuppression can prevent graft failure, with little separation in the Kaplan–Meier curves by group and confirmatory 95% confidence intervals for hazard ratios that included the null value. Further, there were no definitive signals in favour of biomarker-led care from any of the secondary outcomes in the HLA Ab+ groups, although biopsy-proven rejection was significantly lower in the BLC arm. These data will impact significantly on how transplant centres around the world organise their post-transplant monitoring and should encourage a global effort to find novel approaches to prevent allograft survival in the face of DSA.

The validity of HLA Ab as a prognostic biomarker for kidney transplant failure was first demonstrated in retrospective case control studies showing a higher prevalence of Ab against donor HLA in failed compared to working transplants.^4^ Later prospective studies reported a higher graft failure rate in those with HLA Ab compared to patients without, including a large study by Lachmann et al.^5^ on which OuTSMART was powered.

The development of HLA Ab conveys an increased risk of developing rejection, slowly progressive transplant dysfunction and graft failure^6^^,^^7^ and to date, no treatment strategies have been shown to be effective at preventing any of these outcomes. Multiple trials (reporting since OuTSMART started) of agents targeting B cells with rituximab (±IVIg)^10^^,^^22^ or plasma cells with bortezomib have failed to show any impact.^23^ Although agents targeting IL-6 or IL-6 receptor have shown early promise in early phase studies,^24^ larger studies assessing their impact are awaited. Other innovative treatments are at earlier stages of assessment.^25^ It is in this landscape that OuTSMART was originally conceived.

Our hypothesis was that targeting the cells of the immune system rather than the HLA Ab might prevent graft failure. There were three strands to this. First, activated T cells are required for development of HLA Ab.^8^^,^^26^, ^27^, ^28^ Second, immunosuppression reduction, including from non-adherence, associates with DSA and graft dysfunction.^8^^,^^29^ Finally, optimised oral immunosuppression can both prevent graft dysfunction,^12^, ^13^, ^14^ and slow the progression of deteriorating function in those with established immune-mediated dysfunction.^9^, ^10^, ^11^

In keeping with previous work, OuTSMART showed that 15–20% of grafts in DSA+ patients failed within the period of follow-up (after DSA detection) compared with 7% in the population who stayed consistently HLA Ab-negative. This is in line with observations from the Collaborative Transplant Survey (CTS Newsletter 2:2020 1st May), but is much lower than expected based on Lachmann et al.^5^ Lachmann et al. also reported a survival disadvantage associated with non-DSA,^5^ but in OuTSMART, those with non-DSA had a similar time to graft failure as patients without HLA Ab (Fig. 2E). A potential explanation for both differences is different population demographics, most prominently baseline maintenance immunosuppression. For example, the proportion of patients in OuTSMART taking either tacrolimus (73%) or MMF (67%) was double that in Lachmann's cohort (35% and 33%), reflecting shifts in practice over the last twenty years.

We screened more than 5000 patients, representing approximately half of the prevalent transplant population in the 13 UK centres in 2013 for inclusion in this trial. Although 62% of these were not included, most either failed to provide consent (1400) or didn't meet eligibility criteria (1867), which were designed to maximise safety and minimise ambiguity of data interpretation, so we are confident that the generalisability of our findings is not invalidated. Several factors in the design of OuTSMART deserve further explanation. First, patients known to be DSA+ (but XM-) at the time of transplantation accounted for ∼23% of recruits. Second, the majority of HLA Ab+ patients were DSA+ (∼66%) or non-DSA+ (∼68%) at the point of randomisation, and although most of these were de novo Ab (i.e. had developed post-transplantation), only a relative minority developed de novo Ab during our re-screening process. Both these were practical compromises, as recruiting sufficient numbers of HLA Ab-negative patients to collect enough DSA+ patients from re-screening alone was not feasible. Since patients with DSA that persist >12 months post-transplantation are at high risk of chronic rejection and graft failure,^30^^,^^31^ and at least one study has shown a similar prognostic significance for persistent non-DSA,^31^ both these decisions do not compromise the validity of the design. Third, we changed the primary endpoint during the study from graft failure rate over three years, to time to graft failure with minimum follow-up of 43 months. This was because the prevalence and incidence rates of DSA were lower than anticipated with consequent implications for the number of patients needed.^15^^,^^16^ This change preserved the power of the trial, without affecting the protocol or general modelling strategy. Although the minimum follow-up period was shortened due to the unplanned COVID-19 pandemic, our sensitivity analyses suggested this did not impact on our conclusions. Fourth, in the original design, development of HLA Ab triggered a transplant biopsy to correlate with graft pathology even in the absence of graft dysfunction. This design aspect was removed after a Patient Public Involvement session at which patients raised serious concerns. However, most clinicians would now want to perform a transplant biopsy in patients developing a DSA before deciding on future management. Fifth, after allocation into HLA Ab+ groups, no further screening was done until the final visit, at which point we were able to re-test 70–80%, revealing that only 50% remained DSA+. Whilst we are confident that our testing regimen, which involved a screening test followed by single antigen testing was identifying genuine DSA, these data might indicate heterogeneity within the DSA+ groups not accounted for in our design. However, a formal analysis of interaction between DSA persistence and our primary endpoint indicated non-significant differences on the hazard ratios.

Finally, we designed the trial as a ‘real world’ effectiveness study, such that optimisation was tailored to individual patients, according to compliance, tolerance and achievement of target tacrolimus levels. This aspect was regarded as highly important by patients and PIs, but resulted in relatively modest changes in immunosuppression in the BLC group, such that all BLC and SC groups had average tacrolimus levels within our target range, only 50% of the unblinded DSA+ group received the ‘steroid boost’ and many in the blinded groups were on immunosuppression that resembled our optimised regimen. Although tailoring to individual patient needs was a practical compromise, it is potentially a major limitation of this study and might have stopped us from revealing smaller benefits of our intervention.

That said, more than 95% of the unblinded Ab+ group had the intervention interview, the proportions at the end of the trial on tacrolimus, MMF and prednisolone in the BLC HLA Ab+ groups showed statistically significant increases compared to the proportions in the blinded HLA Ab+ groups, and the proportion taking all three IMPS increased significantly from 23% to 48% in unblinded DSA+ but stayed constant (∼22%) in the blinded DSA+ group. In addition, at the end of the trial, the BLC DSA+ group had the highest average tacrolimus levels and were on the highest average dose of MMF. Moreover, our formal assessments of adherence revealed evidence of significant differences for each of the IMPS in the BLC groups, at least at 12 months after the intervention. All these indicate measurable and significant differences related to our intervention, and are a likely explanation for why the frequency of biopsy-proven rejection was reduced in the BLC arm. Despite all this evidence that our intervention ‘worked’ we still failed to see an impact on time to graft failure.

There are a few analytical limitations. First, we have made certain assumptions as to missing data, the primary analysis assumes a missing at random mechanism (with data censored at loss to follow up or death). The effect of the intercurrent event of death was assessed with a sensitivity analysis (which showed similar results). Other than death, the percentage lost to follow up was very low (4%) and we consider that if the missingness mechanism were “missing not at random”, the impact on the results would be quite small. Secondary analyses also assumed a missing at random mechanism. Measures of the secondary outcomes are additionally affected by graft failure and death as intercurrent events, for which the data will be subsequently missing. We haven't directly assessed the impact of these intercurrent events for the secondary outcomes and so these treatment estimates should be considered as the treatment effect in the absence of death or graft failure (we think this is reasonable given the lack of evidence of an effect of the intervention on death or graft failure). Second, we have not adjusted reported p-values for multiplicity as we have defined a clear single primary outcome, and consequently the results on the secondary outcomes should be considered subsidiary and exploratory rather than confirmatory.

In conclusion, in this large, UK multicentre trial we have found no evidence that regular screening for HLA Ab in patients beyond 1-year post-transplantation, followed by tailored optimisation of immunosuppression impacts on graft failure. We conclude that, whilst screening for DSA has clear prognostic value and appears to reduce the incidence of rejection, we need novel strategies to intervene in this group to prevent graft failure before widespread routine screening should be adopted.

## Contributors

DS, RT-T, JP: trial statisticians had full access to all of the data in the study and take responsibility for the integrity of the data and the accuracy of the data analysis. LG Trial Manager. OS, BC, DB, JW, MB: HLA lab PIs. GD recruitment. RH, MP, RT, RB, RB, KM, JS, MP, SS, KYS, BW, AA, WA, JH; Site PIs and deputies. NB, AK, ZM, RH; health beliefs and compliance team; PM Health economist; EG, JK, CM; King's CTU, Database/Trial design. AD: CI, Trial design, Grant Holder, corresponding author.

## Data sharing statement

The data that support the findings of this study are available from the corresponding author upon reasonable request.

## Declaration of interests

DB declares consulting fees and speaker honoraria from Hansa Biopharma.

RT declares membership of ESOT Education Committee (2018–2021) (expenses reimbursed).

PM declares research funding from NIHR.

AD declares research funding from the 10.13039/501100007155Medical Research Council, consulting fees (paid to KCL) from Hansa Biopharma, Verici Diagnostics, UCB Pharma and Quell Therapeutics, Membership of the Herperis Faculty 2019, 2021 and 2022 (expenses reimbursed), Membership of the UK Organ donation and transplantation research network executive since 2020 (unpaid), Membership of the EME funding Committee (2014–2019) and the EME funding committee subgroup (2018–2019) (both unpaid).

The remaining authors declare no conflicts of interest.

## Appendix A Supplementary data

Caption for the supplementary material
