# Supplementary material for: Optimized immunosuppression to prevent graft failure in renal transplant recipients with HLA antibodies (OuTSMART): a randomised controlled trial
Source: eClinicalMedicine. 2023 Jan 12;56:101819. doi: 10.1016/j.eclinm.2022.101819 (PMC9852275; doi:10.1016/j.eclinm.2022.101819)
Supplement: Caption for the supplementary material [file mmc1.docx]

Caption for the supplementary material

The supplementary file contains additional detail on methodology and results. It includes a copy of the final protocol, summary of changes to the protocol, statistical analysis plan, summary of changes to the statistical analysis plan, and the SOP for how centres measured the HLA antibodies.
