# Supplementary material for: Optimized immunosuppression to prevent graft failure in renal transplant recipients with HLA antibodies (OuTSMART): a randomised controlled trial
Source: eClinicalMedicine. 2023 Jan 12;56:101819. doi: 10.1016/j.eclinm.2022.101819 (PMC9852275; doi:10.1016/j.eclinm.2022.101819)
Supplement: Supplementary material [file mmc2.docx]

# **Supplementary File**

## Table of Contents

**Supplementary File** 1

Table of Contents 1

List of Investigators and UK Recruiting Centres 5

Methods. 5

Anti-HLA Ab determination. 5

Expansion of statistical methodology 5

Primary outcome assumptions and sensitivity analyses 5

Adherence to drug therapy and perceptions of risk to the health of the transplant 7

Changes to the analysis from the SAP following discussion of the results and peer review comments 8

Results: 8

Protocol violations/Randomisation errors 8

Adverse events and changes in DSA 9

Proportional hazards assumption for primary outcome 10

References 10

Supplementary Figures 11

Supplementary Figure 1: OuTSMART trial design 11

Supplementary Figure 2: Diagram to help explain how endpoints were assessed 12

Supplementary Figure 3: Adverse events recorded in the HLA Ab+ groups 13

Supplementary Tables 14

Supplementary Table 1 14

Supplementary Table 2 15

Supplementary Table 3: 16

Supplementary Table 4: 17

Supplementary table 5: 18

Supplementary table 6: 19

Supplementary Table 7: 20

Supplementary Table 8: 21

**PROTOCOL TITLE:** 22

**Trial Identifiers** 22

**Sponsor** 22

**Chief Investigator** 22

**Name and address of Co-Investigator(s), Statistician, Laboratories etc** 22

**Study Synopsis** 24

**1. Background & Rationale** 30

**2. Trial Objectives and Design** 35

**3 Trial Medication** 41

**4. Selection and Withdrawal of Subjects** 41

**5. Trial Procedures** 44

**6 Assessment of Efficacy** 46

**7. Assessment of Safety** 48

**8. Statistics** 50

**9. Trial Steering Committee** 53

**10. Data Monitoring Committee** 53

**11. Direct Access to Source Data and Documents** 53

**12. Ethics & Regulatory Approvals** 54

**13. Quality Assurance** 54

**14. Data Handling, Publication Policy and Finance** 54

**15. Data Handling** 54

**16. Publication Policy** 54

**17. Insurance / Indemnity** 55

**18. Financial Aspects** 55

**19. Signatures** 55

**Appendix 1** 56

**Summary of changes to protocol approved by the ethics committee** 63

*Version 2 07/11/12:* 63

*Version 3 29/1/2013* 63

*Version 4 13/5/2013* 63

*Version 5 9/7/2013* 63

*Version 6 6/12/2013* 63

*Version 7 7/4/2014* 64

*Version 8 1/7/2014* 64

Version 9 15/10/2014 65

*Version 10 11/08/2015* 65

*Version 11 26/11/2015* 65

*Version 12 1/12/16* 65

*Version 13 21/11/18* 65

*Version14 08/07/2020* 66

A randomised controlled trial of a combined screening /treatment programme to prevent premature failure of renal transplants due to chronic rejection in patients with HLA antibodies (OuTSMART trial) 67

Statistical Analysis Plan 67

Version 2.4 67

Version 2.4 started: 09/02/2021 67

**Purpose and Scope of Statistical Analysis Strategy** 69

Contents 70

A) QUANTITATIVE ANALYSIS PLAN 71

Description of the trial 71

Protocol Publication: 71

Principal research objectives to be addressed 71

Trial design including blinding 72

Method of allocation of groups 74

Duration of the treatment period 75

Frequency and duration of follow-up 75

Data collection 76

Sample size estimation (including clinical significance) 79

Brief description of proposed analyses 82

1 Further changes in procedures in response to Covid-19 82

Data analysis plan – Data description 83

Descriptives by HLA status 83

Recruitment and representativeness of recruited patients 84

Baseline comparability of randomised groups 85

Adherence to allocated treatment and treatment fidelity 85

Loss to follow-up and other missing data 85

Adverse event reporting 85

Assessment of outcome measures (unblinding) 85

Descriptive statistics for outcome measures 85

Description of therapists/therapies 85

Data analysis plan – Inferential analysis 85

Main analysis of treatment differences 85

Exploratory analyses 88

Exploratory mediator and moderator analysis 88

Interim analysis 88

Software 88

B) ECONOMIC ANALYSIS AND ADHERENCE/RISK ASSESSMENT PLANS 88

C) SCHEDULE OF ASSESSMENTS AND MEASURES 91

D) Reference list 93

E) Amendments to version 1.0 93

SOP relating to HLA antibody determination 94

## List of Investigators and UK Recruiting Centres

The following PIs, based in 13 UK centres were: 1) Anthony Dorling (CI) and Rachel Hilton (PI) Guy’s Hospital, Guy’s and St Thomas’ NHS Foundation Trust; 2) Sapna Shah (PI) King’s College Hospital, King’s College Hospital NHS Foundation Trust; 3) Richard Baker (PI) St James’s University Hospital, Leeds Teaching Hospitals NHS Trust; 4) Michael Picton (PI) Manchester Royal Infirmary, Manchester University NHS Foundation Trust; 5) Raj Thuraisingham (PI) Royal London Hospital, Bart’s Health NHS Trust; 6) Richard Borrows (PI) Queen Elizabeth Hospital, University Hospitals Birmingham NHS Foundation Trust; 7) Keith McCullough (PI) York Hospital, York and Scarborough Teaching Hospitals NHS Foundation Trust; 8) Waqar Ayub (PI) University Hospital Coventry, University Hospitals Coventry and Warwickshire NHS Trust; 9) Aimun Ahmed (PI) Royal Preston Hospital, Lancashire Teaching Hospitals NHS Foundation Trust; 10) Janet Hegarty (PI) Salford Royal Hospital, Northern Care Alliance NHS Foundation Trust; 11) John Stoves (PI) Bradford Royal Infirmary, Bradford Teaching Hospitals NHS Foundation Trust; 12) Kin Yee Shiu and Stephen B Walsh (PIs) Royal Free Hospital, Royal Free NHS Foundation Trust; 13) Mysore Phanish (PI) St Helier Hospital, Epsom and St Helier University Hospitals NHS Foundation Trust.

## Methods.

### Anti-HLA Ab determination.

Serum prepared from 10 mL of blood was used in LABScreen tests (One Lambda, Canoga Park, CA through VH Bio, Gatehead UK), analysed on Luminex equipment (Luminex Corp, Austin, Texas) in five sites (Guy’s, Birmingham, Manchester, Leeds and Royal London). All worked to the same standard operating procedure, agreed pre-trial (refer to supplementary appendix). Serum was first analysed using mixed HLA class I and class II Ab screening beads, with a positive or negative result assigned based on batch-specific cut offs designated using validated protocols at the Guys site. Serum with a positive result on mixed bead screening was analysed using single antigen coated class I or class II beads, with a positive defined as mean fluorescence intensity (MFI) of binding ≥2,000. Laboratory staff then compared assigned DSA/non-DSA status depending on whether HLA Ab were directed against a mismatched donor HLA antigen. No retrospective HLA typing was performed. Instead, tissue typing staff inferred the presence of specific donor recipient mismatches when alleles were in strong linkage disequilibrium (see table 3). HLA Ab in which it was difficult to label as DSA + or non-DSA + (for instance because of insufficient data on donor mismatches), were categorised as non-DSA+. Samples with a positive reaction on screening but lacking reactivity with the single antigen beads were considered negative.

### Expansion of statistical methodology

### Primary outcome assumptions and sensitivity analyses

The following treatment effect contrasts for the primary and secondary outcomes were estimated:

1a. DSA+ BLC versus DSA+ SC participants (both at randomisation and re-screening)

1b. non-DSA+ BLC versus non-DSA+ SC participants (both at randomisation and re-screening)

2. All randomised BLC versus SC participants.

For the primary outcome, contrasts 1a and 1b were tested for superiority and contrast 2 was tested for non-inferiority, with non-inferiority concluded if the upper bound of the 95% confidence interval for the hazard ratio was less than 1.4. For all secondary outcomes, all contrasts were tested for superiority.

Different comparisons/outcomes used different observation periods. This is outlined in suppl figure 2 below.

The purpose of these different observation periods for the different comparisons, is that the within DSA+ and within non-DSA+ comparisons aim to estimate the treatment effect of unblinding + optimisation in HLA+ve participants and so include participants at risk from the time they were found to be HLA+ve. The overall unblinded (BLC) vs blinded (SC) comparison aims to estimate the overall effect of the blinding strategy, and so participants time at risk is the time of blinding/unblinding to the HLA result (which is randomisation for all participants).

For the DSA+ and within non-DSA+ comparisons, time at risk started at randomisation for those HLA positive at randomisation and at time of re-screening for those HLA Negative participants who became HLA positive later at re-screening rounds. For the primary outcome, patients follow up time was used up until the pre-COVID-19 collection period. For the overall comparison, time at risk started at randomisation for all participants up until the pre-COVID-19 collection period. This was also true for the secondary outcome of death.

For the other secondary outcomes, these were only collected in the intensive data collection period which was from randomisation to 32 months post-randomisation, or in the case of participants who became HLA positive at re-screening rounds, 32 months post-re-screening. Therefore for these secondary outcomes, for the within DSA+ and within non_DSA+ comparisons, time at risk starts at randomisation for those HLA positive at randomisation and at time of re-screening for those HLA Negative participants who became HLA positive later and ends 32 months later. However, for the overall unblinded (BLC) vs blinded (SC) comparison for these other secondary outcomes, time at risk starts at randomisation for all participants and ends 32 months post-randomisation (ignoring any additional follow up for re-screen HLA positive participants).

For the primary outcome, the proportional hazards assumption was checked by testing for an interaction between treatment and time or more precisely, testing for a non-zero slope in a generalized linear regression of the scaled Schoenfeld residuals on functions of time which is equivalent to testing the interaction). Log-log survival plots were also examined, overall and across strata to assess this assumption.

Several sensitivity analyses were also carried out for the primary outcome These used the same covariates/modelling strategy as the primary analysis unless stated:

1. Excluding site as a covariate: There were a large number of sites, and this was a stratification factor adjusted for the model. However, there were low numbers of participants recruited for some sites such that some estimates for the site covariate was not estimated in the model. An analysis excluding site was carried out to ensure this was not causing instability in treatment effect estimates.
2. A competing risks analysis using competing risk regression, according to the method of Fine and Gray (1999), was carried out to examine sensitivity of the results to the competing risk of death. The sub-hazard ratio for graft failure was estimated.
3. For COVID-19 data: An analysis was carried out using additional follow up data up until November 30th, 2020, which we called the post-COVID-19 timepoint as these participants’ outcomes may have been affected by the COVID-19 pandemic. The analysis was otherwise exactly the same.
4. Using the primary model for the HLA Ab Non-DSA group but restricting it to those participants who were assessed as definite non-DSA (as opposed to Non-DSA in the absence of any conclusive evidence of DSA).
5. A sensitivity/per protocol analysis restricting those in the HLA Ab+ DSA and HLA Ab+ non-DSA groups to those who received the full optimisation protocol (taking MMF, Tacrolimus and Prednisolone at the visit following the optimisation interview).

For the secondary outcome of death an additional sensitivity analysis restricting the follow up time to the first 32 months was carried out (as the original protocol implied that all secondary outcomes will be carried out on the 32 months intensive follow up period only).

The secondary outcomes of biopsy proven rejection, infection, malignancy, and diabetes de novo were all analysed using logistic regression, with the outcome as to whether the participant experienced the event (at least once) over the intensive 32 month follow up period (from randomisation (for overall comparison) or from re-screening (for DSA+ and non-DSA+) as appropriate). Site was not included as a covariate in these models as small numbers recruited in some sites would lead to perfect prediction and observations being dropped. Baseline immunosuppression was included as a covariate as per the primary outcome model, except where this also led to perfect prediction which occurred for the biopsy proven rejection outcome in the non-DSA+ comparison and so was removed. All participants were included if they had at least one observation post-randomisation (or post-rescreening).

The outcome of proteinuria at month 32 was analysed using a logistic (longitudinal) mixed model, with all observations included between randomisation (or re-screening as appropriate) and month 32 at 4 monthly intervals, although most participants only had data at 8 monthly intervals as frequency of follow up was changed to 8 monthly in Protocol V10 11/08/2015). Trial arm, timepoint, an interaction between timepoint and trial arm and stratification factors were included as covariates. A random intercept was included for participant. Treatment effects at month 32 were estimated using post-estimation commands. All participants were included if they had at least one observation post-randomisation (or post-rescreening).

The outcome of eGFR was analysed using a linear (longitudinal) mixed model, with timepoints as per the proteinuria model. Trial arm, timepoint, an interaction between timepoint and trial arm, baseline eGFR and the stratification factors were included as covariates. A random intercept was included for participant. Treatment effects at month 32 were estimated using post-estimation commands. All participants were included if they had at least one observation post-randomisation or post-rescreening (and so estimates are unbiased under a missing at random assumption as the model uses maximum likelihood). Residuals were examined to assess the normality of residuals assumption.

### Adherence to drug therapy and perceptions of risk to the health of the transplant

Health surveys, consisting of validated psychological measures adapted for this specific health context, were performed at baseline and 12 and 24 months post screening for HLA Ab+. The Medication Adherence report scale (MARS) questionnaire consisted of measuring six items on a five-point Likert scale with higher scores representing greater adherence. Most items assessed intentional medication non-adherence; one item measured unintentional non-adherence. The measure was completed for each concomitant medication that patients were receiving. MARS correlates well with relatively objective measures of adherence in a range of illness contexts, including electronic measures of inhaled corticosteroids for asthma and blood pressure control for hypertension^1,2^. It has also been shown to have good levels of internal consistency, test-retest reliability and construct validity ^2^.  For tacrolimus, 12 hour trough levels were also compared against the target trough levels (4-8ng/ml) intended on the trial. A composite adherence scale based on combining MARS scores with trough levels was developed. Concern about risk transplant failure was measured using the Brief Illness Perceptions Questionnaire (BIPQ) ^3^.

Analysis was performed separately to the main trial data by the team at UCL. Analyses were based on imputed data: where values were missing for a given survey item, the mean score for that item across all participants was used, providing that a given case had at least 80% complete data for other items on that scale. Mann Whitney U or Chi Squared tests were used to compare mean scores or percentages across patients in the BLC DSA+ compared to SC DSA+ groups, and BLC non DSA compared to SC non DSA groups.

### Changes to the analysis from the SAP following discussion of the results and peer review comments

The following changes were made to the analysis following discussion/review of the results and peer review comments and are not covered in the final Statistical Analysis Plan (V2.4 09/02/2021)

1. A post-hoc exploratory sensitivity analysis for the primary outcome was carried out (for each of the 3 comparisons) using only BLC participants taking all 3 IMPs and with Tacrolimus trough levels of between 6 and 8 compared to SC participants (and reported in Supplementary Table 2)
2. A post-hoc exploratory sensitivity analysis for the primary outcome was carried out (for each of the 3 comparisons) further adjusting for time of transplant and sex as covariates given chance imbalances between arms for these variables. Time to transplant appeared skewed (even after log transforming) and so was included categorised by quartiles.
3. McNemar tests were carried out comparing whether numbers on immunosuppression medications for BLC HLA+ participants (both DSA and non-DSA) changed from pre-optimisation to the last visit. This was to try to demonstrate that the optimisation intervention did change these participants immunosuppression medications as intended. This is reported in footnote for Table 4 in the main text.
4. A post-hoc analysis of the interaction between persisting DSA and time to graft failure in the main analysis was added to test whether those with persisting DSA and those without had different treatment effects. Results are described below in supplementary in the “Adverse events and changes in DSA” section.
5. The definition of what was classified as a biopsy proven rejection was not strictly defined in the SAP or the protocol. This was erroneously taken to be only those participants who showed rejection on the primary pathology for renal biopsies originally. Biopsy proven rejection is a secondary outcome. This became clear when responding to peer review and the Chief Investigator clarified that rejection on secondary pathology should also have been defined as biopsy proven rejection. This analysis was therefore amended to include these few additional events and the results changed slightly.
6. Where secondary outcomes reported odds ratios in the analysis, we have also estimated and reported adjusted risk differences in Supplementary table 8 to aid interpretation. Adjusted risk differences were estimated from the respective logistic regression models using post-estimation commands in Stata (margins).

## Results:

### Protocol violations/Randomisation errors

Consented and eligible participants were randomised by unblinded lab staff (using the KCTU bespoke randomisation system) following completion of baseline and HLA screening. Baseline HLA Ab status was entered into the randomisation system, and trial arm (and HLA Ab status if unblinded) communicated to the research staff at the site by emails generated by the system. Changes in HLA Ab status at re-screening rounds (every 8 months for HLA Ab-negative participants still in the trial up until Month 32) were similarly entered into the randomisation system, and emails sent by the system to the principal investigator about the change in HLA Ab status for participants in the unblinded (BLC) arm.

There were two randomisation errors, both participants were randomised to the Blinded (SC) arm and were HLA Ab-negative at baseline. One participant had graft failure prior to randomisation and one participant was randomised but was found to have died shortly before randomisation. Randomisation was carried out by lab staff following HLA screening and in error, it was not communicated to the lab staff that these events had occurred prior to randomisation. These participants are excluded from all analyses.

Further, for the primary analysis, one re-screened randomised participant is not included in the DSA group as they were found to have graft failure prior to being re-screened and becoming HLA Ab+ DSA and so were not at risk for the purpose of this analysis. This participant is included in any group/sensitivity analyses where time at risk starts at randomisation for all.

There were several other errors in recording of HLA status in the randomisation system. As per the intention to treat principle, these were analysed in the original groups as recorded in the randomisation data and not in the corrected group (as the HLA status as per randomisation data was communicated to the PI if in the unblinded arm, and treatment strategy would have been based on this data). These errors were the following:

- One participant randomised to the BLC arm and entered as HLA Ab+ DSA at baseline in randomisation system was actually HLA Ab+ definite non-DSA at baseline according to the lab data.
- One participant randomised to the SC arm and entered as HLA Ab+ DSA at baseline was actually HLA Ab+ definite non-DSA at baseline according to the lab data.
- One participant in the BLC arm was moved from the HLA Ab-negative to the HLA Ab+ DSA group at re-screening (at month 16). However according to their lab data, their antibodies at that time indicated “unknown whether DSA” and should have been allocated to the non-DSA group as per the protocol.
- One participant randomised to the BLC arm and entered as DSA at baseline actually had antibodies at that time indicating “unknown whether DSA” and should have been randomised to the non-DSA group
- Two participants randomised to the SC arm who were HLA Ab-negative at baseline were re-screened according to lab data at Month 16 and became HLA Ab+ with unknown DSA. However, this was erroneously not entered into the randomisation system (and are considered HLA Ab-negative for the purpose of ITT analysis).

### Adverse events and changes in DSA

A total of 8189 AEs (670 SAES) were reported, and 1570 patients (77%) experienced at least one AE (supplementary table 6). Significant differences were observed for 5 outcomes/codes with HLA Ab+ participants in the BLC arm being more likely to experience cardiovascular, respiratory, gastrointestinal and GU/renal AEs than HLA Ab+ participants in the SC arm (supplementary figure 2). These comparisons are not adjusted for multiple testing however, and any adverse events of concern are covered by existing secondary outcomes.

By the end of intensive follow up, fewer than 2% of the HLA Ab-negative groups became Ab+, more than 50% of the DSA+ participants became HLA Ab-negative, 16-23% lost their DSA but retained non-DSA HLA Ab, and 60-70% of the non-DSA+ participants became Ab-negative. 5.1% of the SC non-DSA+ recruits had developed DSA or possible DSA, compared to 1.6% of the unblinded non-DSA+ participants (table 2). Within the blinded SC group, the same proportion (2/21 [9.5%]) of those with persisting DSA had graft failure as those who became DSA-negative (4/48 [8.3%]). Although in the BLC group, only 2/50 (4%) of the recruits who lost DSA suffered graft failure, compared to 6/28 (21.4%) with persisting DSA, a formal post-hoc analysis of the interaction between persisting DSA and time to graft failure in the main analysis revealed non-significant differences (p=0.316) in revised HRs. Further analysis of within group interactions were not undertaken as numbers were small.

Proportional hazards assumption for primary outcome

The proportional hazards assumption was assessed for the primary outcome by examining log-log plots and by testing whether the log-hazard ratio is constant over time (using *phtest* in Stata). Log time was used. There was no evidence for any of the three comparisons (witihin DSA+ comparison p=0.89 , within non-DSA comparison p=0.74, overall comparison p=0.58) that this assumption was violated.

## References

1. Cohen JL, Mann DM, Wisnivesky JP, et al. Assessing the validity of self-reported medication adherence among inner-city asthmatic adults: the Medication Adherence Report Scale for Asthma. *Ann Allergy Asthma Immunol* 2009; **103**(4): 325-31.

2. Chan AHY, Horne R, Hankins M, Chisari C. The Medication Adherence Report Scale: A measurement tool for eliciting patients' reports of nonadherence. *Br J Clin Pharmacol* 2020; **86**(7): 1281-8.

3. Broadbent E, Petrie KJ, Main J, Weinman J. The brief illness perception questionnaire. *J Psychosom Res* 2006; **60**(6): 631-7.

## Supplementary Figures

### Supplementary Figure 1: OuTSMART trial design


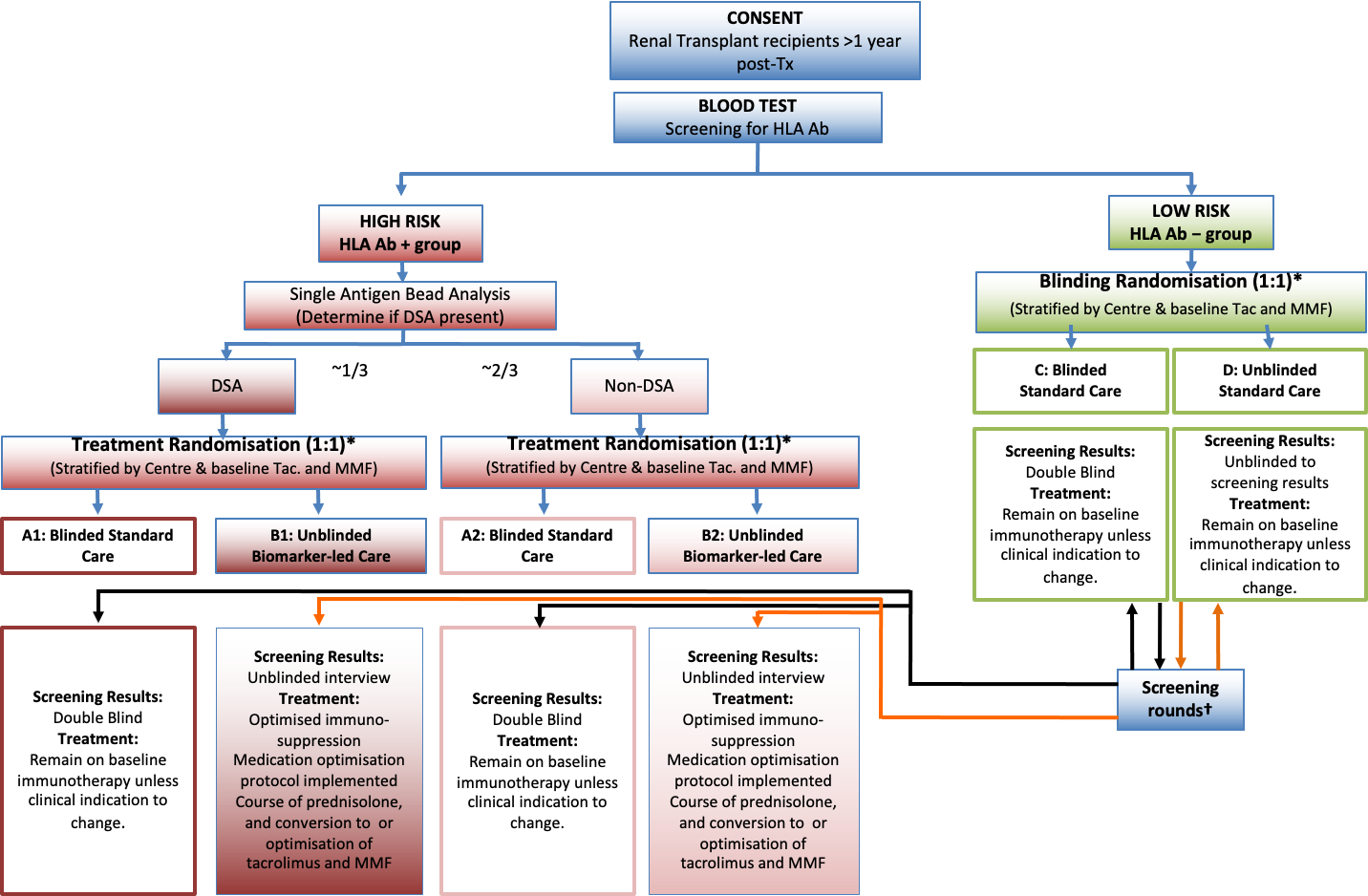


Legend to Supplementary Figure 1: *Randomisation was performed after results of a recruit’s first screening test result. Those with HLA Ab underwent no further screening as part of the trial but had a final test performed at the last formal visit.

**^†^**Those initially HLA Ab-negative underwent routine screening every 8 months. There was no second randomisation: If a recruit allocated to Blinded standard care (group C) became HLA Ab positive (black lines), they remained in Standard care group (group A1 or A2). If in unblinded group (D), they changed to unblinded biomarker-led care (group B1 or B2) (orange lines). For each patient moving this way, the trial clock was ‘reset’ to time zero at that point, and they underwent a further 32 months of follow-up. To maintain blinding in group C, the randomisation system was programmed at each screening round to choose a random group of HLA Ab-negative recruits to complete a further 32 months follow-up.

### Supplementary Figure 2: Diagram to help explain how endpoints were assessed

HLA Ab- at all tests

HLA Ab- at enrolment

DSA+month8

HLA Ab- at enrolment

Non-DSA+month16

1

2

3

Month 32

Month 64

Period of assessment of all other secondary EPs in HLA Ab+ BLC vs. HLA Ab+ SC groups

Period of assessment of overall graft failure in BLC vs. SC arms and death

Ignore graft failure for primary endpoint and selected outcome analyses of secondary endpoints in HLA Ab+ groups

Period of assessment of all other secondary EPs in overall BLC vs. SC comparison

Period of assessment of primary endpoints

Primary endpoints

Secondary endpoints

Legend to supplementary figure 2. Figure depicts 3 recruitment scenarios and explains how different outcomes were assessed, either in relation to recruitment/randomisation or, in the case of recruits turning from HLA Ab-negative to +, in relation to re-screen.

### Supplementary Figure 3: Adverse events recorded in the HLA Ab+ groups


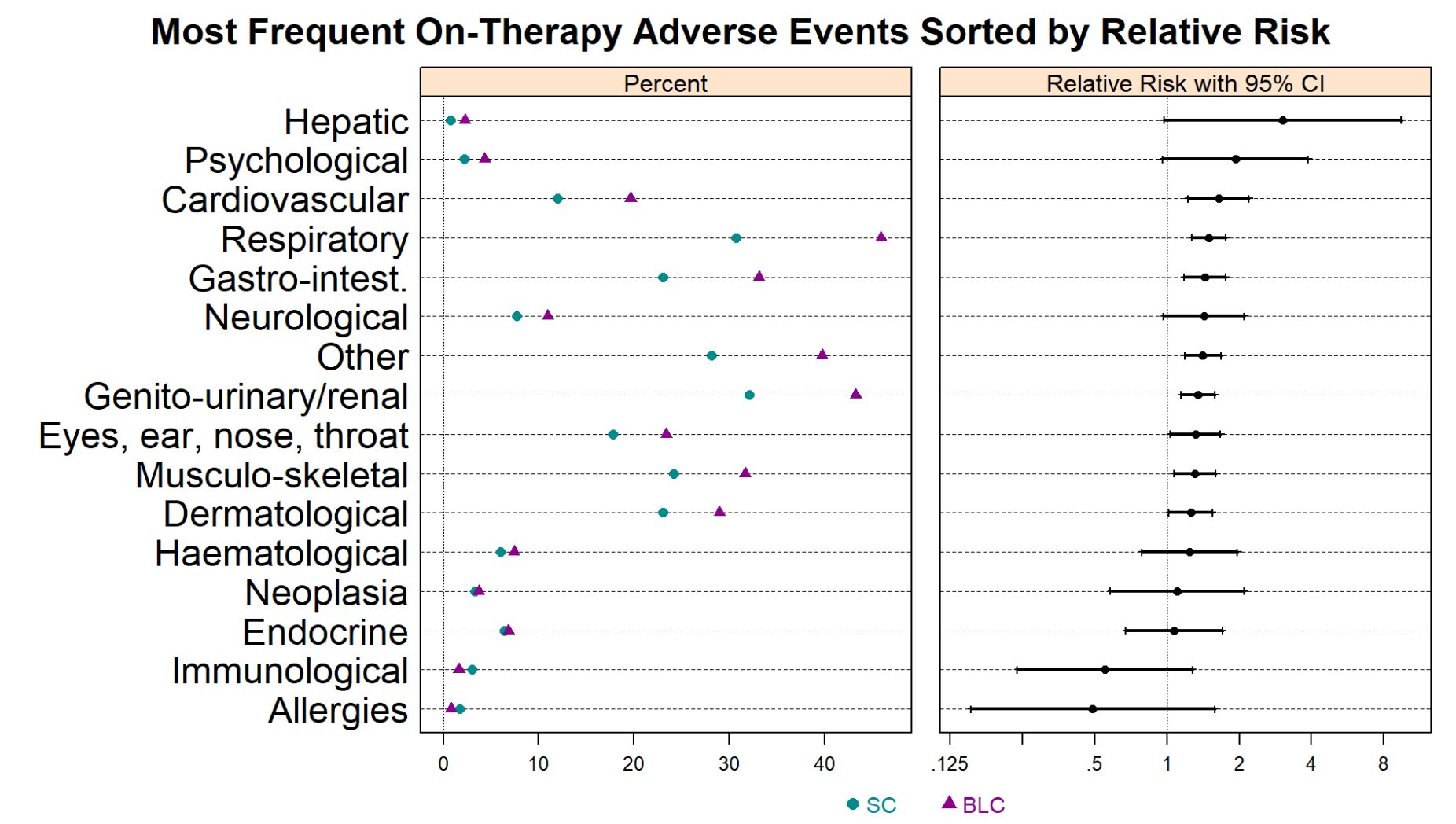


Legend to Supplementary Figure 3

Left panel compares the proportion of patients suffering adverse events grouped by body system in blinded (SC: blue circles) and unblinded (BLC: purple triangles) HLA Ab+ groups. Right panel shows relative risk (95% CI) of developing an adverse event in the BLC patients, ordered by size of relative risk.

## Supplementary Tables

### Supplementary Table 1

Prevalent population of UK transplant recipients being followed up at OuTSMART recruiting centres 2013-2016

|  | 2013 | 2014 | 2015 | 2016 |
| --- | --- | --- | --- | --- |
| Leeds | 890 | 916 | 954 | 976 |
| Royal London | 952 | 1,041 | 1,072 | 1,141 |
| Guy’s | 1,182 | 1,240 | 1,302 | 1,365 |
| Manchester | 1,259 | 1,218 | 1,305 | 1,398 |
| Birmingham | 1,163 | 1,231 | 1,291 | 1,411 |
| King’s College Hospital | 362 | 393 | 429 | 436 |
| York | 242 | 289 | 300 | 306 |
| Coventry | 471 | 504 | 518 | 531 |
| Preston | 487 | 548 | 591 | 605 |
| Salford | 411 | 470 | 483 | 513 |
| Bradford | 288 | 305 | 330 | 359 |
| Royal Free | 1,093 | 1,155 | 1,221 | 1,288 |
| St Helier | 604 | 636 | 652 | 685 |
| Total | 9,404 | 9,946 | 10,448 | 11,014 |
|  |  |  |  |  |
| All UK | 29,592 | 31,164 | 32,624 | 34,107 |

Source – UK Kidney Association Annual reports (<https://ukkidney.org/audit-research/annual-report/all>)

### Supplementary Table 2

Combinations of drugs taken by participants in the BLC groups pre-and post-optimization

| Which immunosuppression drugs (of Tac,Pred and MMF) is participant taking? | DSA+ BLC | | Non-DSA BLC | |
| --- | --- | --- | --- | --- |
|  | Baseline/post-screening | Immediately post-optimization | Baseline/post-screening | Immediately post-optimization |
| Not taking Pred, MMF or Tac | 7 (6.7%) | 3 (3.1%) | 20 (4.7%) | 9 (2.2%) |
| Pred only | 11 (10.5%) | 4 (4.1%) | 36 (8.4%) | 21 (5.2%) |
| MMF only | 5 (4.8%) | 1 (1.0%) | 16 (3.8%) | 8 (2.0%) |
| Tac only | 13 (12.4%) | 3 (3.1%) | 57 (13.4%) | 35 (8.7%) |
| Pred and MMF only | 15 (14.3%) | 7 (7.1%) | 42 (9.8%) | 21 (5.2%) |
| Tac and Pred only | 12 (11.4%) | 14 (14.3%) | 43 (10.1%) | 40 (9.9%) |
| Tac and MMF only | 18 (17.1%) | 13 (13.3%) | 107 (25.1%) | 92 (22.8%) |
| All three drugs | 24 (22.9%) | 53 (54.1%) | 106 (24.8%) | 178 (44.1%) |
| Total | 105 | 98 | 427 | 404 |

Percentages are column percentages.

### Supplementary Table 3:

Sensitivity analyses on the primary outcome

| **Group/comparison** | **Hazard Ratio** | **Lower 95% CI** | **Upper 95% CI** | **P-value** |
| --- | --- | --- | --- | --- |
| **Post-COVID analysis^1^** | | | | |
| DSA (N=197^2^) | 1.29 | 0.64 | 2.60 | 0.48 |
| Non-DSA (N=818) | 1.05 | 0.61 | 1.82 | 0.86 |
| All participants (N=2035^3^) | 1.03 | 0.74 | 1.42 | 0.88 |
| **Excluding site as a co-variate** | | | | |
| DSA (N=197) | 1.51 | 0.72 | 3.19 | 0.28 |
| Non-DSA (N=818) | 0.98 | 0.54 | 1.75 | 0.93 |
| All participants (N=2035) | 1.02 | 0.72 | 1.45 | 0.91 |
| **Competing risk of death** | | | | |
| DSA (N=197) | 1.53 | 0.70 | 3.35 | 0.29 |
| Non-DSA (N=818) | 0.96 | 0.53 | 1.74 | 0.90 |
| All participants (N=2035) | 1.01 | 0.71 | 1.43 | 0.96 |
| **Randomisation as time zero^4^** | | | | |
| DSA (N=198) | 1.35 | 0.64 | 2.86 | 0.43 |
| Non-DSA (N=818) | 0.96 | 0.53 | 1.72 | 0.88 |
| **Analysis of only those who underwent IS optimization (using only BLC participants taking all 3 IMPs)^5^** | | | | |
| DSA (N=145) | 1.17 | 0.44 | 3.14 | 0.75 |
| Non-DSA (N=569) | 0.96 | 0.44 | 2.10 | 0.91 |
| All participants (N=1238) | 1.21 | 0.71 | 2.09 | 0.48 |
| **Analysis of only those with definite non-DSA** | | | | |
| DSA (N=283) | 1.47 | 0.76 | 2.85 | 0.25 |
| Non-DSA (N=729) | 0.90 | 0.46 | 1.73 | 0.74 |
| **Post-hoc sensitivity with sex and time since transplant as additional covariates** | | | | |
| DSA (N=197) | 1.60 | 0.73 | 3.49 | 0.24 |
| Non-DSA (N=818) | 1.02 | 0.56 | 1.85 | 0.96 |
| All participants (N=2035) | 1.00 | 0.71 | 1.43 | 0.98 |
| **Post-hoc sensitivity using only BLC participants taking all 3 IMPs with tac levels 6-8^6^** | | | | |
| DSA (N=118) | 1.23 | 0.30 | 4.98 | 0.77 |
| Non-DSA (N=496) | 0.70 | 0.23 | 2.12 | 0.53 |
| All participants (N=1138) | 1.02 | 0.48 | 2.17 | 0.96 |

Sensitivity analyses performed on primary endpoint

Footnotes: ^1^Post-COVID (1^st^ wave) analysis included additional graft failure events that occurred between March 16^th^ 2020, and end November 2020 that included COVID outcomes. ^2^One HLA-Ab-negative participant in the blinded (SC) group who developed DSA on re-screening was not included in this analysis as the graft failed prior to re-screening, so they were not at risk for the purpose of this analysis. ^3^Although 2037 randomised, 2 patients in the HLA Ab-negative group were excluded from the analysis – see text and figure 2: ^4^Using randomisation as time zero for all (as opposed to time of re-screen for those initially HLA Ab-negative at time of randomisation)

^5^Comparing the 53 (54%) BLC DSA+ recruits and the 178 (44%) BLC non-DSA+ recruits who were on optimised onto all three IMPs, with all SC DSA+ and SC non-DSA+ recruits.

^6^Comparing the 26 (26.5%) BLC DSA+ recruits and the 105 (26%) BLC non-DSA+ recruits who were on optimised onto all three IMPS with tacrolimus levels at the higher end of the range we targeted, with all SC DSA+ and SC non-DSA+ recruits.

### Supplementary Table 4:

Histological diagnoses in DSA+ patients undergoing biopsy during the 32 months post-identification of DSA

| BANFF 09 Classification | Unblinded BLC DSA+ | Blinded SC DSA+ |
| --- | --- | --- |
| Category 1 Normal | 2 | 0 |
| Category 2 ABMR | 2 | 8 |
| C4d deposition only | 0 | 1 |
| subtype 1 | 0 | 0 |
| subtype 2 | 0 | 2 |
| subtype 3 | 1^1^ | 0 |
| chronic | 1 | 5^2^ |
| subtype NOS | 0 | 0 |
| Category 3 Borderline change | 2 | 3 |
| Category 4 TCMR | 3 | 2 |
| subtype IA | 1 | 2^2^ |
| subtype IB | 1 | 0 |
| subtype IIA | 0 | 0 |
| subtype IIB | 0 | 0 |
| subtype III | 1^1^ | 0 |
| subtype NOS | 0 | 0 |
| Category 5 IFTA without specific cause | 5 | 3 |
| Grade I | 2 | 0 |
| Grade II | 3^1^ | 3 |
| Grade III | 0 | 0 |
| Category 6 | 5* | 6† |
| Insufficient sample | 2 | 0 |
| Totals | 21 | 22 |

Definitions: ABMR antibody mediated rejection; TCMR T cell mediated rejection; IFTA interstitial fibrosis with tubular atrophy.

Table shows only the findings of biopsies performed after patients were allocated to either of the two 2 DSA+ groups. If patients were originally allocated to the HLA-Ab-negative groups, and had a biopsy prior to DSA development, these are not included in the above table.

4 DSA + patients in unblinded BLC group were diagnosed with rejection on biopsy. NB ^1^Is a single patient with mixed ABMR subtype 3 and TCMR subtype III (plus IFTA grade II) in same biopsy.

9 DSA+ patients in blinded group were diagnosed with rejection on biopsy. NB ^2^ is a single patient with mixed TCMR subtype IA and chronic ABMR in same biopsy.

Category 6 diagnoses recorded

*Recurrent IgA, chronic ischaemia, diabetic change, not recorded x2

† Chronic TIN, membranous nephropathy, acute tubular injury, not recorded x3,

Supplementary table 5:

Number (percentage) of participants who experienced infections by type in intensive follow up period

|  | DSA+ | | Non-DSA+ | | No HLA Ab | |  |
| --- | --- | --- | --- | --- | --- | --- | --- |
| Infections | Blinded (SC) | Unblinded (BLC) | Blinded (SC) | Unblinded (BLC) | Blinded (SC) | Unblinded (BLC) | Total |
| **All Infection types** | 48 (52%) | 65 (61%) | 225 (58%) | 260 (61%) | 276 (53%) | 241 (50%) | 1115 (55%) |
| **All Infection types (confirmed by culture or PCR)** | 21 (23%) | 32 (30%) | 95 (25%) | 109 (26%) | 115 (22%) | 107 (22%) | 479 (24%) |
| **Viral** | 24 (26%) | 30 (28%) | 105 (27%) | 134 (32%) | 120 (23%) | 95 (20%) | 512 (25%) |
| **Viral (confirmed)** | 4 (4.3%) | 5 (4.7%) | 19 (4.9%) | 22 (5.2%) | 25 (4.8%) | 22 (4.5%) | 97 (4.8%) |
| BK | 0 (0.0%) | 0 (0.0%) | 1 (0.3%) | 3 (0.7%) | 6 (1.1%) | 2 (0.4%) | 12 (0.6%) |
| CMV | 2 (2.2%) | 3 (2.8%) | 4 (1.0%) | 6 (1.4%) | 0 (0.0%) | 2 (0.4%) | 17 (0.8%) |
| EBV | 1 (1.1%) | 2 (1.9%) | 4 (1.0%) | 6 (1.4%) | 5 (1.0%) | 4 (0.8%) | 22 (1.1%) |
| Shingles | 1 (1.1%) | 1 (0.9%) | 1 (0.3%) | 1 (0.2%) | 5 (1.0%) | 3 (0.6%) | 12 (0.6%) |
| **Bacterial** | 34 (37%) | 46 (43%) | 157 (41%) | 183 (43%) | 197 (38%) | 173 (36%) | 790 (39%) |
| **Bacterial (confirmed by culture or PCR)** | 19 (21%) | 31 (29%) | 81 (21%) | 93 (22%) | 96 (18%) | 90 (19%) | 412 (20%) |
| UTI | 12 (13%) | 19 (18%) | 64 (17%) | 66 (16%) | 65 (12%) | 59 (12%) | 286 (14%) |
| Pneumonia | 0 (0.0%) | 4 (3.8%) | 7 (1.8%) | 6 (1.4%) | 12 (2.3%) | 10 (2.1%) | 39 (1.9%) |
| TB | 0 (0.0%) | 1 (0.9%) | 1 (0.3%) | 0 (0.0%) | 1 (0.2%) | 1 (0.2%) | 4 (0.2%) |
| **Fungal** | 4 (4.3%) | 6 (5.7%) | 23 (5.9%) | 15 (3.5%) | 18 (3.4%) | 17 (3.5%) | 83 (4.1%) |
| **Fungal (confirmed by culture or PCR)** | 0 (0.0%) | 3 (2.8%) | 4 (1.0%) | 6 (1.4%) | 5 (1.0%) | 4 (0.8%) | 22 (1.1%) |
| Pneumocystis jirovecii | 0 (0.0%) | 0 (0.0%) | 1 (0.3%) | 1 (0.2%) | 0 (0.0%) | 2 (0.4%) | 4 (0.2%) |

Supplementary table 6:

Percentage (number) of participants who experienced biopsy-proven malignancies by site of malignancy and group

|  | DSA+ | | Non-DSA+ | | No HLA Ab | |  |
| --- | --- | --- | --- | --- | --- | --- | --- |
| Site of malignancy | Blinded (SC) | Unblinded (BLC) | Blinded (SC) | Unblinded (BLC) | Blinded (SC) | Unblinded (BLC) | Total |
| Skin | 3 (3.3%) | 6 (5.7%) | 23 (5.9%) | 23 (5.4%) | 24 (4.6%) | 15 (3.1%) | 94 (4.7%) |
| Lymph node | 0 (0.0%) | 1 (0.9%) | 4 (1.0%) | 2 (0.5%) | 1 (0.2%) | 2 (0.4%) | 10 (0.5%) |
| Lung | 1 (1.1%) | 0 (0.0%) | 0 (0.0%) | 1 (0.2%) | 0 (0.0%) | 2 (0.4%) | 4 (0.2%) |
| Liver | 0 (0.0%) | 0 (0.0%) | 0 (0.0%) | 0 (0.0%) | 0 (0.0%) | 0 (0.0%) | 0 (0.0%) |
| Breast | 0 (0.0%) | 0 (0.0%) | 0 (0.0%) | 1 (0.2%) | 0 (0.0%) | 0 (0.0%) | 1 (0.1%) |
| Prostate | 0 (0.0%) | 0 (0.0%) | 3 (0.8%) | 0 (0.0%) | 3 (0.6%) | 0 (0.0%) | 6 (0.3%) |
| Stomach | 0 (0.0%) | 0 (0.0%) | 0 (0.0%) | 1 (0.2%) | 0 (0.0%) | 2 (0.4%) | 3 (0.1%) |
| Colon | 1 (1.1%) | 0 (0.0%) | 1 (0.2%) | 0 (0.0%) | 3 (0.6%) | 0 (0.0%) | 5 (0.3%) |
| Cervical/vaginal | 0 (0.0%) | 0 (0.0%) | 1 (0.3%) | 1 (0.2%) | 0 (0.0%) | 0 (0.0%) | 2 (0.1%) |
| Bladder | 0 (0.0%) | 0 (0.0%) | 1 (0.3%) | 1 (0.2%) | 0 (0.0%) | 0 (0.0%) | 2 (0.1%) |
| Blood | 0 (0.0%) | 1 (0.9%) | 0 (0.0%) | 1 (0.2%) | 0 (0.0%) | 0 (0.0%) | 2 (0.1%) |
| Kidney | 0 (0.0%) | 0 (0.0%) | 0 (0.0%) | 0 (0.0%) | 1 (0.2%) | 1 (0.2%) | 2 (0.1%) |
| Tongue /throat /larynx | 1 (1.1%) | 1 (0.9%) | 3 (0.8%) | 2 (0.5%) | 0 (0.0%) | 0 (0.0%) | 7 (0.4%) |
| Other | 0 (0.0%) | 0 (0.0%) | 0 (0.0%) | 2 (0.5%) | 3 (0.6%) | 2 (0.4%) | 7 (0.4%) |
| **Total^1^** | 6 (6.5%) | 10 (9.4%) | 35 (9.0%) | 38 (9.0%) | 36 (6.9%) | 25 (5.2%) | 150 (7.4%) |

^1^Total numbers do not add up to sum of columns exactly as for a couple of participants, separate biopsy proven malignancies were reported at different sites over the course of the trial

Percentages are percentages of participants who experienced that type/site of malignancy out of all randomised participants in that group.

### Supplementary Table 7:

Number (%) of participants who experienced an adverse event within each body system code and group

|  | DSA+ | | Non-DSA+ | | No HLA Ab | |  |
| --- | --- | --- | --- | --- | --- | --- | --- |
| Body system | Blinded (SC) | Unblinded (BLC) | Blinded (SC) | Unblinded (BLC) | Blinded (SC) | Unblinded (BLC) | Total |
| Allergies | 1 (1.1%) | 0 (0.0%) | 8 (2.0%) | 4 (0.9%) | 3 (0.6%) | 1 (0.2%) | 17 (0.8%) |
| Cardiovascular | 4 (4.3%) | 15 (14%) | 60 (15%) | 80 (19%) | 66 (13%) | 57 (12%) | 282 (14%) |
| Dermatological | 24 (26%) | 27 (26%) | 99 (25%) | 113 (27%) | 101 (19%) | 76 (15%) | 440 (22%) |
| Endocrine | 6 (6.5%) | 7 (6.6%) | 28 (7.2%) | 26 (6.1%) | 29 (5.5%) | 21 (4.2%) | 117 (5.7%) |
| Eyes, ear, nose, throat | 19 (21%) | 24 (23%) | 76 (19%) | 89 (21%) | 82 (16%) | 59 (12%) | 349 (17%) |
| Gastro-intestinal | 29 (32%) | 32 (30%) | 94 (24%) | 128 (30%) | 116 (22%) | 105 (21%) | 504 (25%) |
| Genito-urinary/renal | 30 (33%) | 46 (43%) | 141 (36%) | 163 (38%) | 163 (31%) | 130 (26%) | 673 (33%) |
| Haematological | 7 (7.6%) | 7 (6.6%) | 25 (6.4%) | 29 (6.8%) | 35 (6.7%) | 26 (5.3%) | 129 (6.3%) |
| Hepatic | 2 (2.2%) | 2 (1.9%) | 2 (0.5%) | 9 (2.1%) | 4 (0.8%) | 6 (1.2%) | 25 (1.2%) |
| Immunological | 3 (3.3%) | 1 (0.9%) | 13 (3.3%) | 7 (1.6%) | 9 (1.7%) | 4 (0.8%) | 37 (1.8%) |
| Musculo-skeletal | 26 (28%) | 32 (30%) | 103 (26%) | 121 (28%) | 134 (26%) | 106 (21%) | 522 (26%) |
| Neoplasia | 2 (2.2%) | 1 (0.9%) | 16 (4.1%) | 17 (4.0%) | 9 (1.7%) | 9 (1.8%) | 54 (2.7%) |
| Neurological | 12 (13%) | 10 (9.4%) | 29 (7.4%) | 43 (10%) | 31 (5.9%) | 30 (6.1%) | 155 (7.6%) |
| Psychological | 2 (2.2%) | 3 (2.8%) | 10 (2.6%) | 18 (4.2%) | 23 (4.4%) | 11 (2.2%) | 67 (3.3%) |
| Respiratory | 37 (40%) | 46 (43%) | 127 (33%) | 176 (41%) | 177 (34%) | 149 (30%) | 712 (35%) |
| Other | 34 (37%) | 35 (33%) | 116 (30%) | 157 (37%) | 150 (29%) | 118 (24%) | 610 (30%) |

Percentages use number of randomised recruits in each group as denominator

### Supplementary Table 8:

Adjusted risk differences for secondary outcomes

Negative risk differences represent a lower risk of the event in the unblinded BLC group compared to the blinded SC group (and positive risk differences a higher risk). P-values remain unchanged and are not presented here; see Table 5 in main paper for results.

| **Group/Comparison** | (Adjusted) Risk difference^1^ | **95% CI** |
| --- | --- | --- |
| **Biopsy Proven rejection** |  |  |
| DSA (N=198) | -5.7% | -12.4% to 0.9% |
| Non-DSA (N=818) | -0.9% | -2.6% to 0.9% |
| All participants (N=2035) | -1.4% | -2.6% to -0.2% |
| **Confirmed infection** |  |  |
| DSA (N=197) | 10.2% | -1.8% to 22.2% |
| Non-DSA (N=809) | 1.5% | -4.4% to 7.4% |
| All participants (N=2010) | 1.4% | -2.3% to 5.1% |
| **Malignancy** |  |  |
| DSA (N=198) | 0.5% | -6.1% to 7.0% |
| Non-DSA (N=810) | -0.6% | -4.3% to 3.2% |
| All participants (N=2015) | -0.5% | -2.6% to 1.6% |
| **Diabetes Mellitus** |  |  |
| DSA (N=198) | -0.1% | -3.8% to 3.7% |
| Non-DSA (N=818) | -1.7% | -4.1% to 0.7% |
| All (N=2015) | -0.6% | -1.7% to 0.6% |
| **Proteinuria** |  |  |
| DSA (N=184) | -3.2% | -9.1% to 2.7% |
| Non-DSA (N=788) | 1.3% | -1.8% to 4.4% |
| All participants (N=1972) | -0.7% | -2.6% to 1.1% |

**PROTOCOL TITLE:**

A randomized controlled clinical trial to determine if a combined screening /treatment programme can prevent premature failure of renal transplants due to chronic rejection in patients with HLA antibodies.

Short Title – ‘Optimized TacrolimuS and MMF for HLA Antibodies after Renal Transplantation:’ – “OuTSMART”

**Trial Identifiers**

EudraCT Number – 2012-004308-36

ISRCTN – 46157828

REC Number – 12/L0/1759

**Sponsor**

Name: Amy Holton, King’s College London and Guy’s and St Thomas’ NHS Foundation Trust.

Address: King’s Health Partners Clinical Trials Office, Guy’s Hospital, Great Maze Pond, London SE1 9RT

Telephone: 020 7188 5732

Fax: 020 7188 8330

Email: amy.holton@kcl.ac.uk

**Chief Investigator**

Name: Professor Anthony Dorling

Address: MRC Centre for Transplantation, King’s College London, Guy’s Hospital, Great Maze Pond, London, SE1 9RT

Telephone: 020 7188 5880

Fax: 020 7188 5660

Email: anthony.dorling@kcl.ac.uk

**Name and address of Co-Investigator(s), Statistician, Laboratories etc**

Name: Dr Rachel Hilton

Address: Dept of Nephrology and Transplantation, Guy’s Hospital, Great Maze Pond, London SE1 9RT

Telephone: 020 7188 5691

Fax: 020 7188 5646

Email: Rachel.Hilton@gstt.nhs.uk

Name: Mr Dominic Stringer

Address: Biostatistics Department, Institute of Psychiatry, Psychology and Neuroscience, Kings College London, London, SE5 8AF

Telephone: 020 7848 0323

Email: dominic.stringer@kcl.ac.uk

Name: Professor Janet Peacock

Address: King’s College London, Guy’s Hospital, London SE1 9RT

Telephone: 020 7848 6651

Fax:

Email: janet.peacock@kcl.ac.uk

Name: Dr Caroline Murphy

Address: Clinical Trials Unit, King’s College London, PO64, M2.06, Institute of Psychiatry

Telephone:020 7848 5273

Fax: 020 7848 5229

Email: caroline.murphy@kcl.ac.uk

Name: Dr Olivia Shaw

Address: Clinical Transplantation Laboratory, 3^rd^ Floor Borough Wing, Guy’s Hospital, GSTT Foundation Trust,

Telephone: 020 7188 1531

Fax: 020 7407 6370

Email: Olivia.Shaw@viapath.co.uk

Name: Dr Richard Baker

Address: Renal Unit, St James’s University Hospital, Beckett Street, Leeds, LS9 7TF

Telephone: 0113 2066869

Fax: 0113 2066216

Email: Richard.Baker@leedsth.nhs.uk

Name: Dr Brendan Clarke,

Address: Transplant Immunology, Level 09 Gledhow Wing, St James's University Hospital, Beckett Street, Leeds LS9 7TF

Telephone: (0113) 2064579

Fax:

Email: Brendan.Clarke@leedsth.nhs.uk

Name: Dr Raj Thuraisingham

Address: Renal Unit, The Royal London Hospital, London E1 1BB

Telephone: 020 7377 7236

Fax: 020 7377 7003

Email: r.c.thuraising@qmul.ac.uk

Name: Dr Matthew Buckland

Address: Clinical Transplantation Laboratory, 2nd floor, Pathology & Pharmacy Building, 80 Newark Street, The Royal London Hospital, London E1 1BB

Telephone: 020 3246 0264

Fax:

Email: Matthew.Buckland@bartshealth.nhs.uk

Name: Dr Michael Picton

Address: Dept of Renal Medicine, Manchester Royal Infirmary, Oxford Road, Manchester, M13 9WL

Telephone: 0161 276 4253

Fax:

Email: Michael.Picton@cmft.nhs.uk

Name: Dr Declan deFreitas

Address: Dept of Renal Medicine, Manchester Royal Infirmary, Oxford Road, Manchester, M13 9WL

Telephone: 0161 276 7902

Fax:

Email: Declan.deFreitas@cmft.nhs.uk

Name: Dr Judith Worthington

Address: Transplantation Laboratory, Manchester Royal Infirmary, Oxford Road, Manchester, M13 9WL

Telephone: 0161 276 7988

Fax: 0161 276 6148

Email: Judith.Worthington@cmft.nhs.uk

Name: Dr Richard Borrows

Address: Renal Unit, University Hospital Birmingham, Edgbaston, Birmingham, B15 2LN

Telephone: 0121 627 2528

Fax:

Email: Richard.Borrows@uhb.nhs.uk

Name: Dr David Briggs

Address: NHSBT Birmingham, Vincent Drive, Edgbaston, Birmingham B15 2SG.

Telephone: 0121 278 4099

Fax:

Email: David.Briggs@nhsbt.nhs.uk

**Study Synopsis**

| **Title of clinical trial** | **A randomized controlled clinical trial to determine if a combined screening /treatment programme can prevent premature failure of renal transplants due to chronic rejection in patients with HLA antibodies.** |
| --- | --- |
| **Protocol Short Title/Acronym** | **Optimized TacrolimuS and MMF for HLA Antibodies after Renal Transplantation /OuTSMART** |
| **Study Phase if not mentioned in title** | **Phase IV** |
| **Sponsor name** | **King’s College London / GST NHS Foundation Trust** |
| **Chief Investigator** | **Prof. Anthony Dorling** |
| **Eudract number** | **2012-004308-36** |
| **REC number** | **12/LO/1759:** |
| **Medical condition or disease under investigation** | **Premature allograft failure / Chronic rejection** |
| **Purpose of clinical trial** | **The overall objective is to test whether a structured screening programme to identify patients with a validated prognostic biomarker for kidney transplant failure, allied with an optimized immunosuppression treatment protocol, can reduce the time to graft failure at the primary endpoint (approximately 43 months post-randomisation).** |
| **Primary objective** | **Compare the time to graft failure in patients with HLA Ab who receive an optimized anti-rejection medication intervention (‘treatment’), with that in a control group with HLA Ab who remain on their established immunotherapy and whose clinicians are not aware of their Ab status.** |
| **Secondary objective (s)** | **a) Determine the time to graft failure in patients randomized to ‘unblinded’ HLA Ab screening, compared to a control group randomized to ‘blinded’ HLA Ab screening.**  **b) Determine whether treatment influences patient survival**  **c) Determine whether ‘treatment’ influences the development of graft dysfunction as assessed by presence of proteinuria (Protein:Creatinine Ratio > 50 or Albumin:Creatinine Ratio > 35) and change in estimated Glomerular Filtration Rate (eGFR).**  **d) Determine whether ‘treatment’ influences the rates of acute rejection in these groups**  **e) Determine the adverse effect profiles of ‘treatment’ in this group, in particular whether they are associated with increased risk of infection, malignancy or DM.**  **f) Determine the cost effectiveness of routine screening for HLA Ab and prolonging transplant survival using this screening/treatment protocol.**  **g) Determine the impact of biomarker screening and “treatment” on the patients’ adherence to drug therapy and their perceptions of risk to the health of the transplant.** |
| **Trial Design** | **A prospective, open labelled, randomised marker-based strategy (hybrid) trial design, with two arms stratified by biomarker (HLA Ab) status. Recruitment will take place in 13 renal transplant units, recruiting for minimum of 45 months with recruits followed up intensively for 32 months (maximum 64 months) and primary endpoint assessed by remote evaluation when approximately 43 months post-randomisation is achieved by all.** |
| **Endpoints** | **Primary: Time to graft failure in HLA Ab positive patients randomized to biomarker-led treatment groups vs. time to graft failure in HLA Ab positive patients randomized to the control (standard care) group. Graft failure will be defined as re-starting dialysis or requiring a new transplant.**  **Secondary:**  **Clinical:**  **• time to graft failure in patients randomized to unblinded HLA Ab screening vs. blinded screening**  **• patient survival.**  **• graft dysfunction, as assessed by two separate measures; presence of proteinuria (Protein:Creatinine Ratio > 50 or Albumin:Creatinine Ratio > 35) and change in estimated Glomerular Filtration Rates over 32 months.**  **• rates of biopsy-proven T cell-mediated or antibody-mediated rejection over 32 months.**  **• rates of culture-positive infection, biopsy-proven malignancy and diabetes mellitus.**  **• health economic analysis of outcomes in intervention vs. control groups.**  **• analysis of adherence and perceptions of risk in biomarker led care vs standard care groups.** |
| **Sample Size** | **It is anticipated that approximately 2357 total patients will need to be recruited. Given the observed proportions of DSA participants, predicted drop outs and HLA Ab conversion rates, this will allow the target of 165 (~83 per group) DSA participants to be recruited. It is expected based on observed proportions, that this will result in approximately 824 (412 per group) non-DSA participants being recruited and 1368 (684 per group) participants remaining HLA Ab negative at the primary endpoint, exceeding the target numbers required for these groups.** |
| **Summary of eligibility criteria** | **Included: Renal transplant recipients aged 18-75, > 1 year post-transplantation, with estimated glomerular filtration rate (GFR) ≥30 by 4 variable MDRD.**  **Excluded: Recipients of cross-match positive transplant requiring HLA desensitization to remove antibody, recipients of additional solid organ transplants (e.g. pancreas, heart etc), history of malignancy (with exclusions), recent acute rejection, recipients with hepatitis B, C or HIV, recipients known to have HLA antibody who have received specific treatment, known hypersensitivity to any of the IMPs, known hereditary disorders of carbohydrate metabolism, pregnancy, females who refuse to consent to using suitable contraception through trial, patients enrolled in any other studies involving administration of another IMP at time of recruitment.** |
| **IMP, dosage and route of administration** | **Oral Tacrolimus od or bd titrated to pre-dose levels of 4-8ng/ml.**  **Oral Mycophenolate Mofetil or enteric coated mycophenolic acid bd, tds or qds given at highest tolerated daily dose or according to unit guidelines, with maximum dose determined by SmPC.**  **Oral Prednisone od according to the following regime: 20mg od for 2 weeks tapering to 5mg od over 4 weeks.** |
| **Active comparator product(s)** | **None** |
| **Maximum duration of treatment of a Subject** | **HLA Ab-screening phase will last 45 months. For each recruit, the duration of study will be a minimum of 32 months and up to 64 months, as patients who initially tested negative for HLA Ab, but become HLA Ab positive in the final screening round will be followed up for a further 32 months from that point.** |

**CONTENTS**

**Trial Identifiers 42**

**Sponsor 42**

**Chief Investigator 42**

**Name and address of Co-Investigator(s), Statistician, Laboratories etc 42**

**Study Synopsis 44**

**1. Background & Rationale 49**

*1.1 Existing Research 49*

1.1.1 Using HLA Ab (a prognostic biomarker of premature kidney transplant failure) as a screening test: 49

1.1.2: The biology of HLA Ab and CR: 50

1.1.3: Evidence supporting the use of optimized oral immunotherapy regime in patients with HLA Ab: 50

*1.2. Risks and Benefits 51*

1.2.1: Risks: 51

1.2.2: Benefits: 52

*1.3. Rationale for Current Study 52*

*1.4. References 52*

**2. Trial Objectives and Design 55**

*2.1. Trial Objectives 55*

2.1.1 Primary endpoints 55

2.1.2 Secondary endpoints 55

*2.2 Trial Design & Flowchart 56*

2.2.1 Table of events - Summary of study procedures 57

*2.3 Trial Flowchart 59*

**3 Trial Medication 60**

*3.1 Investigational Medical Products and dosing regimen 60*

*3.2 Drug Accountability 60*

*3.3 Subject Compliance 60*

*3.4 Concomitant Medication 60*

**4. Selection and Withdrawal of Subjects 60**

*4.1 Inclusion Criteria 60*

*4.2 Exclusion Criteria 60*

*4.3 Selection of Participants 61*

*4.4 Randomisation Procedure / Code Break 61*

*4.5 Withdrawal of Subjects 62*

*4.6 Expected Duration of Trial 62*

**5. Trial Procedures 63**

*5.1 By Visit 63*

*5.2 Laboratory Tests 65*

5.2.1 HLA Ab analysis: 65

5.2.2 Routine biochemical, haematological and calcineurin inhibitor trough analysis: 66

5.2.3 Scientific laboratory analysis: 66

**6 Assessment of Efficacy 66**

*6.1.1 Primary Efficacy Parameters 66*

*6.1.2 Secondary Efficacy Parameters 66*

*6.2 Procedures for Assessing Efficacy Parameters 66*

**7. Assessment of Safety 67**

*7.1 Specification, Timing and Recording of Safety Parameters. 67*

*7.2 Procedures for Recording and Reporting Adverse Events 68*

*7.3 Reporting Responsibilities 69*

7.3.1 Adverse events that do not require reporting 69

*7.4 Treatment Stopping Rules 70*

**8. Statistics 70**

*8.1 Sample Size 70*

*8.2 Randomisation 72*

*8.3 Analysis 72*

**9. Trial Steering Committee 73**

**10. Data Monitoring Committee 73**

**11. Direct Access to Source Data and Documents 74**

**12. Ethics & Regulatory Approvals 74**

**13. Quality Assurance 74**

**14. Data Handling, Publication Policy and Finance 74**

**15. Data Handling 74**

**16. Publication Policy 75**

**17. Insurance / Indemnity 75**

**18. Financial Aspects 75**

**19. Signatures 75**

**Appendix 1 76**

**1. Background & Rationale**

The problem addressed by this study is ‘premature’ transplant failure – kidney transplants do not last for the natural lifespan of most recipients. Premature in this context refers to the lifespan of the recipient^^[[1]](#footnote-1)^^. Current death-censored 10-year transplant survival rates vary between 59 and 70%, so 30-40% of patients have their transplant for < 10 years [[1](#_ENREF_1)]. Since 2000, a consistent annual attrition rate of around 3% of kidney transplants [[2](#_ENREF_2)] means that approximately 700 patients return to dialysis each year in the UK. Attrition rates in the USA are similar [[1](#_ENREF_1)], so this is a worldwide problem. Although many of these patients are eligible for a second transplant, the legacy of the first often makes it harder to find a well-matched second kidney. In addition, second (and any subsequent) transplants have a shorter lifespan than the original transplant, so the problem of premature failure becomes amplified. Of the various reasons why transplanted kidneys fail the single biggest cause is immune-mediated injury [[3](#_ENREF_3)].

**1.1 Existing Research**

**1.1.1 Using HLA Ab (a prognostic biomarker of premature kidney transplant failure) as a screening test:**

Two types of study have linked antibodies (Ab) against human leukocyte antigens (HLA) to immune-mediated injury and premature graft failure. Case control studies have compared patients who have lost grafts with those in whom grafts are still working, performing retrospective analysis of prospectively collected serum samples. For instance, Mizutani et al [[4](#_ENREF_4)] studied 39 patients with failed grafts due to ‘chronic rejection’ (CR) and 26 matched controls with functioning grafts. In the former group, 72% had IgG HLA Ab, compared to 46% of controls. Similar results were obtained from a different study of a separate population [[5](#_ENREF_5)]. The surprising thing from these studies was the high incidence of HLA Ab in patients with working grafts. There are several potential explanations for this. First, it is possible that factors about the HLA Ab (such as complement fixing ability) or factors other than the Ab influence the progression of CR and thus the timing of eventual graft rejection. Our data supports the latter, by linking progression of renal dysfunction to activity of the cellular immune responses (see appendices). A second, related possibility, is that all patients with HLA Ab develop pathology, but progressing at different rates, such that patients showing up in the control groups in these studies are deteriorating more slowly. Evidence for this comes from Mizutani [[4](#_ENREF_4)], who showed that their CR group with HLA Ab showed progressive deterioration of renal function prior to graft failure. The same progressive deterioration was seen in the control group of patients with HLA Ab, whose grafts did not fail. These data illustrate that CR is a time-dependent process in which progressive graft dysfunction precedes graft failure. Moreover, the time from development of HLA Ab to graft failure is highly variable in different people.

Separate studies have reported prospective follow-up of outcomes in those with HLA Ab. Terasaki et al [[6](#_ENREF_6)] studied 2231 patients. In the group of 479 with HLA Ab, the two-year graft failure rate was 15%, compared to 6.8% in the 1753 with no HLA Ab. This trial noted that the patients who failed within two years had worse renal function on testing than those that didn’t, consistent with the fact that CR is a progressive and time-dependent process and those that fail are at the end of this process. In another study, the same group [[7](#_ENREF_7)] reported 4 year survival rates in 1329 patients, all with functioning transplants, of 58% in those with HLA Ab (158 patients) vs. 81% in those without (806). Lachmann et al [[8](#_ENREF_8)] have performed the best study to date, of 1014 patients with stable kidney function (for the six months pre-recruitment) from a single centre in Berlin, on average 6 years post-transplantation, who were tested for HLA Ab and prospectively followed for 5½ years. Grafts failed in 37% of the 302 who had HLA Ab, but in only 17% of the 712 patients who tested negative for HLA Ab. Moreover, in this latter group, a subgroup of 195 patients had a repeat test performed 2 ½ years into the study; of these, 148 remained negative and only 6% of grafts failed in this group. In contrast, 47 had developed new HLA Ab since the beginning of the study and 21% of these suffered graft failure, confirming that the development of new HLA Ab in the ‘negative’ group was predictive of future graft loss. Finally, this study identified a difference between the prognostic value of HLA Ab that were specific for the donor (donor specific antibodies – DSA, found in 33% of HLA Ab positive patients) and those that were not (non-DSA, found in 66%). Graft failure rates were 51% over 5.5 years in patients with DSA and 30% in patients with non-DSA. In subgroup of patients who had transplant biopsies, 78% of those with failed grafts and HLA Ab+ had changes consistent with CR. They concluded that grafts in patients with HLA Ab were >3x more likely to fail than those without, even when corrected for age, gender, year of transplant, estimated GFR and number of previous kidney transplants. These findings have been corroborated by a second study from the Netherlands [[9](#_ENREF_9)], in which the risk of graft failure with HLA Ab was also shown to be independent of graft dysfunction and proteinuria.

**1.1.2: The biology of HLA Ab and CR:**

Although there is a widespread view in the literature is that HLA Ab *cause* CR, there is no evidence of this in humans. We have data indicating that T cell responses, via specific interaction with donor-specific B cells, drive the progressive deterioration in a subgroup of patients with CR and HLA Ab (see appendices).

Donor HLA are the primary target of the immune response against the transplanted kidney and it is for this reason that HLA matching pre-transplantation still underpins organ allocation, to maximise the similarities between donor and recipient HLA.

The prevailing view in the literature is that when HLA Ab are present, they cause the pathology that ultimately leads to graft dysfunction and kidney failure – indeed, one type of CR is called chronic antibody-mediated rejection (CAMR), exemplifying how Ab are thought to contribute to the process [[10](#_ENREF_10)] (see appendix 1). This is easy to conceptualise when the HLA Ab detected in the circulation are donor specific (i.e. DSA), but less easy when only non-DSA are found, though conventional wisdom has it that the DSA in these patients are all deposited within the graft [[11](#_ENREF_11)]. However, although Ab against the mouse equivalent of HLA have been shown to induce some of the features of CR in experimental models, there is no clinical evidence to support a causative role for HLA Ab in human CR.

In reality, the pathophysiology of human CR is likely to be complex. The presence of HLA Ab indicates that the recipient’s immune system has recognised and reacted to donor HLA. Although the Abs are made by plasma cells, these arise from activated antigen-specific B cells through a process that involves intimate contact between the B cells and HLA-specific T cells. Thus multiple components of the recipient immune system, including T and B cells are sensitised against the donor in patients with HLA Ab and there are several mechanisms capable of damaging the transplanted organ that may be operating in these patients.

The chief investigator’s group has been investigating patients with CR for several years. This work has been seeking the answers to two basic questions; which elements of the immune system are involved in the pathology of CR and what is driving the deterioration in kidney function? Data from these studies indicates that in patients with HLA Ab, the activity of T and B cells is most strongly associated with progression of CR. For example, 15 patients with early 'Ab-mediated' injury on protocol kidney biopsy were followed for 2.5 years, during which 6/15 developed clinical evidence of CR, manifesting as a progressively deteriorating creatinine or development of significant proteinuria. Examining the peripheral blood mononuclear cells of these patients, interferon-gamma (IFN-γ) production by T cells at the time of biopsy was most strongly associated with development of deteriorating function, compared to multiple other variables examined, including HLA Ab. In 75% of these people, B cells were helping CD4+ T cells to make the IFN-γ, consistent with hypothesis that the close interaction between antigen-specific T and B cells is at the centre of the mechanisms operating in the deteriorating transplants in these patients (see appendix 1).

**1.1.3: Evidence supporting the use of optimized oral immunotherapy regime in patients with HLA Ab:**

The data from the CI, referred to above suggests that it would be more rational to target underlying cellular immune responses (i.e. by T and B cells) to prevent CR, rather than focus on the Ab. Based on this, a strategy to enhance immunosuppression to target T and B cells was tested in a group of >30 patients with established CR, all of whom had deteriorating transplant function. 18 of these, with relatively homogenous pathology on their renal biopsies, received ‘optimized treatment’ with tacrolimus (Tac), MMF and in a few, rituximab. After this intervention, 11/18 were assessed to have stabilised 1 year later. However, at 3 years, only 7/18 remained stable. Analysis of T and B cell activity at the time of biopsy indicates that donor-specific IFN-γ production by T cells strongly associated with long-term stability, indicating that these patients benefit most from the enhanced immunosuppression (appendix 1). In addition to this work, the CI is also running a randomized controlled trial (RituxiCAN-C4) in patients with advanced CR, and this has a run-in phase in which all patients get optimized on Tac and MMF. Though still ongoing, it is clear from the interim analyses that up to 30-50% of the patients stabilize during this run-in phase, some of them remaining stable for more than 3 years (appendix 2). The patients who appear to benefit are those with the least advanced CR, suggesting that, if caught early, optimized oral therapy to suppress T and B cells may be sufficient to control the underlying pathological process.

Research by others working in this area is mostly focussed on how to treat established CR. All reports contain small numbers of patients. Theruvath et al reported 12-month stabilisation of kidney function in ¾ patients with kidney dysfunction due to CR associated with HLA Ab after transfer onto Tac and MMF and a short course of prednisolone [[12](#_ENREF_12)]. In addition, several studies have reported successful stabilization of CR using B cell depletional therapy [[13-15](#_ENREF_13)], further supporting the hypothesis that targeting underlying cellular responses, rather than HLA Ab, is a rational approach to treatment in these patients.

In contrast to these, this trial will involve optimization of therapy in many patients before they have started to clinically deteriorate, to try and prevent the graft dysfunction associated with CR. As described above, the natural history of untreated CR is of progressive loss of function, usually at a predictable rate, leading eventually to complete loss of graft function. Depending on the starting creatinine, the time to graft loss will be variable, but graft loss rarely occurs without this period of progressive loss of function. Lachmann et al [[8](#_ENREF_8)] reported a constant rate of graft loss from the start of their study, so we expect that the rate of graft loss in our study to be constant in the standard care group. We predict that optimised immunotherapy will change the natural history of the condition, and lead to stabilisation of function in a significant proportion (50%) of those with HLA Ab. This will be visible in our analyses of the secondary endpoints. Hopefully it will prevent the predicted graft loss in the first 3 years in this group and thus impact on the primary endpoint of the study.

Several other strands of evidence support the use of an optimized Tac and MMF regime to achieve this. First, both MMF [[16](#_ENREF_16)] and Tac [[17](#_ENREF_17)] are better at suppressing acute rejection than alternative agents and the combination of the two agents achieves better outcomes at 1 and 2 years compared to alternative regimes [[18](#_ENREF_18), [19](#_ENREF_19)]. Second, regimes containing MMF are associated with a lower prevalence of HLA Ab [[20](#_ENREF_20)] and MMF specifically reduces the development of HLA Ab development during episodes of acute rejection [[21](#_ENREF_21)]. Third, although these benefits have not always fed through to improvements in graft survival rates, one recent landmark study did show improved graft survival on the combination of Tac and MMF ([[22](#_ENREF_22)]. For Tac, enhanced graft survival also emerged during a systematic Cochrane review comparing Tac with ciclosporin (CsA) [[19](#_ENREF_19)]. For MMF, a retrospective analysis of US registry data revealed an association with significantly lower rates of premature allograft failure [[23](#_ENREF_23)]. Finally, some studies have shown that conversion from CsA to Tac is beneficial in patients with deteriorating graft function [[24](#_ENREF_24)], and that introduction of MMF has a similar effect [[25](#_ENREF_25)]. Although other studies have reported contradictory results, [[26](#_ENREF_26)] much of this literature is difficult to interpret because many studies do not distinguish between CR and other causes of chronic graft dysfunction [[27](#_ENREF_27)].

In addition to Tac and MMF, we propose to use a short course of moderate-dose prednisolone followed by low dose steroid maintenance in this trial. There is no direct evidence from the transplant literature to support this intervention, but a similar treatment course is standard therapy in many situations where quick and effective suppression of immune responses is required, for example in acute asthma and in many types of autoimmune diseases.

**1.2. Risks and Benefits**

**1.2.1: Risks:**

Conversion to Tac from CsA is associated with an increased risk of diabetes mellitus (DM), reported in one study as occurring in 12.5% compared to 4.5% in those who remained on CsA [[24](#_ENREF_24)]. Analysis of >130 trials comparing the two agents revealed that Tac is associated with a 5% higher rate of DM compared to CsA [[19](#_ENREF_19)]. This risk is likely to be exacerbated by the prednisolone, although the DM induced by combined pred/Tac is only transient in approximately 50% of patients. Enhanced immunosuppression is associated with an increased incidence of infection, especially viral and with an increased risk of malignancy. The precise risks of both these in this trial are difficult to estimate. The incidence of DM, infection and malignancy will be monitored carefully on this trial.

**1.2.2: Benefits:**

Tac is associated with a better cholesterol profile [[28](#_ENREF_28)] and lower blood pressures [[19](#_ENREF_19)] than CsA.

**1.3. Rationale for Current Study**

Spending on kidney failure services is 3% of the total NHS budget. The National Service Framework recognizes transplantation as the most clinically and cost effective treatment for patients with kidney failure. To maximise rates of transplantation, efforts are focussed on increasing the number of donor organs by 50% in the next few years. In the words of the NHSBT, “The cost benefit of kidney transplantation compared to dialysis over a period of ten years is £241,000.” The problem of premature transplant failure undermines the ability of the NHS to capitalise maximally on improved transplantation rates and is an aspect hitherto ignored by health strategists.

Because CR is the single biggest cause of premature graft failure and because HLA Ab are an established prognostic biomarker for premature graft failure, there is a need to test whether treatment decisions based on the presence of the biomarker can alter prognosis. So this trial combines 2 elements, testing whether a routine screening programme for HLA Ab in all kidney transplant recipients is useful, and then, for those found to be HLA Ab +, testing whether the (randomized) introduction of a standard optimization treatment protocol can reduce graft failure rates.

Management in both the biomarker-led care (BLC) and standard care (SC) groups will involve control of hypertension, proteinuria and hypercholesterolaemia to defined target ranges using conventional agents. Clinicians will be blinded to the results of the screening tests in the ‘blinded’ groups. Patients who have no HLA Ab at their initial screening will be re-screened every 8 months for the first three years of the study. If found to be HLA Ab+, those in the ‘unblinded’ group (D) will enter the standardized anti-rejection optimized treatment protocol, whereas the treatment of those in the ‘blinded’ group (C) will be unchanged and clinicians will remain unaware of the change in HLA Ab status.

**1.4. References**

1 **Lamb, K. E., Lodhi, S. and Meier-Kriesche, H. U.,** Long-term renal allograft survival in the United States: a critical reappraisal. *Am J Transplant* 2011. **11**: 450-462.

2 **Ravanan, R., Udayaraj, U., Bakran, A., Steenkamp, R., Williams, A. J. and Ansell, D.,** Measures of care in adult renal transplant recipients in the United Kingdom (chapter 11). *Nephrol Dial Transplant* 2007. **22 Suppl 7**: vii138-154.

3 **Gaston, R. S., Cecka, J. M., Kasiske, B. L., Fieberg, A. M., Leduc, R., Cosio, F. C., Gourishankar, S., Grande, J., Halloran, P., Hunsicker, L., Mannon, R., Rush, D. and Matas, A. J.,** Evidence for antibody-mediated injury as a major determinant of late kidney allograft failure. *Transplantation* 2010. **90**: 68-74.

4 **Mizutani, K., Terasaki, P., Rosen, A., Esquenazi, V., Miller, J., Shih, R. N., Pei, R., Ozawa, M. and Lee, J.,** Serial ten-year follow-up of HLA and MICA antibody production prior to kidney graft failure. *Am J Transplant* 2005. **5**: 2265-2272.

5 **Lee, P. C., Zhu, L., Terasaki, P. I. and Everly, M. J.,** HLA-specific antibodies developed in the first year posttransplant are predictive of chronic rejection and renal graft loss. *Transplantation* 2009. **88**: 568-574.

6 **Terasaki, P. I. and Ozawa, M.,** Predictive value of HLA antibodies and serum creatinine in chronic rejection: results of a 2-year prospective trial. *Transplantation* 2005. **80**: 1194-1197.

7 **Terasaki, P. I., Ozawa, M. and Castro, R.,** Four-year follow-up of a prospective trial of HLA and MICA antibodies on kidney graft survival. *Am J Transplant* 2007. **7**: 408-415.

8 **Lachmann, N., Terasaki, P. I., Budde, K., Liefeldt, L., Kahl, A., Reinke, P., Pratschke, J., Rudolph, B., Schmidt, D., Salama, A. and Schonemann, C.,** Anti-human leukocyte antigen and donor-specific antibodies detected by luminex posttransplant serve as biomarkers for chronic rejection of renal allografts. *Transplantation* 2009. **87**: 1505-1513.

9 **van Timmeren, M. M., Lems, S. P., Hepkema, B. G. and Bakker, S. J.,** Anti-human leukocyte antigen antibodies and development of graft failure after renal transplantation. *Transplantation* 2009. **88**: 1399-1400.

10 **Colvin, R. B.,** Pathology of chronic humoral rejection. *Contrib Nephrol* 2009. **162**: 75-86.

11 **Terasaki, P. I.,** Humoral theory of transplantation. *Am J Transplant* 2003. **3**: 665-673.

12 **Theruvath, T. P., Saidman, S. L., Mauiyyedi, S., Delmonico, F. L., Williams, W. W., Tolkoff-Rubin, N., Collins, A. B., Colvin, R. B., Cosimi, A. B. and Pascual, M.,** Control of antidonor antibody production with tacrolimus and mycophenolate mofetil in renal allograft recipients with chronic rejection. *Transplantation* 2001. **72**: 77-83.

13 **Billing, H., Rieger, S., Ovens, J., Susal, C., Melk, A., Waldherr, R., Opelz, G. and Tonshoff, B.,** Successful treatment of chronic antibody-mediated rejection with IVIG and rituximab in pediatric renal transplant recipients. *Transplantation* 2008. **86**: 1214-1221.

14 **Fehr, T., Rusi, B., Fischer, A., Hopfer, H., Wuthrich, R. P. and Gaspert, A.,** Rituximab and intravenous immunoglobulin treatment of chronic antibody-mediated kidney allograft rejection. *Transplantation* 2009. **87**: 1837-1841.

15 **Rostaing, L., Guilbeau-Frugier, C., Fort, M., Mekhlati, L. and Kamar, N.,** Treatment of symptomatic transplant glomerulopathy with rituximab. *Transpl Int* 2009. **22**: 906-913.

16 **Halloran, P., Mathew, T., Tomlanovich, S., Groth, C., Hooftman, L. and Barker, C.,** Mycophenolate mofetil in renal allograft recipients: a pooled efficacy analysis of three randomized, double-blind, clinical studies in prevention of rejection. The International Mycophenolate Mofetil Renal Transplant Study Groups. *Transplantation* 1997. **63**: 39-47.

17 **Sola, R., Diaz, J. M., Guirado, L., Sainz, Z., Gich, I., Picazo, M., Garcia, R., Abreu, E., Ortiz, F. and Alcaraz, A.,** Tacrolimus in induction immunosuppressive treatment in renal transplantation: comparison with cyclosporine. *Transplant Proc* 2003. **35**: 1699-1700.

18 **Ahsan, N., Johnson, C., Gonwa, T., Halloran, P., Stegall, M., Hardy, M., Metzger, R., Shield, C., 3rd, Rocher, L., Scandling, J., Sorensen, J., Mulloy, L., Light, J., Corwin, C., Danovitch, G., Wachs, M., VanVeldhuisen, P., Salm, K., Tolzman, D. and Fitzsimmons, W. E.,** Randomized trial of tacrolimus plus mycophenolate mofetil or azathioprine versus cyclosporine oral solution (modified) plus mycophenolate mofetil after cadaveric kidney transplantation: results at 2 years. *Transplantation* 2001. **72**: 245-250.

19 **Webster, A., Woodroffe, R. C., Taylor, R. S., Chapman, J. R. and Craig, J. C.,** Tacrolimus versus cyclosporin as primary immunosuppression for kidney transplant recipients. *Cochrane Database Syst Rev* 2005: CD003961.

20 **Lederer, S. R., Friedrich, N., Banas, B., Welser, G., Albert, E. D. and Sitter, T.,** Effects of mycophenolate mofetil on donor-specific antibody formation in renal transplantation. *Clin Transplant* 2005. **19**: 168-174.

21 **van der Mast, B. J., van Besouw, N. M., Witvliet, M. D., de Kuiper, P., Smak Gregoor, P., van Gelder, T., Weimar, W. and Claas, F. H.,** Formation of donor-specific human leukocyte antigen antibodies after kidney transplantation: correlation with acute rejection and tapering of immunosuppression. *Transplantation* 2003. **75**: 871-877.

22 **Ekberg, H., Tedesco-Silva, H., Demirbas, A., Vitko, S., Nashan, B., Gurkan, A., Margreiter, R., Hugo, C., Grinyo, J. M., Frei, U., Vanrenterghem, Y., Daloze, P. and Halloran, P. F.,** Reduced exposure to calcineurin inhibitors in renal transplantation. *N Engl J Med* 2007. **357**: 2562-2575.

23 **Ojo, A. O., Meier-Kriesche, H. U., Hanson, J. A., Leichtman, A. B., Cibrik, D., Magee, J. C., Wolfe, R. A., Agodoa, L. Y. and Kaplan, B.,** Mycophenolate mofetil reduces late renal allograft loss independent of acute rejection. *Transplantation* 2000. **69**: 2405-2409.

24 **Meier, M., Nitschke, M., Weidtmann, B., Jabs, W. J., Wong, W., Suefke, S., Steinhoff, J. and Fricke, L.,** Slowing the progression of chronic allograft nephropathy by conversion from cyclosporine to tacrolimus: a randomized controlled trial. *Transplantation* 2006. **81**: 1035-1040.

25 **Meier-Kriesche, H. U., Merville, P., Tedesco-Silva, H., Heemann, U., Kes, P., Haller, H., Rostaing, L., Gafner, N. and Bernasconi, C.,** Mycophenolate mofetil initiation in renal transplant patients at different times posttransplantation: the TranCept Switch study. *Transplantation* 2011. **91**: 984-990.

26 **Jevnikar, A., Arlen, D., Barrett, B., Boucher, A., Cardella, C., Cockfield, S. M., Rush, D., Paraskevas, S., Shapiro, J., Shoker, A., Yilmaz, S., Zaltzman, J. S. and Kiberd, B.,** Five-year study of tacrolimus as secondary intervention versus continuation of cyclosporine in renal transplant patients at risk for chronic renal allograft failure. *Transplantation* 2008. **86**: 953-960.

27 **Remuzzi, G., Cravedi, P., Costantini, M., Lesti, M., Ganeva, M., Gherardi, G., Ene-Iordache, B., Gotti, E., Donati, D., Salvadori, M., Sandrini, S., Segoloni, G., Federico, S., Rigotti, P., Sparacino, V. and Ruggenenti, P.,** Mycophenolate mofetil versus azathioprine for prevention of chronic allograft dysfunction in renal transplantation: the MYSS follow-up randomized, controlled clinical trial. *J Am Soc Nephrol* 2007. **18**: 1973-1985.

28 **Cheung, C. Y., Chan, H. W., Liu, Y. L., Chau, K. F. and Li, C. S.,** Long-term graft function with tacrolimus and cyclosporine in renal transplantation: paired kidney analysis. *Nephrology (Carlton)* 2009. **14**: 758-763.

29 **Dudley, C., Pohanka, E., Riad, H., Dedochova, J., Wijngaard, P., Sutter, C. and Silva, H. T., Jr.,** Mycophenolate mofetil substitution for cyclosporine a in renal transplant recipients with chronic progressive allograft dysfunction: the "creeping creatinine" study. *Transplantation* 2005. **79**: 466-475.

30 **Curtis, L.,** Unit Costs of Health and Social Care 2010. PSSRU, University of Kent, Canterbury 2010.

31 **Mora, P. A., Berkowitz, A., Contrada, R. J., Wisnivesky, J., Horne, R., Leventhal, H. and Halm, E. A.,** Factor structure and longitudinal invariance of the Medical Adherence Report Scale-Asthma. *Psychol Health* 2011. **26**: 713-727.

32 **Cohen, J. L., Mann, D. M., Wisnivesky, J. P., Home, R., Leventhal, H., Musumeci-Szabo, T. J. and Halm, E. A.,** Assessing the validity of self-reported medication adherence among inner-city asthmatic adults: the Medication Adherence Report Scale for Asthma. *Ann Allergy Asthma Immunol* 2009. **103**: 325-331.

33 **Butler, J. A., Peveler, R. C., Roderick, P., Smith, P. W., Horne, R. and Mason, J. C.,** Modifiable risk factors for non-adherence to immunosuppressants in renal transplant recipients: a cross-sectional study. *Nephrol Dial Transplant* 2004. **19**: 3144-3149.

34 **Butler, J. A., Peveler, R. C., Roderick, P., Horne, R. and Mason, J. C.,** Measuring compliance with drug regimens after renal transplantation: comparison of self-report and clinician rating with electronic monitoring. *Transplantation* 2004. **77**: 786-789.

35 **Haynes, R. B., Taylor, D. W., Sackett, D. L., Gibson, E. S., Bernholz, C. D. and Mukherjee, J.,** Can simple clinical measurements detect patient noncompliance? *Hypertension* 1980. **2**: 757-764.

36 **Gordis, L.,** Conceptual and methodologic problems in measuring patient compliance. . In **Sackett, D. L., Taylor, D. W. and R.B., H.** (Eds.) *Compliance in Health Care*. John Hopkins University Press, London 1979, pp 23-45.

37 **Horne, R., Weinman, J. and Hankins, M.,** The beliefs about medicines questionnaire: The development and evaluation of a new method for assessing the cognitive representation of medication. *Psychology & Health* 1999. **14**: 1-24.

38 **Horne, R., Hankins, M. and Jenkins, R.,** The Satisfaction with Information about Medicines Scale (SIMS): a new measurement tool for audit and research. *Quality in Health Care* 2001. **10**: 135-140.

39 **Zigmond, A. S. and Snaith, R. P.,** THE HOSPITAL ANXIETY AND DEPRESSION SCALE. *Acta Psychiatrica Scandinavica* 1983. **67**: 361-370.

40 **Moss-Morris, R., Weinman, J., Petrie, K. J., Horne, R., Cameron, L. D. and Buick, D.,** The revised Illness Perception Questionnaire (IPQ-R). *Psychology & Health* 2002. **17**: 1-16.

41 **Beatty, P. C. and Willis, G. B.,** Research synthesis: The practice of cognitive interviewing. *Public Opinion Quarterly* 2007. **71**: 287-311.

42 **Barber, J. and Thompson, S.,** Multiple regression of cost data: use of generalised linear models. *J Health Serv Res Policy* 2004. **9**: 197-204.

**2. Trial Objectives and Design**

**2.1. Trial Objectives**

The overall objective is to test whether a structured screening programme to identify patients with a validated prognostic biomarker for kidney transplant failure, allied with an optimized immunosuppression treatment protocol, can reduce transplant failure rates over time.

Primary objective;

Determine the time to graft failure in patients testing positive for HLA Ab at baseline or within 32 months of randomization who receive an optimized anti-rejection medication intervention with prednisone, Tac and MMF (‘treatment’), compared to a control group who test positive for HLA Ab at baseline or within 32 months post-randomization who remain on their established immunotherapy and whose clinicians are not aware of their Ab status. The primary endpoint will be assessed remotely when approximately 43 months post-randomisation has been achieved by all.

Secondary objectives;

a) Determine the time to graft failure in patients randomized to ‘unblinded’ HLA Ab screening, compared to a control group randomized to ‘blinded’ HLA Ab screening.

b) Determine whether ‘treatment’ influences patient survival

c) Determine whether ‘treatment’ influences the development of graft dysfunction as assessed by presence of proteinuria (Protein:Creatinine Ratio > 50 or Albumin:Creatinine Ratio > 35) and change in estimated Glomerular Filtration Rate (eGFR).

d) Determine whether ‘treatment’ influences the rates of acute rejection in these groups

e) Determine the adverse effect profiles of ‘treatment’ in this group, in particular whether they are associated with increased risk of infection, malignancy or DM.

f) Determine the cost effectiveness of routine screening for HLA Ab and prolonging transplant survival using this screening/treatment protocol.

g) Determine the impact of biomarker screening and “treatment” on the patients’ adherence to drug therapy and their perceptions of risk to the health of the transplant.

**2.1.1 Primary endpoints**

The primary endpoint is time to graft failure in HLA Ab positive patients randomized to biomarker led care groups vs. time to graft failure in HLA Ab + patients randomized to standard care groups assessed at approximately 43 months post-randomisation achieved by all. A second capture of primary endpoint data will also be taken at 46-92 months post-randomisation to be used in a sensitivity analysis. Graft failure will be defined as re-starting dialysis or requiring a new transplant.

**2.1.2 Secondary endpoints**

The secondary clinical endpoints are:

• time to graft failure in patients randomized to blinded HLA Ab screening vs those randomized to unblinded screening. Graft failure will be defined as re-starting dialysis or requiring a new transplant.

The following endpoints will be assessed at end of intensive follow up (32 months):

• patient survival.

• graft dysfunction, as assessed by two separate measures proteinuria (Protein:Creatinine Ratio > 50 or Albumin:Creatinine Ratio > 35) and change in estimated Glomerular Filtration Rates, and the rate of progression to graft dysfunction.

• rates of biopsy-proven rejection.

• rates of culture- or polymerase chain reaction (PCR)-positive infection, biopsy-proven malignancy and DM.

• health economic analysis of outcomes in intervention vs. control groups.

• analysis of adherence and perceptions of risk in BLC groups.

**2.2 Trial Design & Flowchart**

This is a prospective, open labelled, randomised marker-based strategy (hybrid) trial design, with two arms stratified by biomarker (HLA Ab) status. Recruitment will take place in 13 renal transplant units, recruiting for 45 months with recruits followed up intensively for at least 32 months (maximum 64 months) and primary endpoint assessed by remote evaluation after approximately 43 months post-randomisation is achieved by all. The trial design is represented in the flow diagram in section 2.3, showing the number of patients anticipated to be in each group by the end of the trial, based on sample size calculations, consent rates, eligibility and estimated fall-out. Using the flow diagram (top-to-bottom) as a guide: recipients of cross-match negative transplants aged 18-75, > 1 year post-transplant with an eGFR ≥ 30 will consent to the screening/treatment process. The first stratification will result from blood test screening for HLA Ab. Approximately 35% will be HLA positive, with ~65% negative. The HLA Ab+ patients will be further screened with single antigen beads to determine whether DSA are present (~1/6 DSA and 5/6 non-DSA). Thus, biomarker stratification leads to three groups (DSA+, non-DSA+ and HLA Ab-neg). The second stratification will be based on current immunosuppression, to ensure balanced numbers already on Tac or MMF in each group. The final stratification will be by site. HLA Ab positive patients will be randomized 1:1 into either Blinded Standard Care or Unblinded Biomarker led-care. Patients in the former (groups A1 & A2 in the flow chart in 2.3) will be blind to their biomarker status and will remain on baseline immunotherapy, whereas patients in the latter (groups B1 and B2 in the flow chart) will know their HLA Ab status and will be offered “treatment”. HLA Ab-negative patients will remain on their existing immunotherapy and randomized 1:1 into either Blinded (group C) or Unblinded groups (D), with only the latter knowing their HLA Ab status. Both these groups will receive regular Ab status monitoring for the first 3 years. Those patients who become positive during subsequent screening rounds (~10% per year) will be moved to the appropriate HLA Ab positive groups (DSA+ or non-DSA+) for final data analysis. All patients in group D found to be positive on second or subsequent rounds will be offered the same “treatment” as those patients who were positive in the first screening round, and be intensively followed up for an additional 32 months from the time they become positive. Thus the maximum amount of time any single patient may remain in intensive follow up is 64 months^^[[2]](#footnote-2)^^. New patients will be recruited to the study at each successive screening round.

**2.2.1 Table of events - Summary of study procedures**

| Phase | Peri-Randomization | Post-Randomization | | | | | | | | | | | |  | |
| --- | --- | --- | --- | --- | --- | --- | --- | --- | --- | --- | --- | --- | --- | --- | --- |
|  |  | Unblinded HLA Ab+ groups – Approximate times of assessment (+/- 1 week). Once stabilised, go to month 8 assessment | | | | | | All Groups – Approximate times of assessment  (+/- 3 months) | | | | | | | |
| Study Week/month | Day -56 to 0 | Wk 2 | Wk 4 | Wk 6 | Wk 8 | Wk 10 | Wk 12 | Month  8 | Month 16 | Month  24 | | Month 32 | | ~ 43 months | Month 46-92 |
| Informed consent | x |  |  |  |  |  |  |  |  |  | |  | |  |  |
| Inclusion/Exclusion Criteria | x^^[[3]](#footnote-3)^^ |  |  |  |  |  |  |  |  |  | |  | |  |  |
| Medical History inc. Drugs | x^^[[4]](#footnote-4)^^ |  |  |  |  |  |  |  |  |  | |  | |  |  |
| Transplant / sensitisation Hx | x |  |  |  |  |  |  |  |  |  | |  | |  |  |
| Registration / Demographics | x^^[[5]](#footnote-5)^^ |  |  |  |  |  |  |  |  |  | |  | |  |  |
| Weight / BP | x |  |  |  |  |  |  | x | x | x | | x | |  |  |
| Urine PCR or ACR | x |  |  |  |  |  |  | x | x | x | | x | |  |  |
| Haematology^^[[6]](#footnote-6)^^ | x |  | X |  | x |  | x | x | x | x | | x | |  |  |
| Biochemistry | x^^[[7]](#footnote-7)^^ |  | x^^[[8]](#footnote-8)^^ |  | x^8^ |  | x^8^ | x^^[[9]](#footnote-9)^^ | x^^[[10]](#footnote-10)^^ | x^9^ | | x^10^ | |  |  |
| [Calcineurin inhibitor] trough | x | x^^[[11]](#footnote-11)^^ | X | x | x | x | x | x | x | x | | x | |  |  |
| Total immunoglobulin  (or IgG, IgM +/- IgA) | x |  |  |  |  |  |  |  | x |  | x | |  | |  |
| HLA antibody screening | x^^[[12]](#footnote-12)^^ |  |  |  |  |  |  | x^12^ | x^12^ | x^12^ | x^12^ | |  | |  |
| Apply optimized treatment protocol^^[[13]](#footnote-13)^^ |  | x | X | x | x | X | x |  |  |  |  | |  | |  |
| See Trial-specific Nurse | x |  |  |  |  |  |  | x | x | x | x | |  | |  |
| Record Medications | x |  |  |  |  |  |  | x | x | x | x | |  | |  |
| Adverse Events Form |  | x | X | x | x | X | x | x | x | x | x | |  | |  |
| Questionnaire for analysis of adherence / risk | x |  |  |  |  |  |  |  |  | x |  | |  | |  |
| Questionnaire for health economics | x |  |  |  |  |  |  |  | x |  |  | |  | |  |
| Primary Endpoint  (remote data collection) |  |  |  |  |  |  |  |  |  |  |  | | x | |  |
| Primary Endpoint  (sensitivity analysis; remote data collection) |  |  |  |  |  |  |  |  |  |  |  | |  | | x |

Note: where an “x” is contained within a field this denotes that the associated data will be collected at the identified time point.

2.3 Trial Flowchart


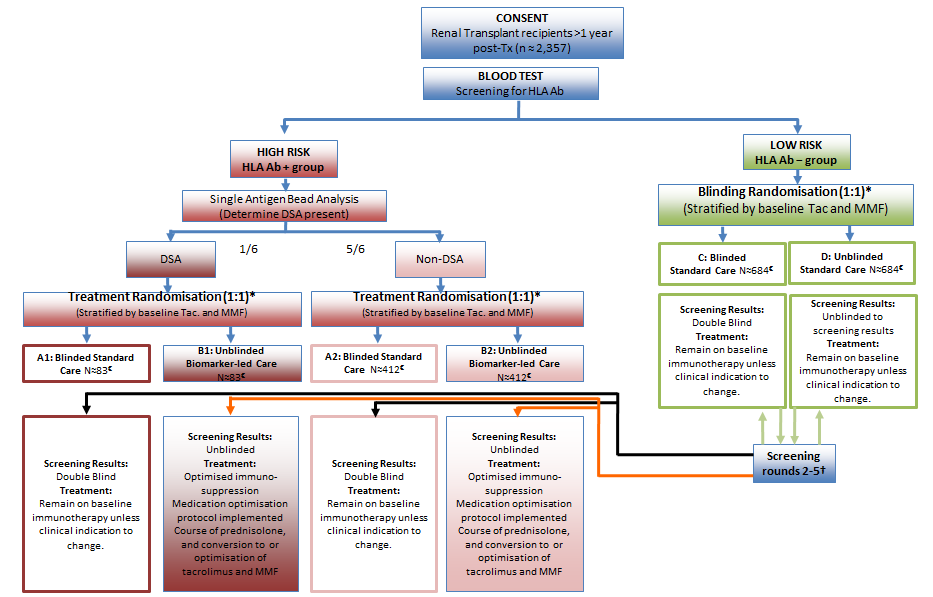


*Randomisation performed on results of a recruit’s first screening test. Those with HLA Ab undergo no further screening as part of the trial (but serum will be stored for analysis of HLA Ab profiles later). **^†^**Those initially HLA Ab-negative undergo routine screening every 8 months. THERE IS NO SECOND RANDOMIZATION: If a recruit allocated to Blinded standard care (group C) becomes HLA Ab positive (black lines), he/she remains in Standard care group (group A1 or A2). If in unblinded standard care group (D), they change to unblinded biomarker-led treatment care (group B1 or B2) (orange lines). € Numbers in each group are those anticipated at the end of study.

**3 Trial Medication**

All treatments will be introduced on the basis that they will be tailored to the individual patient, according to compliance, tolerance and achievement of target levels (for Tac). Failure to tolerate one or more of the components of the protocol (or refusal to take any of the agents) will not be used as a reason for withdrawal from the study.

**3.1 Investigational Medical Products and dosing regimen**

The ‘optimized treatment’ protocol in the two groups (B_1_, B_2_ in section 2.3) with HLA Ab will be;

a) Mycophenolate mofetil bd, tds or qds, or enteric coated mycophenolic acid bd, with daily dose determined according to local unit guidelines. The patient will be stabilized on the maximum tolerated dose.

b) Tacrolimus od or bd, according to local unit preference, with dose titrated to achieve 12-hour post-dose levels of 4μg/L to 8μg/L (4-8 ng/ml). The patient will be stabilized on the maximum tolerated dose that achieves these levels.

c) Prednisolone od. Starting at 20mg for two weeks, then reducing by 5 mg od every two weeks down to their previous maintenance dose or 5mg od, if not previously taking.

After consultation with the MHRA, we have confirmed that all these medicines will be classed as IMPs, whereas all others will not. Mycophenolate mofetil/mycophenolic acid is being used outside of its Marketing Authorisation (which states that it should be used with ciclosporin). However, because it is now used so widely in combination of tacrolimus in most units in the UK, the two can be regarded as ‘standard care’. We therefore propose that the three drugs will not require labelling in line with annex 13. This means the IMPs can be managed in the same way as normal i.e. GP or hospital prescription (as appropriate) and will not require special labelling/accountability/storage etc.

**3.2 Drug Accountability**

Not applicable

**3.3 Subject Compliance**

See section 6.2

**3.4 Concomitant Medication**

Patients in all groups will have blood pressure controlled and total cholesterol lowered, using agents according to local unit guidelines and working to unit-defined targets. All other medication and treatment will be determined by local unit guidelines.

**4. Selection and Withdrawal of Subjects**

**4.1 Inclusion Criteria**

• Sufficient grasp of English to enable written and witnessed informed consent to participate.

• Renal transplant recipients >1 year post-transplantation, male or female

• Aged 18-75 years

• Estimated glomerular filtration rate (eGFR by 4 variable MDRD) of ≥30 (within the previous 6 months of signing consent or taken at screening if not done in the previous 6 months).

**4.2 Exclusion Criteria**

• Recipient requiring HLA desensitisation to remove antibody for a positive XM transplant

• Recipient known already to have HLA antibody WHO HAS RECEIVED specific intervention for that antibody or for CAMR / chronic rejection

• Recipient of additional solid organ transplants (e.g. pancreas, heart, etc).

• History of malignancy in previous 5 years (excluding non-melanomatous tumours limited to skin)

• HBsAg+, HepC IgG+ or HIV+ recipient (on test performed within previous 5 years)

• History of acute rejection requiring escalation of immunosuppression in the 6 months prior to screening.

• Patient enrolled in any other studies involving administration of another IMP at time of recruitment

*The following exclusion criteria are based on information contained within the SMPcs of the IMPs*

• Known hypersensitivity to any of the IMPs

• Known hereditary disorders of carbohydrate metabolism

• Pregnancy or breastfeeding females (based on verbal history of recipient)

• Pre-menopausal females who refuse to consent to using suitable methods of contraception throughout the trial.

**4.3 Selection of Participants**

The local transplant clinic database will be used to identify patients meeting the baseline inclusion/exclusion criteria. At the start of the trial, the entire population of transplant clinic attendees who meet the eligibility criteria are potentially eligible for recruitment. On subsequent screening rounds, patients who reach 12 months post-transplantation after the start of the trial will become eligible and these will be recruited before the next screening round.

Informed consent – Potentially eligible patients will be approached at a routine clinic appointment by the PI or research nurses and given printed and verbal information about the trial. They will have the opportunity to return for a second consultation within a few days to give informed consent for recruitment into the study or to do this on their next routine appointment. Alternatively, eligible patients will be sent information about the study through the post, for discussion and consent at their next routine appointment. Following consent, full eligibility criteria will be reviewed. This may include testing for chronic viral disease (if no such test within last 5 years) or pregnancy (if history suggests possibility of pregnancy).

**4.4 Randomisation Procedure / Code Break**

Prior to randomisation but after consent, site staff will register all recruits online and each will be assigned a MACRO PIN. Samples from all recruits will be sent to the HLA laboratory, along with this PIN and a sample request form containing other information required for randomisation.

Lab staff will screen for HLA Ab and perform single antigen bead testing on positive screening samples to check for the presence of DSA. Once this information is known, the lab staff will access the randomisation system and randomise the patient, using the HLA Ab results and information on the sample form to stratify. In all sites, the PI’s and nurses will be automatically emailed and the system will tell them whether the patient is in the blinded or unblinded groups. If in the unblinded group it will feed back HLA Ab status to the PI. Unblinded patients will be identified by blue stickers to be appended to the notes and all future clinical samples. The system will tell the trial staff to enter HLA Ab-negative patients into the subsequent 8 monthly screening rounds, and also whether the patients have been selected to provide future samples for 8 monthly scientific analysis (for transfer to the CIs laboratory). This information will be relayed using a ‘star’ on the blue labels, appended to their laboratory request forms thereafter.

Blinded patients will have green stickers/labels. HLA Ab status will not be fed back to the PIs or trial staff. A ‘star’ will be used to tell the trial staff which recruits have been selected to provide 8 monthly samples for transfer back to the CIs lab for scientific analysis. All these patients will have samples taken 8 monthly for HLA Ab screening. Once inside the lab, the lab staff will use their knowledge of the HLA status to determine those from HLA Ab-negative patients which will undergo screening. The samples from HLA Ab positive patients will be discarded.

On the second and subsequent HLA Ab screening rounds, the lab staff will update the randomisation system. The lab staff will have 52 days from the date the re-screen sample was taken to perform the HLA Ab re-screen for the required participants. The results from patients in the unblinded groups only will be forwarded to the PI and lab staff, via email. This will indicate whether status has changed and trigger the initiation of the treatment protocol in those that have changed from HLA Ab negative to positive.

Randomisation will be via the online King’s Clinical Trials Unit randomisation system.

Laboratory staff at each recruiting site, with access to HLA Ab results, will be provided with a unique username and password to access the randomisation system. Password access must be authorised by the trial manager in all cases and request directly from sites will not be processed. Access to the system is via [www.ctu.co.uk](http://www.ctu.co.uk), clicking the ‘randomisation – advanced’ link and selecting the OuTSMART Trial.

There are no blinded study medications in the trial so no emergency code break is required. In the event that a study site clinician wishes to be made aware of blinded laboratory results, this must be discussed and agreed with the trial manager and the study Chief Investigator in all cases. It is not anticipated that unblinding in this manner will be required and only in extraordinary circumstances would this be agreed.

**4.5 Withdrawal of Subjects**

Withdrawal and stopping criteria: Individual recruits can withdraw at any time if they wish. Failure to tolerate one or more components of the ‘treatment’ will ***not*** be seen as a reason to withdraw an individual participant from the trial but is to be anticipated as an integral part of individualising therapy. The randomization process will be halted temporarily if any of the following are noted;

• A patient death attributable to ‘treatment’.

• Unacceptable incidence of severe adverse events attributable to ‘treatment’ (if occurring in >10% patients). In both these instances, the trial will undergo urgent review by the Data Monitoring and Ethics and Trial Steering committees.

Participants have the right to withdraw from the study at any time for any reason. The investigator also has the right to withdraw patients from the study drug in the event of inter-current illness, AEs, SAE’s, SUSAR’s, protocol violations, cure, administrative reasons or other reasons. It is understood by all concerned that an excessive rate of withdrawals can render the study uninterpretable; therefore, unnecessary withdrawal of patients should be avoided. Should a patient decide to withdraw from the study, all efforts will be made to report the reason for withdrawal as thoroughly as possible. Should a patient withdraw from study drug only, efforts will be made to continue to obtain follow-up data, with the permission of the patient.

Participants who wish to withdraw from ‘treatment’ will be asked to confirm whether they are still willing to provide study specific data and samples for scientific laboratory analysis according to the trial protocol.

**4.6 Expected Duration of Trial**

The trial is expected to recruit for a minimum of 45 months. The recruitment target is to recruit 165 HLA Ab positive DSA patients, 269 HLA Ab positive non-DSA patients and 243 patients to remain HLA Ab negative until the end of follow up. The targets for DSA and non-DSA include HLA Ab negative participants who develop do-novo antibodies at the 8 monthly re-screening rounds and hence become either HLA Ab positive DSA or HLA Ab positive non-DSA. An estimated 237 patients will need to be recruited overall to recruit the target of 16 DSA patients. Because of this requirement to recruit sufficient DSA participants, recruitment to the other groups is likely to be greater than the specified targets.

Following recruitment to the trial, the patients will undergo 32 months of intensive follow up involving 8-monthly clinic visits post-randomisation, except in the following scenario; a patient in groups C or D who becomes Ab positive during the initial 32 months follow up will transfer to the relevant Ab+ group and undergo intensive follow up for a further 32 months from date of transfer. Therefore, the maximum amount of time that any single patient may remain in intensive follow up is 64 months. The secondary endpoints will be assessed at the end of the intensive follow up period (32 months up to 64 months) and at this point trial procedures relating to the participants will finish. The participants will be informed that they no longer are required to attend research clinic visits. The original plan was for the primary endpoint to be assessed remotely when at least 43 months post-randomisation had been achieved by all. The last participant research clinic visit was expected to be in March 2020 with the assessment of the primary endpoint being performed during the final three months of the trial concluding at the end of June 2020 when the last participant recruited reaches 43 months post-randomisation.

Due to the coronavirus pandemic in the UK in 2020 most clinical trial activity, including this trial, was severely limited and so it was not possible to rely on the original plan to obtain the primary endpoint data between April and June 2020. For this reason, the best alternative was to obtain the primary endpoint data from patients’ clinic notes. Evidence for graft failure or death will be taken from the participants’ last hospital contact prior to March 16 2020. These data, which will reflect participants’ pre-COVID status, will be used for the primary endpoint analysis.

Evidence of graft failure or death will also be taken from participants’ notes from their most recent hospital contact at the point of a final assessment between September 1 2020 and November 30 2020. During this designated three-month window, endpoint data will be collected from each patients’ notes only once. These data, reflecting status post-onset of COVID crisis, will be used for a sensitivity analysis. The trial will conclude on November 30 2020.

The end of trial for this study has been defined as the last follow up of primary outcome data.

**5. Trial Procedures**

Synopsis: A structured screening programme for IgG HLA Ab is proposed in patients who give consent. Results obtained will initially be blinded to the transplant clinicians and patients. DSA+, non-DSA+ and HLA Ab-neg groups will be randomized through the KCL CTU (stratified by current immunosuppression) into the groups as detailed. Patients in groups A1, A2 and C (see flow diagram section 2.3) will remain blinded to the results of their screening (as will their clinicians), whereas those in B1, B2 and D will learn whether they are HLA Ab+ or Ab-neg. All recruits will undergo a final test for HLA Ab status as they reach the end of the study. The ‘optimized treatment’ protocol in the two groups (B_1_, B_2_) with HLA Ab is outlined in section 3 above. Patients in all groups will have blood pressure controlled and total cholesterol lowered, according to local unit guidelines.

All treatments will be introduced on the basis that they will be tailored to the individual patient, according to compliance, tolerance and achievement of target levels (for Tac). Failure to tolerate one or more of the components of the protocol (or refusal to take any of the agents) will not be used as a reason for withdrawal from the study.

**5.1 By Visit**

Post consent, patients who have not been screened for HIV or hepatitis B/C within the last 5 years will need to have additional screening tests for these viruses. Female patients who report they may be pregnant will have a blood or urine test for beta-HCG levels. Once eligibility criteria have been met, the following baseline data will be recorded at recruitment. a) Weight and bp; b) Sex and ethnicity; c) Age and date of birth; d) HLA type and that of donor kidney (if known); e) Any significant past medical history, including history of diabetes mellitus, cause of renal failure, details of previous transplants and cause of graft loss, evidence of sensitisation pre-transplantation (PRA and antibody specificities if known); f) medication list and doses; g) Protein:Creatinine ratio on urine sample;

All patients will then have blood taken for;

i) Baseline clinical parameters: a) Full blood count (minimum Hb, WCC, platelets); b) Biochemical series (creatinine, Na^+^, K^+^, bicarbonate, calcium, CRP, lipid profile, glucose, HbA1C); c) MDRD eGFR on latest creatinine; d) current calcineurin inhibitor 12 hour trough levels (as appropriate); e) Total immunoglobulin or IgG, IgM +/- IgA levels. If the participant has had blood taken for any of these tests, sampled for routine care up to one week prior to consent, use results present in hospital notes. If this is the case, then only take blood for trial-specific tests.

ii) Scientific analyses: 50-60 mls blood for separation of PBMC and 20mls for serum storage. For participants who require screening for HIV or hepatitis B/C for eligibility, baseline blood samples for scientific analyses will not be taken. Scientific blood samples from these participants will be taken on their next research clinic visit after eligibility has been confirmed. The collection of scientific blood samples will continue throughout the trial only as long as the resources and the capability of receiving, processing and storing samples are available.

iii) Analysis of HLA Ab status (10mls clotted blood), as described above, which will allow randomization to proceed.

All patients will be asked to complete questionnaires.

Subsequent intensive follow up visits.

HLA Ab+ participants in the unblinded group will have a discussion about the risks and prognostic significance of being antibody positive. The optimised treatment protocol will be introduced ideally within 3 months or as soon as possible thereafter in those HLA Ab+ patients allocated to this group (optimisation must be performed within 8 months of HLA Ab positive screening). The first optimisation visit can be performed by the physician either in the clinic or alternatively over the phone. Recruits will then be seen *up to* two weekly during this period (maximum of 6 extra clinic appointments are envisaged), though they should be on maintenance dose prednisolone 7 weeks after initiating optimisation. During this period they will have FBC (as above), creatinine, Na^+^, K^+^, glucose, calcineurin inhibitor trough levels and blood pressure monitored according to the schedule in 2.2.1. Optimisation visits will not be recorded in the eCRF but will be documented in an “Optimisation Log” at each site. Results from blood tests taken during the optimisation process will be recorded in the patients’ medical notes but not in the eCRF. Once stabilized, they will be seen at least 8 monthly in transplant clinic. Patients allocated to all other groups will be seen at least every 8 months in transplant clinic for formal study assessments. Trial-specific nurses will carry out all trial-related procedures with participants at follow up visits. Patients may be seen at other times during this period, according to clinical need, but study assessments should be done within the time parameters in Table 2.2.1. Ideally assessment times will be performed +/- 1 month of the study assessment month. However assessments can be performed up to 3 months prior and 3 months after the scheduled study visit without deviation to the protocol. If required, research nurses or clinic staff will contact participants up to a week before their allocated clinic appointment to ensure that the participant will be attending their appointment.

Once every 8 months the following will be recorded. a) Weight and bp; b) Full blood count (minimum Hb, WCC, platelets); c) Biochemical series (creatinine, Na^+^, K^+^, bicarbonate, calcium, CRP, glucose, HbA1C); d) MDRD eGFR on latest creatinine; e) calcineurin inhibitor trough levels; f) protein: creatinine ratio on urine sample; g) episodes of infection, malignancy or new DM; h) episodes considered to be adverse events; i) medications and doses participant is currently taking on the day of the follow up visit. We will not record medications taken for short courses during the 8 months prior to any study visit. Every 16 months total immunoglobulin or IgG, IgM +/- IgA levels will be measured and recorded in the eCRF. In addition, every 16 months a lipid profile will be performed. If research nurses are contacting participants by telephone to remind them of allocated appointments, and provided that the participant is in agreement, details regarding episodes of infection, malignancy or new DM and episodes considered to be adverse events will be collected during the phone call. These details will be updated on the appointment day and then recorded in the eCRF.

Assuming resources are available, on all patients with HLA Ab, and a small cohort of patients without, separate blood samples will be taken for separation and storage for future non-routine scientific analyses. Steps will be taken to ensure this sampling does not break the blinding of group allocations. Once every 8 months, HLA Ab-negative patients will undergo further screening for HLA Ab (see above). The aim of the study is to conduct all visit procedures, baseline and follow up, but as it is a Type A trial any procedure missing will not be considered a protocol deviation.

Upon completion of the 32- to 64-month intensive follow up, consisting of 8-monthly research visits, the participants will be told by the research nurses that they no longer need to attend to clinic for research visits but their regular clinic visits will continue. At the last intensive follow up visit a HLA Ab sample will be collected from all participants. Due to the changes required due to coronavirus pandemic described above (Section 4.6), data regarding the primary endpoint will be collected from patient notes for all participants. These data will be collected from the participants’ last hospital contact prior to March 16 2020 (pre-COVID pandemic). A sensitivity analysis will also be performed by collecting data a second time from the participant’s most recent hospital contact in the assessment period between September 1 2020 and November 30 2020, at which point participants’ involvement in the study will be completed.

Recruits consented to the trial prior to Protocol Version 12 (01/12/2016) will be re-consented specifically to access their patient notes to assess primary endpoint data after completion of their intensive follow-up visits. This re-consenting process will be performed by research nurses during a research clinic visit prior to the completion of the intensive follow-up.

In recruits with living donors (known to them), the living donors will be invited to participate either by the recruit, or directly by the study team, following consent from the recruit to inform donors of their participation in the study. Donors will attend the clinic at their convenience, where consent will be taken. If donors are no longer being followed up at the hospital involved in the trial, they will be contacted by telephone to discuss the study and arrange a mutually convenient time for them to attend. As these participants are only needed to provide blood, consent will not need to be taken by a doctor. Donors who have not been screened for HIV, HepBSAg or HepC within the last 5 years will need to have additional screening tests for these viruses. Finally, blood (up to 80mls) will be taken for separation of PBMC which will be stored in the research laboratory, identified only as the donor of a particular study recruit. Donors may be asked to donate another aliquot of blood at another time within the next three years. The collection of living donor blood samples will continue throughout the trial as long as the resources and the capability of receiving, processing and storing samples are available.

**5.2 Laboratory Tests**

**5.2.1 HLA Ab analysis:**

Serum prepared from 10mls of blood will be used in the commercially available ‘LABScreen’ tests, containing fluorescently tagged beads coated with purified HLA antigens. All participating centres have ‘Luminex’ equipment for analysis of these tests and the skills to process samples and interpret results. Therefore, the tests will be performed in each of the centres. A sequential analysis of samples is planned, first to identify those with HLA Ab, using mixed class I & class II Ab ‘screening’ beads coated with multiple different types of HLA; To interpret these tests, the manufacturer’s definition of a positive and negative test will be used. In those patients with positive results, the specificity of the HLA Ab will be determined by single antigen beads (SAB), coated with single HLA class I or class II antigens. As before, to interpret these tests, the manufacturer’s definition of a positive and negative test will be used.  Any patient with a positive test for HLA Ab identified by SAB will be regarded as HLA Ab+ positive for the trial if the mean fluorescence intensity (MFI) is ≥2000. If that HLA Ab is directed against a mismatched donor HLA antigen, this will be assigned as DSA+. The number of DSA with an MFI ≥2000 will be recorded to define the Ab 'burden' of an individual patient. In the final analysis, correlations between HLA Ab burden and outcomes will be sought. Patients with SAB positivity that is difficult to label as DSA/non DSA (because of insufficient data on donor mismatches, for instance), will be regarded as having non-DSA. Patient’s with a positive reaction on screening but lacking reactivity with the SAB at the level described will be considered negative. Excess serum will be stored. The same screening will continue on the HLA Ab negative patients every 8 months, with the samples taken at a routine clinic appointment. Patients with HLA Ab allocated to the unblinded arm will be told the result (possibly be telephone) as soon as possible and invited to undergo optimisation. Those in the blinded groups or in the unblinded HLA Ab negative arm will be told the result of their randomisation at their next clinic visit. All participants will be screened for HLA Abs at their last intensive follow up visit.

**5.2.2 Routine biochemical, haematological and calcineurin inhibitor trough analysis:**

These will be performed by the local clinical laboratories and results recorded as above.

**5.2.3 Scientific laboratory analysis:**

Serum and PBMC samples for future scientific analysis will be collected as long as resources allow; the precise nature of the analyses will be determined in future. Patients will also consent to allow analysis, for research purposes, of any stored serum, blood or tissues (such as transplant biopsies). This will apply for all existing and future samples taken for clinical reasons. In the case of future transplant biopsies, the patients will be asked to consent to having an extra core taken for transcriptome analysis. Subject to available funding, this will be stored in appropriate medium and transported to CI’s laboratory for storage.

**6 Assessment of Efficacy**

**6.1.1 Primary Efficacy Parameters**

• Time to graft failure assessed by remote evaluation when approximately 43 months post-randomisation is achieved by all.

**6.1.2 Secondary Efficacy Parameters**

• Graft dysfunction, as assessed by two separate measures, presence of proteinuria (Protein:Creatinine Ratio > 50 or Albumin:Creatinine Ratio > 35) at 32 months and change in estimated Glomerular Filtration Rates over 32 months.

• Rates of acute rejection over 32 months.

• Health economic analysis of outcomes in intervention vs. control groups.

• Analysis of adherence and perceptions of risk in all biomarker led care groups.

**6.2 Procedures for Assessing Efficacy Parameters**

• Graft failure; will be defined as the return to long-term dialysis or re-transplantation. This will be measured from the date of recruitment and will be reported for failure due to all causes. The date of re-starting dialysis or of re-transplantation will be recorded on the CRF.

• Estimated Glomerular Filtration rates (eGFRs) will be calculated using the Modification of Diet in Renal Disease (MDRD) Study equation and recorded on the CRF. Mean eGFR slopes at 32 months will be compared between arms, using all available observations between baseline and 32 months using a test of interaction in a linear mixed model.

• Proteinuria; This will be defined by a Protein:Creatinine Ratio (PCR) > 50 or an Albumin:Creatinine Ratio (ACR) > 35 from a urine sample. The PCR or ACR will be recorded on the CRF.

• Acute rejection; will be defined by a combination of: a) acute rise in serum creatinine prompting a renal biopsy; b) any pathology on the biopsy which meets criteria for acute rejection, according to latest BANFF criteria. The number of biopsies and the appropriate biopsy reports will be recorded on the CRF.

• Health economic analysis; Effectiveness: A full economic evaluation will adopt a NHS perspective. 16 month outcomes rates of; (1) graft failure; (2) patient survival (3) graft dysfunction (see defn. above); (4) acute rejection; (5) culture-positive infection, malignancy or diabetes. (6) EQ-5D, which is a patient-specific quality-adjusted life years (QALY) measurement. Cost-effectiveness: will use (1)-(5) where (1) is primary and the others are secondary outcomes. Cost-utility: will use (6) (EQ-5D questionnaires). Cost-benefit: Net benefit per patient calculated by multiplying QALY by assumed maximum willingness-to-pay for QALY (£20000 per QALY) and subtracting costs. Costs: of all interventions will be obtained from Guy’s or estimated by identifying relevant categories of resource utilization OR measuring the volume of each category and multiplying by the average NHS resource costs ([[30](#_ENREF_30)] BNF and NHS reference lists). Costs of intervention include the cost of screening beads and enhanced drug costs.

Measurement of adherence; Participants report of adherence behaviour will be assessed using the Medicines Adherence Report Scale (MARS) [[31](#_ENREF_31), [32](#_ENREF_32)], a valid and reliable scale that has been previously used to assess adherence in renal transplant recipients [[33](#_ENREF_33), [34](#_ENREF_34)]. Self-report measures have the advantage of being inexpensive and non-intrusive. However, it is known that self-report underestimates the true extent of nonadherence because of inherent self-presentational and recall biases. Self-presentational bias occurs when respondents may be reluctant to admit to nonadherence because they perceive a social contract where the expectation is one of high adherence. The MARS takes steps to diminish this bias by sanctioning and normalising reports of nonadherence. However, this does not totally remove the effect self-presentational and recall biases that are inherent in all self-report measures. For this reason we will apply a combined approach to adherence assessment, where initial categorisation of patients into high vs. low on the basis of self report is revised based on calcineurin inhibitor (CNI) blood monitoring (carried out in routine management for patients prescribed tacrolimus or ciclosporin) and tablet counts (conducted on a sample or participants). In this approach, reports of low adherence are accepted as self presentational biases act in the opposite direction (reports of low adherence are more reliable than reports of high adherence [[35](#_ENREF_35), [36](#_ENREF_36)]). Patients who report high adherence are reclassified to low adherence on the basis of CNI results (e.g if levels are undetectable then participant is assumed to be nonadherent) or tablet counts (e.g. if there is a greater than 20% discrepancy between the actual and expected tablet count then participant is reclassified as non adherent).

In order to explore the potential antecedents to participants’ adherence behaviours, they will be also be asked to complete specially adapted versions of questionnaires relating to

treatment intrusiveness (TIQ), symptoms associated with immunosuppressants (SAQ), beliefs about medicines (BMQ) [[37](#_ENREF_37)], satisfaction with information about their medicines (SIMS) [[38](#_ENREF_38)] and whether they are feeling anxious and/or depressed (HADS) [[39](#_ENREF_39)].  Perceived risk will be measured on the basis of questionnaire-based approaches to qualifying perceptions of personal risk (IPQr) [[40](#_ENREF_40)].

On the basis of their survey responses, a small number of participants will be purposively selected (e.g. positive and negative attitudes, high and low adherers) for qualitative interview to explore their perception of risk and adherence behaviours in more depth.

All patients taking part in the trial will be asked to complete all or some of the questionnaires at specified times (see table 2.2.1). Questionnaires will be administered electronically. Respondents will complete the survey online whilst in clinic, using an IPad or equivalent tablet device that is designated solely for this trial. Completed survey responses are stored on the Qualtrics secure servers and can only be accessed using a login/ password. Nothing will be recorded on the main trial CRF.

The questionnaires will be piloted in the first few participants recruited to the Guy’s site. These respondents will be asked to undergo cognitive interviewing whilst completing the survey, a technique used to ensure the validity of questionnaire items [[41](#_ENREF_41)]. On the basis of this pilot, the questionnaire items may undergo minor modification. The ease of utility of the online survey and tablet device will also be evaluated during the pilot.

**7. Assessment of Safety**

**7.1 Specification, Timing and Recording of Safety Parameters.**

The following safety parameters will be assessed as formal end-points for the trial:

• Patient survival.

• Rates of culture- or PCR-positive infection, biopsy-proven malignancy and diabetes mellitus. – these will be assessed at each formal study visit.

• Patient survival will be measured from day of recruitment. All deaths will be recorded, along with the cause and date, on the CRF.

• Infection; This will be defined as a positive microbiological culture or other test (such as PCR) confirming viral, bacterial or fungal replication in association with specific symptoms. Also, clinical episodes with classical presentations and signs (such as ‘shingles’ due to Herpes zoster) or episodes with confirmatory imaging of infection (for instance, consolidation on lung imaging) will be regarded as an infective episode and recorded.

• Malignancy; this will be defined by histopathological confirmation of malignancy on a biopsy of any suspicious lesion. The site of malignancy and the biopsy report (where available) will be filled in the CRF.

• Diabetes Mellitus; potential new cases of diabetes mellitus will be identified by elevated serum glucose and HbA1C measurements at study assessments and where possible, glucose measurements in between will be recorded on CRF. Standard WHO definitions for diagnosis of DM will be used to confirm diagnosis.

**7.2 Procedures for Recording and Reporting Adverse Events**

Recording of adverse events in the eCRF for OuTSMART will use the following definitions of expectedness reported below which are based on those listed in the SmPC for each IMP.

**Adverse Event (AE):** Any untoward medical occurrence in a subject to whom an IMP has been administered including occurrences which are not necessarily caused by or related to that product.

**Adverse Reaction (AR):** Any untoward and unintended response in a subject to an IMP which is related to any dose administered to that subject.

**Unexpected Adverse Reaction (UAR):** An adverse reaction the nature and severity of which is not consistent with the information about the IMP in question set out in the summary of product characteristics (SmPC) for that product.

**Serious Adverse Event (SAE):** Serious Adverse Reaction (SAR) or Unexpected Serious Adverse Reaction (USAR): Any adverse event, adverse reaction or unexpected adverse reaction, respectively, that

Results in death;

Is life-threatening;

Required hospitalisation or prolongation of existing hospitalisation;

Results in persistent or significant disability or incapacity;

Consists of a congenital anomaly or birth defect.

This trial fulfils the criteria for a ‘Type A’ trial (i.e. risk no higher than that of standard care). Therefore, there will be reduced reporting of adverse events to the Sponsor and the MHRA. In this trial, SAE’s will be reported on only those patients in who, medication is assigned IMP status i.e. those in the unblinded HLA Ab positive arm who have undergone optimisation. In addition, only serious adverse events that fulfil the following criteria will be reported to the sponsor and MHRA:

1. result in death
2. require hospitalisations resulting in kidney graft failure
3. are SAR’s that would prompt yellow-card reporting in the blinded arm of the trial.

In the very unlikely event of pregnancy, study subjects will not be withdrawn from the treatment. The study IMP is used as per standard of care and treatment will be optimised specifically for the patient. Therefore the risk is not greater than that of standard of care. Thus, even though it is not a serious adverse event, any unplanned pregnancy in patients taking the IMP will be reported via the SAE reporting system as stated below.

We will not report important medical events (IME).

**7.3 Reporting Responsibilities**

King’s College London and GSTT have delegated the delivery of the Sponsor’s responsibility for Pharmacovigilance (as defined in Regulation 5 of the Medicines for Human Use (Clinical Trials) Regulations 2004 to the King’s Health Partner’s (KHP) Clinical Trials Office (CTO).

The PIs on each site will take responsibility for reporting all adverse events (and pregnancy) to the Chief Investigator. All SAEs, SARs and SUSARs (excepting those specified in this protocol as not requiring reporting – see above) will be reported by the local investigators on the SAE form provided by the KHP CTO to the Chief Investigator, *immediately they become aware,* and by the Chief Investigator to the KHP CTO in accordance with the current Pharmacovigilance Policy. SAEs will be reported up to the last intensive visit (i.e. either at 32-months or up to 64 months) of each recruit.

All deaths will be reported as SAEs. Those that occur as a result of disease progression and other events that are primary or secondary outcome measures will also be reported on the appropriate CRF.

Important Medical Events that may not be immediately life-threatening or result in death or hospitalisation but may jeopardise the patient or may require intervention to prevent one of the other outcomes listed in the SAE definition should also be considered serious. However, as stated, we will not report IMEs to the sponsor unless fulfilling the criteria for SAE reporting set out above.

The KHP CTO will report SUSARs to the regulatory authorities (MHRA, competent authorities of other EEA (European Economic Area) states in which the trial is taking place.

The Chief Investigator will report to the relevant ethics committee. Reporting timelines are as follows:

- SUSARs which are fatal or life-threatening must be reported not later than 7 days after the sponsor is first aware of the reaction. Any additional relevant information must be reported within a further 8 days.
- SUSARs that are not fatal or life-threatening must be reported within 15 days of the sponsor first becoming aware of the reaction.

The Chief Investigator and KHP CTO (on behalf of the *co*-sponsors), will submit a Development Safety Update Report (DSUR) relating to this trial’s IMPs, to the MHRA and REC annually.

**7.3.1 Adverse events that do not require reporting**

In the unblinded HLA Ab positive groups receiving IMPs:

• Events or reactions listed in the SmPC for each of the IMPs do not need reporting for this trial (see http://www.medicines.org.uk/emc), unless they are SAEs according to trial reporting guidelines above. A summary of AEs from the SmPCs are included in appendix 1.

• AE’s not thought to be related to the IMP must be recorded in the eCRF but do not need reporting, unless they are SAE’s according to trial reporting guidelines above.

In all other study (Non-IMP-receiving) groups:

• AEs and SAEs occurring in subjects not administered an IMP must be recorded in the eCRF but do not need reporting to the sponsor/MHRA.

• AR’s in these subjects should be reported using the standard yellow form system

In all groups:

• Hospital admissions occurring as a result of a planned or elective admission for any reason will not be regarded as SAE according to trial reporting guidelines above unless the site PI decides they need reporting, in which case the procedure above should be followed. All adverse events should be recorded throughout the trial, by the PIs within 28 days of becoming aware.

**7.4 Treatment Stopping Rules**

The trial will stop recruiting once 165 HLA Ab positive DSA patients have been recruited, including patients who are HLA Ab positive with DSA antibodies at baseline and those who become HLA Ab positive DSA at the 8-monthly rescreening rounds.

The trial may be prematurely discontinued by the Sponsor, Chief Investigator or Regulatory Authority on the basis of new safety information or for other reasons given by the Data Monitoring & Ethics Committee / Trial Steering Committee regulatory authority or ethics committee concerned.

The trial may also be prematurely discontinued due to lack of recruitment or upon advice from a Trial Steering Committee who will advise on whether to continue or discontinue the study and make a recommendation to the sponsor. If the study is prematurely discontinued, active participants will be informed and no further participant data will be collected

Earlier termination *will be considered* by the Data Monitoring committee if there is a significant excess of adverse events in the intervention arm.

**8. Statistics**

**8.1 Sample Size**

The primary purpose of this trial is to demonstrate superior outcomes using a defined treatment strategy in biomarker (HLA Ab) positive patients, and at the same time demonstrate non-inferior outcomes when the unblinded screening strategy is applied to the entire patient population. Time to graft failure has been chosen as a clinically relevant primary outcome. As a reference for power calculations, we have used the observed failure rates reported by Lachmann et al. [[8](#_ENREF_8)] for HLA Ab+ and HLA Ab-neg patients. Since failure rates differ between DSA+ and non-DSA+ patients, sample size calculations have been carried out separately for these groups. Following these calculations, we have estimated the number to be screened, based on expected drop out rates, expected screening results and eligibility criteria (see below).

We have based our estimates of the *differences* in primary outcome between groups on two things; first, the results of our preliminary data from patients with CR treated with a similar regime as used here; second, our assessment that large differences in primary outcome will be needed to make the screening programme cost-effective.

Hypotheses and power calculations: (group labels refer to flow diagram in section 2.3)

1. Superiority on Biomarker Positive Patients:
   1. A_1_>B_1_ : HLA Ab+ patients, with DSA, randomized to standard care (A_1_) will show higher graft failure rates than patients randomized to biomarker-led care (B_1_). We hypothesize that the experimental treatment will bring the failure rate in group B_1_ down to that of non-DSA patients in standard care (A_2_). Assuming that 30% of patients with DSA randomised to standard care (A_1_) will have experienced chronic rejection (CR) by 3-years follow up, we expect treatment optimisation to reduce the rate of CR in DSA patients randomised to group B_1_ down to 16% at 3-years follow up (rate observed in patients with non-DSA). This corresponds to a Hazard ratio (HR) of 0.489. The expectation is for 11% and 21% of CR among patients with DSA in in group A_1_ at 1 and 2-years follow up respectively (as in [[8](#_ENREF_8)]), and extrapolating based on a HR of .489, we expect BLC to reduce those CR to 5.5%, and 10.89% at 1 and 2-years. Using a variable follow up design assuming an average accrual monthly rate of 3.6 patients per month, and a follow up time of 43 months, recruiting 165 patients with DSA would allow us to observe 23/83 (28%) events of CR in patients under biomarker led care (B_1_), and 39/82 (47%) in the standard care group (A_1_). This would provide 80% power and 5% type I error, for a two-sided log-rank test.
   2. A_2_>B_2_ : HLA Ab+ patients, with non-DSA, randomized to standard care (A_2_) will show higher graft failure rate than patients randomized to biomarker-led care (B_2_). We hypothesize that the experimental treatment will bring the failure rate in group B_2_ down to that of biomarker negative patients in standard care (C). Assuming that 16% of patients with NDSA randomised to SoC will have experienced chronic rejection (CR) by 3-years follow up, we expect treatment optimisation to reduce the rate of CR in NDSA patients randomised to BLC down to 6% at 3-years follow up (rate observed in patients without HLA antibodies). This corresponds to a Hazard ratio of 0.351. Based on Lachman et al. the expectation is for 3% and 11% of CR among patients with NDSA in SoC at 1 and 2-years follow up respectively, and extrapolating based on a HR of 0.351, we expect BLC to reduce those CR to 1.1%, and 4.1% at 1 and 2-years. Using a variable follow-up design (patients followed until failure, drop out or end of minimum follow up), assuming an average accrual monthly rate of 15.5 patients per month, and a minimum follow up time of 22.4 months, recruiting 296 patients with NDSA, would allow us to observe 8/149 (5.3%) events of CR in patients under BLC, and 21/147 (14%) in the SoC group (total duration = 41.5 months). This would provide 80% power to determine a statistically significant difference between SoC and BLC, using a log-rank test, with a 2-sided type-I error rate.

The numbers enrolled in groups A & B include those patients initially enrolled in groups C or D who become HLA Ab+ during re-screening.

1. Non-inferiority of all Unblinded patients compared to all Blinded patients:
   1. A_1_+A_2_+C ≥ B_1_+B_2_+D : All patients randomized to unblinded screening will show equal or lower graft failure rates than all patients randomized to blinded screening, irrespective of biomarker status. At the end of the trial, we expect 58% of patients to be in the HLA Ab negative groups, 7% DSA+ groups and 35% non-DSA+ groups (after drop-outs). At the time of planning the OuTSMART study, we calculated that based on all assumptions above, all patients randomised to SoC combined would experience 13.9% of CR. We established a non-inferiority limit of 5% absolute difference in rate of CR at 3-years, so that the BLC group would be considered inferior to SoC with a CR rate of 18.9% or higher (expectation under the null hypothesis). This corresponds to a HR of 1.4 under the null hypothesis, and a HR of 0.63 under the alternative. Recruiting 672 patients over a period of 13.2 months, at an average accrual rate of 51 patients per month, and a minimum follow up of 18.21 months, would allow us to observe 22/337 (6.5%) events of CR in the SoC group, and 32/335 (9.5%) in the BLC group. This would provide 90% power to demonstrate non-inferiority with a one-sided 95% Confidence Interval of the HR estimated using a Cox regression model. Given the above proportions, this requires enrolling 336 patients in each of groups C&D and this should allow 423 total patients to reach the primary endpoint (i.e. remain negative (after dropouts) at the end of their three year follow-up).

An audit of potentially available patients within the 5 renal units was performed initially to determine the likelihood of the study being able to recruit all the required patients from the 5 centres. We considered the number of patients in each centre under annual follow-up and the numbers of new patients who will become eligible throughout the first three years of the study (i.e. those who reach >12 months post transplantation, which are those transplanted in the period between12 months prior to the study and the end of year two). We estimated that 60% of these will be potential recruits, the others having reasons for not being included. We assumed that 10% of those approached will refuse consent and 10% of HLA Ab+ patients would have no detectable single Class I or II on single bead analysis. Additionally, we expected 6% of initially Ab-neg patients to become Ab+ in each screening round.

Following 16 months of recruitment to the OuTSMART trial, the observed % of DSA patients (including those from re-screening rounds) was lower than expected, at 6.6%. The percentage of antibody positive patients at baseline was 35.1%, considerably higher than expected (25-30%). 5.8% of all patients had DSA at baseline (expected 9%). 300 Ab-neg patients had been re-screened as part of the Month-8 screening round, of whom 23 had developed de-novo antibodies (7.6% - expected 6%). Five out of the 23 had DSA (1.6% of all –expected 2%).

Based on an overall expected proportion of 7% DSA participants (including from re-screening rounds) we will need to recruit 2357 patients overall to recruit the target of 165 DSA patients. Because of this requirement to recruit sufficient DSA participants, the recruits to the other groups are likely to be more than the minimum required for statistical power for the individual hypotheses.

**8.2 Randomisation**

Using the flow diagram (section 2.3, top-to-bottom) as a guide: renal transplant recipients aged 18-75, > 1 year post-transplant with an eGFR ≥ 30 will consent to the screening/treatment process. The first stratification will result from blood test screening for HLA Ab. Approximately 35% will be HLA positive, with ~65% negative. The HLA Ab+ patients will be further screened with single antigen beads to determine whether DSA are present (~1/6 DSA and 5/6 non-DSA). Thus, biomarker stratification leads to three groups (DSA+, non-DSA+ and HLA Ab-neg). The second stratification will be based on current immunosuppression, to ensure balanced numbers already on Tac or MMF in each group. The final stratification will be by site.

HLA Ab+ patients will be randomized 1:1 into either Blinded Standard Care or Unblinded Biomarker led-care. Patients in the former (groups A1 &A2) will be blind to their biomarker status and will remain on baseline immunotherapy, whereas patients in the latter (groups B1 and B2) will know their HLA Ab status and will be offered “treatment”. HLA Ab- patients will be randomized 1:1 into either Blinded (group C) or Unblinded (group D) and remain on standard care, with only the latter knowing their HLA Ab status. All HLA Ab-negative patients (groups C & D) will receive regular (8 monthly) Ab status monitoring for the first 3 years. Those patients who become positive during subsequent screening rounds (~10% per year) will be moved to the appropriate HLA Ab positive groups (DSA+ or non-DSA+) for final data analysis. For details of the randomisation procedure see section 4.4.

**8.3 Analysis**

Statistical analysis will be on an intention to treat and treatment received-basis, to consider the patients who become positive during the follow up in the appropriate group (group labels refer to flow diagram in section 2.3).

The primary analysis will use data collected up until March 16 2020 and analyses will be conducted for each of the hypotheses as outlined below. A sensitivity analysis will be carried out for the primary outcome using, additionally, data from participants’ most recent hospital contact as of the assessment period between September 1 2020 and November 30 2020. The sensitivity analysis will otherwise be carried out in exactly the same way for each of the hypotheses.

1. Superiority:

H_0_: h_A1_(t)= h_B1_(t) & h_A2_(t)= h_B2_(t)^^[[14]](#footnote-14)^^

H_1_: h_A1_(t)≠ h_B1_(t) & h_A2_(t)≠ h_B2_(t)

In order to test superiority for the primary outcome in the Biomarker (HLA Ab) positive groups (Hypothesis 1.1 and 1.2), we will use Cox proportional hazards regression models to estimate the graft failure hazard ratio between the biomarker led care and standard care groups and test at the 5% level of significance. Results will be given as estimates and 95% confidence intervals (CIs). Within the model, we will adjust for previous immunosuppression regimen and research site (as these are the randomisation stratification factors) for increased statistical efficiency.

We will check the proportional hazards assumption by examining Kaplan-Meier plots and by testing for an interaction between group (BLC or SC) and time to graft failure within the model.

1. . Non-inferiority:

H_0_: h_Unblind_(t) / h_Blind_(t) ≥ δ

H_1_: h_Unblind_(t) / h_Blind_(t) < δ

In order to test for non-inferiority of the unblinded groups compared to the blinded groups (hypothesis 2.1), we will use Cox proportional hazards regression models to estimate the graft failure hazard ratio. We will adjust for the stratification factors in the model as outlined above and check the proportional hazards assumption by examining Kaplan Meier plots and by testing for an interaction between unblinded/blinded group and time to graft failure. We will conclude non-inferiority if H_0_ gets rejected at 5% significance, and the corresponding upper bound of the 95% CI for the hazard ratio excludes the limit δ (hazard ratio of 1.4).

We will use a similar procedure using Cox proportional hazards regression for the analysis of secondary time to event (survival outcomes). Where numbers allow, secondary binary outcomes will be analysed using logistic regression with adjustment for stratification factors. Where numbers are too small for this, the z-test or Fisher’s exact will be used. Results will be given as estimates (odds ratios or differences in proportions) and 95% CIs. For continuous secondary outcomes we will use linear regression (or a linear mixed model if accounting for repeated measures) with adjustment for stratification factors, transforming data where they are skew.

Economic Evaluation: Cost data is usually skew but will be analysed using arithmetic means so that total costs are preserved. Non-normality in errors will be allowed for by using generalized linear models with appropriate error structure (e.g. gamma distribution[[42](#_ENREF_42)]). Incremental cost-effectiveness ratios (ICERs) or incremental cost-utility ratio (ICUR) will be presented where appropriate. Cost effectiveness acceptability curves will be plotted to summarize information on uncertainty in cost-effectiveness.

**9. Trial Steering Committee**

An independent Trial Steering Committee (TSC) will be convened in the post-award period. The membership will be decided by the CI and approved by the NIHR. The chair will be a senior transplantation physician or surgeon from the UK who is unconnected to the study. Members will include the CI, two other PIs from the trial, a representative of the GSTT Kidney Patients Association, one other senior independent renal/transplant physician/surgeon, and an independent senior HLA clinical scientist.

The TSC will meet at least annually during the study, approximately 2 weeks after the DMC. The TSC is an executive committee. Terms of reference of the TSC will be agreed and documented prior to start of recruitment. The Trial Manager will prepare reports to the TSC

**10. Data Monitoring Committee**

A Data Monitoring and Ethics Committee (DMC) will be established comprising a senior UK-based transplant physician/surgeon as chair, an HLA clinical scientist, a biostatistician and trials-experienced pharmacist. All the members will be independent of the trial.

The DMC will meet at least annually during the study, approximately 2 weeks prior to the TSC. The DMC is advisory to the TSC. The DMC charter will be drafted and agreed prior to recruitment. The Trial Statistician will prepare reports to the DMC.

**11. Direct Access to Source Data and Documents**

The investigators and the institutions will permit trial-related monitoring, audits, REC review, and regulatory inspections (where appropriate) by providing direct access to source data and other relevant documents.

**12. Ethics & Regulatory Approvals**

The trial will be conducted in compliance with the principles of the Declaration of Helsinki (1996), the principles of GCP and in accordance with all applicable regulatory requirements including but not limited to the Research Governance Framework and the Medicines for Human Use (Clinical Trial) Regulations 2004, as amended in 2006 and any subsequent amendments.

This protocol and related documents will be submitted for review to London-Hampstead Research Ethics Committee (REC), and to the Medicines and Healthcare products Regulatory Agency (MHRA) for Clinical Trial Authorisation

The Chief Investigator will submit a final report at conclusion of the trial to the KHP CTO (on behalf of the Sponsor), the REC and the MHRA within the timelines defined in the Regulations.

**13. Quality Assurance**

Monitoring of this study to ensure compliance with Good Clinical Practice and scientific integrity will be managed and oversight retained by KHP CTO. All samples will be anonymised before laboratory analysis. No patient-related data will be held in research laboratories. During the study, paper copies will be held in a locked filing cabinet in the chief investigators office and retained for a minimum of 5 years following the end of the study.

The investigators and the institutions will permit trial-related monitoring, audits, REC review, and regulatory inspections (where appropriate) by providing direct access to source data and other relevant documents (ie patients’ case sheets, blood test reports, X-ray reports, histology reports etc).

All study data will be stored and archived in line with the Medicines for Human Use (Clinical Trials) Amended Regulations 2006 as defined in the Clinical Trials Office Archiving SOP. Record keeping will be the responsibility of the investigators.

The chief investigator will review all presentations and publications arising from this study and decide authorship in accordance with accepted guidelines.

**14. Data Handling, Publication Policy and Finance**

The Chief Investigator will act as custodian for the trial data. The following guidelines will be strictly adhered to:

Patient data will be anonymised

- All anonymised data will be stored on a password protected computers.
- All trial data will be stored in line with the Medicines for Human Use (Clinical Trials) Amended Regulations 2006 and the Data Protection Act.

and archived in line with the Medicines for Human Use (Clinical Trials) Amended Regulations 2006 as defined in the Clinical Trials Office Archiving SOP.

**15. Data Handling**

Research data will be collected at sites onto source data worksheets, which will form part of the NHS medical notes. Clinical and research data will be transcribed from the medical notes and source data worksheets to the study eCRF system, hosted at the King’s Clinical Trials Unit, KCL. The eCRF (InferMed MACRO) is GCP and FDA 21 CFR Part 11 compliant with e-signatures for site PI confirmation of each eCRF at end of study. Data entry staff at site will be provided with unique usernames and passwords to the system and will be trained in data entry by the trial manager. Study monitors will be given access to review data on the system, raise discrepancies and confirm source data verification checks. The study trial manager and data manager will have access to review data on the system and raise discrepancies. All requests for access to the data entry system must be authorised by the trial manager. All requests for data exports must be authorised by the trial statistician.

**16. Publication Policy**

It is intended that the results of the study will be reported and disseminated at international conferences and in peer-reviewed scientific journals.

**17. Insurance / Indemnity**

The study will be indemnified by King’s College London for negligent and non-negligent harm. In addition, the Chief Investigator and local Principal Investigators (the clinicians) also have independent insurance with medical defence societies.

**18. Financial Aspects**

The NIHR have supported the study through an EME programme grant award. The scientific analyses of stored blood samples will be funded separately. The analysis of adherence and perceived risk will be funded separately via a PhD fellowship application by Professor Rob Horne.

**19. Signatures**

 08/07/2020

______________________________________ _________________________

Chief Investigator Date

Print name

**Appendix 1**

**Adverse drug reactions mentioned in the SmPCs for the IMPS. These combine pre- and post-marketing experience. These do not require reporting as adverse events in OuTSMART unless resulting in an SAE.**

**MMF**

*Infections and infestations:^^[[15]](#footnote-15)^^*

Very common Sepsis, gastrointestinal candidiasis, urinary tract infection, herpes simplex, herpes zoster

Common Pneumonia, influenza, respiratory tract infection, respiratory moniliasis, gastrointestinal infection, candidiasis, gastroenteritis, infection, bronchitis, pharyngitis, sinusitis, fungal skin infection, skin candida, vaginal candidiasis, rhinitis

The most serious infections including meningitis, endocarditis, tuberculosis and

atypical mycobacterial infection. Cases of BK virus associated nephropathy, as well as cases of JC virus associated progressive multifocal leukoencephalopathy (PML), have been reported in patients treated with immunosuppressants, including MMF.

*Neoplasms:^^[[16]](#footnote-16)^^*

Common Skin cancer, benign neoplasm of skin

*Blood and lymphatic system disorders:*

Very common Leukopenia, thrombocytopenia, anaemia

Common Pancytopenia, leukocytosis

Agranulocytosis and neutropenia have been reported; therefore, regular monitoring of patients taking MMF is advised^^[[17]](#footnote-17)^^. There have been reports of aplastic anaemia and bone marrow depression in patients treated with MMF, some of which have been fatal.

Cases of pure red cell aplasia (PRCA) have been reported in patients treated with MMF. Isolated cases of abnormal neutrophil morphology, including the acquired Pelger-Huet anomaly, have been observed in patients treated with MMF. These changes are not associated with impaired neutrophil function. These changes may suggest a 'left shift' in the maturity of neutrophils in haematological investigations, which may be mistakenly interpreted as a sign of infection in immunosuppressed patients such as those that receive MMF.

Uncommon Pseudolymphoma and bone marrow failure.

*Metabolism and nutrition disorders:*

Common Acidosis, hyperkalaemia, hypokalaemia, hyperglycaemia, hypomagnesaemia, hypocalcaemia, hypercholesterolaemia, hyperlipidaemia, hypophosphataemia, hyperuricaemia, gout, anorexia

*Psychiatric disorders:*

Common Agitation, confusional state, depression, anxiety, thinking abnormal, insomnia

*Nervous system disorders:*

Common Convulsion, hypertonia, tremor, somnolence, myasthenic syndrome, dizziness, headache, paraesthesia, dysgeusia

*Cardiac disorders:*

Common Tachycardia

*Vascular disorders:*

Common Hypotension, hypertension, vasodilatation, venous thrombosis

Uncommon Lymphocele

*Respiratory, thoracic and mediastinal disorders:*

Common Pleural effusion, dyspnoea, cough

There have been isolated reports of interstitial lung disease and pulmonary fibrosis in patients treated with MMF in combination with other immunosuppressants, some of which have been fatal. There have also been report of bronchiectasis in children and adults.

*Gastrointestinal disorders:*

Very common Vomiting, abdominal pain, diarrhoea, nausea, constipation, dyspepsia

Common Gastrointestinal haemorrhage, peritonitis, ileus, colitis, gastric ulcer, duodenal ulcer, gastritis, oesophagitis, stomatitis, flatulence, eructation, gingival hyperplasia, colitis including cytomegalovirus colitis^^[[18]](#footnote-18)^^, pancreatitis and intestinal villous atrophy.

*Hepatobiliary disorders:*

Common Hepatitis, jaundice, hyperbilirubinaemia

*Skin and subcutaneous tissue disorders:*

Common Skin hypertrophy, rash, acne, alopecia,

*Musculoskeletal and connective tissue disorders:*

Common Arthralgia

*Renal and urinary disorders:*

Common Renal impairment

*General disorders and administration site conditions:*

Oedema, including peripheral, face and scrotal oedema, was reported very commonly during the pivotal trials. Musculoskeletal pain such as myalgia, and neck and back pain were also very commonly reported.

*Immune system disorders*

Hypogammaglobulinaemia has been reported in patients receiving CellCept in combination with other immunosuppressants.

*Investigations:*

Common Hepatic enzyme increased, blood creatinine increased, blood lactate dehydrogenase increased, blood urea increased, blood alkaline phosphatase increased, weight decreased

*Hypersensitivity:^^[[19]](#footnote-19)^^*

Hypersensitivity reactions, including angioneurotic oedema and anaphylactic reaction have been reported.

**Tacrolimus**

*Infections and infestations:^^[[20]](#footnote-20)^^*

As is well known for other potent immunosuppressive agents, patients receiving tacrolimus are frequently at increased risk for infections (viral, bacterial, fungal, protozoal). The course of pre-existing infections may be aggravated. Both generalised and localised infections can occur. Cases of BK virus associated nephropathy, as well as cases of JC virus associated progressive multifocal leukoencephalopathy (PML), have been reported in patients treated with immunosuppressants, including tacrolimus.

*Neoplasms:^^[[21]](#footnote-21)^^*

Patients receiving immunosuppressive therapy are at increased risk of developing malignancies. Benign as well as malignant neoplasms including EBV-associated lymphoproliferative disorders and skin malignancies have been reported in association with tacrolimus treatment.

*Blood and lymphatic system disorders:*

Common: anaemia, leukopenia, thrombocytopenia, leukocytosis, red blood cell analyses abnormal

Uncommon: coagulopathies, coagulation and bleeding analyses abnormal, pancytopenia, neutropenia

Rare: thrombotic thrombocytopenic purpura, hypo-prothrombinaemia, thrombotic microangiopathy

Not known: pure red cell aplasia, agranulocytosis, haemolytic anaemia.

*Immune system disorders:^^[[22]](#footnote-22)^^*

Allergic and anaphylactoid reactions have been observed in patients receiving tacrolimus

*Endocrine disorders:*

Rare: hirsuitism

*Metabolism and nutrition disorders:*

Very common: hyperglycaemic conditions, diabetes mellitus^^[[23]](#footnote-23)^^, hyperkalaemia

Common: hypomagnesaemia, hypophosphataemia, hypokalaemia, hypocalcaemia, hyponatraemia, fluid overload, hyperuricaemia, appetite decreased, anorexia, metabolic acidoses, hyperlipidaemia, hypercholesterolaemia, hypertriglyceridaemia, other electrolyte abnormalities

Uncommon: dehydration, hypoproteinaemia, hyperphosphataemia, hypoglycaemia

*Psychiatric disorders:*

Very common: insomnia

Common: anxiety symptoms, confusion and disorientation, depression, depressed mood, mood disorders and disturbances, nightmare, hallucination, mental disorders

Uncommon: psychotic disorder

*Nervous system disorders:*

Very common: tremor, headache

Common: seizures, disturbances in consciousness, paraesthesias and dysaesthesias, peripheral neuropathies, dizziness, writing impaired, nervous system disorders

Uncommon: coma, central nervous system haemorrhages and cerebrovascular accidents, paralysis and paresis, encephalopathy, speech and language abnormalities, amnesia

Rare: hypertonia

Very rare: myasthenia

*Eye disorders:*

Common: vision blurred, photophobia, eye disorders

Uncommon: cataract

Rare: blindness

Not known: optic neuropathy

*Ear and labyrinth disorders:*

Common: tinnitus

Uncommon: hypoacusis

Rare: deafness neurosensory

Very rare: hearing impaired

*Cardiac disorders:*

Common: ischaemic coronary artery disorders, tachycardia

Uncommon: ventricular arrhythmias and cardiac arrest, heart failures, cardiomyopathies, ventricular hypertrophy, supraventricular arrhythmias, palpitations, Rare: pericardial effusion

Very rare: *Torsades de Pointes*

*Vascular disorders:*

Very common: hypertension

Common: haemorrhage, thrombembolic and ischaemic events, peripheral vascular disorders, vascular hypotensive disorders

Uncommon: infarction, venous thrombosis deep limb, shock

*Respiratory, thoracic and mediastinal disorders:*

Common: dyspnoea, parenchymal lung disorders, pleural effusion, pharyngitis, cough, nasal congestion and inflammations

Uncommon: respiratory failures, respiratory tract disorders, asthma

Rare: acute respiratory distress syndrome

*Gastrointestinal disorders:*

Very common: diarrhoea, nausea

Common: gastrointestinal inflammatory conditions, gastrointestinal ulceration and perforation, gastrointestinal haemorrhages, stomatitis and ulceration, ascites, vomiting, gastrointestinal and abdominal pains, dyspeptic signs and symptoms, constipation, flatulence, bloating and distension, loose stools, gastrointestinal signs and symptoms

Uncommon: ileus paralytic, peritonitis, acute and chronic pancreatitis, blood amylase increased, gastrooesophageal reflux disease, impaired gastric emptying

Rare: subileus, pancreatic pseudocyst

*Hepatobiliary disorders:*

Common: cholestasis and jaundice, hepatocellular damage and hepatitis, cholangitis*^^[[24]](#footnote-24)^^*

Rare: hepatitic artery thrombosis, venoocclusive liver disease

Very rare: hepatic failure, bile duct stenosis

*Skin and subcutaneous tissue disorders:*

Common: pruritus, rash, alopecias, acne, sweating increased

Uncommon: dermatitis, photosensitivity

Rare: toxic epidermal necrolysis (Lyell's syndrome)

Very rare: Stevens Johnson syndrome

*Musculoskeletal and connective tissue disorders:*

Common: arthralgia, muscle spasms, pain in extremity, back pain

Uncommon: joint disorders

Rare: mobility decreased

*Renal and urinary disorders:*

Very common: renal impairment

Common: renal failure, renal failure acute, oliguria, renal tubular necrosis, nephropathy toxic, urinary abnormalities, bladder and urethral symptoms

Uncommon: anuria, haemolytic uraemic syndrome

Very rare: nephropathy, cystitis haemorrhagic

*Reproductive system and breast disorders:*

Uncommon: dysmenorrhoea and uterine bleeding

*General Disorders and administration site conditions:*

Common: asthenic conditions, febrile disorders, oedema, pain and discomfort, body temperature perception disturbed

Uncommon: multi-organ failure, influenza like illness, temperature intolerance, chest pressure sensation, feeling jittery, feeling abnormal,

Rare: thirst, fall, chest tightness, ulcer

Very rare: fat tissue increased

*Investigations:*

common: hepatic enzymes and function abnormalities, blood alkaline phosphatase increased, weight increased

uncommon: amylase increased, ECG investigations abnormal, heart rate and pulse investigations abnormal, weight decreased, blood lactate dehydrogenase increased

very rare: echocardiogram abnormal, electrocardiogram QT prolonged

*Description of selected adverse reactions:*

Pain in extremity has been described in a number of published case reports as part of Calcineurin-Inhibitor Induced Pain Syndrome (CIPS). This typically presents as a bilateral and symmetrical, severe, ascending pain in the lower extremities and may be associated with supra-therapeutic levels of tacrolimus. The syndrome may respond to tacrolimus dose reduction. In some cases, it was necessary to switch to alternative immunosuppression.

**Prednisolone**

*Infections and infestations:*

Increased susceptibility and severity of infections*^^[[25]](#footnote-25)^^* with suppression of clinical symptoms

and signs, opportunistic infections, recurrence of dormant tuberculosis.

*Blood and lymphatic system disorders:*

Leukocytosis

*Immune system disorders:*

Hypersensitivity including anaphylaxis, fatigue and malaise

*Endocrine disorders:*

Cushing syndrome, Cushingoid facies, weight gain, impaired carbohydrate tolerance with increased requirement for antidiabetic therapy, manifestation of latent diabetes mellitus, menstrual irregularity and amenorrhea.

*Metabolism and nutrition disorders:*

Sodium and water retention, hypokalaemic alkalosis, potassium loss, negative nitrogen and calcium balance.

*Psychiatric disorders:*

A wide range of psychiatric reactions including affective disorders (such as irritable, euphoric, depressed and labile mood, and suicidal thoughts), psychotic reactions (including mania, delusions, hallucinations, and aggravation of schizophrenia), marked euphoria leading to dependence; aggravation of epilepsy, behavioural disturbances, irritability, nervousness, anxiety, sleep disturbances, and cognitive dysfunction including confusion and amnesia have been reported. Reactions are common and may occur in both adults and children. In adults, the frequency of severe reactions has been estimated to be 5-6%. Psychological effects have been reported on withdrawal of corticosteroids; the frequency is unknown. Psychological dependence, dizziness, headache, vertigo.

*Eye disorders:*

Increased intra-ocular pressure, glaucoma, papilloedema, posterior subcapsular cataracts, central serous chorioretinopathy, exophthalmos, corneal or scleral

thinning, scleral perforation, exacerbation of ophthalmic viral or fungal disease and vision, blurred.

*Cardiac disorders:*

Congestive heart failure in susceptible patients, hypertension.

*Vascular disorders:*

Thromboembolism

*Gastrointestinal:*

Dyspepsia, nausea, peptic ulceration with perforation and hemorrhage, abdominal distension, abdominal pain, increased appetite, oesophageal candidiasis, oesophageal ulceration, acute pancreatitis, perforation of the small bowel, particularly in patients with inflammatory bowel disease.

*Skin and subcutaneous tissue disorders:*

Hirsutism, skin atrophy, bruising, impaired healing, striae, telangiectasia, acne, increased sweating, may suppress reactions to skin tests, pruritis, rash, urticaria

*Musculoskeletal and connective tissue disorders:*

Proximal myopathy, osteoporosis, vertebral and long bone fractures , avascular osteonecrosis, tendon rupture, myalgia, muscle weakness, wasting and loss of muscle mass.

*Renal and urinary disorders:*

Nocturia, scleroderma renal crisis (Amongst the different subpopulations the occurrence of scleroderma renal crisis varies. The highest risk has been reported in patients with diffuse systemic sclerosis. The lowest risk has been reported in patients with limited systemic sclerosis (2%) and juvenile onset systemic sclerosis (1%))

*General disorders and administration site conditions:*

Impaired healing and withdrawal symptoms

*Withdrawal symptoms:*

Too rapid a reduction of corticosteroid dosage following prolonged treatment can lead to acute adrenal insufficiency, hypotension and death. A “withdrawal syndrome” seemingly unrelated to adrenocortical insufficiency may also occur following abrupt discontinuance of glucocorticoids. This syndrome includes symptoms such as: anorexia, nausea, vomiting, lethargy, headache, fever, joint pain, desquamation, myalgia, arthralgia, rhinitis, conjunctivitis, painful itchy skin, weight loss and or hypotension. These effects are thought to be due to the sudden change in glucocorticoid concentration rather than too low corticosteroid levels.

*Additional side effects in children and adolescents:*

Suppression of the hypothalamo-pituitary adrenal axis particularly in times of stress, as in trauma, surgery or illness, growth suppression in infancy, childhood and adolescence. Raised intracranial pressure with papilloedema (pseudotumor cerebri) in children, usually after treatment withdrawal.

## **Summary of changes to protocol approved by the ethics committee**

All changes were discussed and approved by the Trial Steering committee or Chairman and, where appropriate, by the Data Monitoring Committee.

The changes made in Version 11 of the protocol reflect the major changes in the design and endpoints that are incorporated into the final version.

### *Version 2 07/11/12:*

•Change to section 3.1 to reflect that MMF was being used outside its marketing authorisation

### *Version 3 29/1/2013*

•Changes to sections 2.2.1 and 6.2 relating to assessment of adherence and risk perception. Rather than collecting prescription redemption data (version 1 and 2), we proposed tablet counts on randomly selected patients in addition to the use of iPads or similar tablets to collect the data, prior to electronic transfer to a secure server hosted by University College London. Thirdly, we proposed to pilot the questionnaires and perform quantitative interviews on a small number of participants initially recruited to Guy’s, to inform whether existing standardised questionnaires required change to suit this population.

•Change to section 7.3 relating to a change in the pharmacovigilance policy of the sponsor to ensure Important Medical Events are recorded as SAEs

### *Version 4 13/5/2013*

•Changes to sections 2.2 and 2.3 to clarify that randomisation will be stratified by site.

•Change to section 4.1 to clarify that the estimated Glomerular filtration rate measurement on which eligibility will be assessed has to be within 1 month of signing consent.

•Change to section 5.2.1 to clarify the definition of a positive HLA antibody test, which was confusing in the previous protocol versions.

•Changes to mention of recruitment targets, shifting emphasis away from precise predictions towards a more pragmatic approach that highlights recruitment will stop once minimum numbers required for statistical power have been recruited to each of the individual groups.

### *Version 5 9/7/2013*

•Change to lab PI at the Royal London Hospital.

•Change to section 3.1 clarifying the dosing of one the IMPs, Prednisolone.

•Change to reflect updated WHO definition of Diabetes mellitus (addition of HBA1C testing).

•Change to section 7.3.1, reflecting the fact that certain adverse events in this type A trial may not require reporting to the sponsor, but may still require recording in the eCRF.

### *Version 6 6/12/2013*

•Change to abandon the requirement that recruits be tested for hepatitis B core antibody, as it was hindering collection of samples for scientific analysis. Since core antibody positivity was not a contraindication to optimization, and testing was not required by King’s College London, as the infectivity of samples from core antibody positive, surface antigen-negative samples is very low, this change was felt not to compromise the trial in any way but would enhance the number of scientific samples obtained at recruitment.

•Change to allow urine as well as blood testing to rule out pregnancy.

•Changes relating to recruitment of live donors, to ensure they were tested for HIV and Hepatitis B & C if not tested within the previous 5 years, to increase volume of blood taken to 80 mls and to allow consent to be obtained by non-clinicians be allowed to consent these patients.

### *Version 7 7/4/2014*

A: Changes to maximise and standardise recruitment across sites:

•Removal of exclusion criteria “history of ongoing or previous infection that would prevent optimisation”. This criterion was vague (i.e. did not define which infections were important) and was being interpreted differently within and across sites. As the optimisation for each participant was optimised to that particular individual, immunosuppression could be tailored according to their medical history.

•Increase in the gap for the testing of eGFR from within 1 month of signing consent to within the previous 6 months of signing the consent. Participants had to have an eGRF≥30 to be eligible for the study, and by increasing the time-period to within the previous 6 months, screening for potential participants can be more efficient as measurements of eGFR from previous renal clinic appointments could be used.

B: Removal of need to perform total immunoglobulin testing.

•This measurement proved to be a difficult and expensive test to perform and was not routinely performed by all hospital laboratories. This test had originally been incorporated into the study to ensure participants were not developing MMF-induced hypogammaglobulinemia. Fortunately, this could still be detected by maintaining requirement to test for IgG, IgM and IgA.

•Testing and recording of IgG, IgM and IgA moved to every year instead of every four months. Testing for the levels of these immunoglobulins every 12 months was felt to be sufficient by the TSC sufficient for monitoring MMF-induced hypogammaglobulinemia.

C: Clarification of and changes to follow up procedures:

• Clarification that participants would see a research nurse for all trial-related procedures at follow up appointments, which will be held at the same time a participant is in routine clinic.

•Details regarding the fact that only medications being taken at the time of the follow-up would be recorded.

• Clarification of the timings for questionnaire completion in table 2.2.1.

• Patients could be given an appointment slip containing a telephone number and/or an email address to contact research nurses if their routine appointments are rescheduled.

• Clarification of the time windows for follow up appointments that were allowed without deviation to the protocol.

D: Clarification of the optimisation process for participants allocated to the unblinded HLA antibody positive arm.

•Change of optimisation timing from within 3 months of HLA antibody positivity to *ideally* within 3 months after positive screening for HLA antibody and allocation to the unblinded treatment arm or as soon as possible thereafter BUT within 8 months of positive screening. This coincided with the realisation that some patients were proving difficult to contact to arrange optimisation and the change was felt to enhance the optimsation process without affecting the outcome of the trial.

• Clarification of the way that patients will be informed about the group to which they have been allocated. Participants with HLA antibodies allocated to the unblinded arm will be told the results of this allocation as soon as possible and invited to undergo optimisation. This can be performed over the phone. Those participants in the blinded groups or in the unblinded HLA antibody negative group will be told the result of their randomisation at their next clinic visit.

• Clarification that recording details of the optimisation process will be in an Optimisation Log at each site and not in the eCRF.

E: Addition of three new sites

### *Version 8 1/7/2014*

• Extension to the time that tissue typing laboratories had to perform the randomisation of patients, from 28 to 56 days post consent. This was to optimise batching of patient serum for testing, reducing the number of experimental controls and HLA screening beads needed, and therefore the cost of screening.

• Clarification, in section 7.1, of when testing of HbA1Cshould occur.

• Clarification about tests to be performed to monitor for MMF-induced hypogammaglobulinemia.

• Changes to allow the use of results from routine clinic blood tests taken up to a week prior to consent, to minimise duplication of tests in sites where it was routine for patients to attend for blood tests prior to their clinic visit.

• Changes to allow study information to be collected via telephone to minimise time spent with each patient during busy routine clinics.

### Version 9 15/10/2014

• Clarification around timing and need for collection of experimental research samples (laboratory) at point of consent.

• Further clarification of the time windows for follow up appointments that were allowed without deviation to the protocol. Changes made to try to ensure collection of three study assessments per year.

• Change to reduce nurse paperwork: as a Type A trial with a large recruitment target, missing data around sample collection was to be coded in the eCRF but not recorded as a protocol deviation.

• Change to the principal investigator at one of the sites

### *Version 10 11/08/2015*

• Changes to two co-investigators in the Tissue Typing Laboratories and addition of three new sites.

• Change to eligibility age range, from 18-70 years to 18-75 years. Originally, we believed that non-transplant-related mortality may be higher in 71-75 year old age group, however this was re-considered and felt not to be an issue.

• Clarification of the inclusion criteria relating to timing of the eGFR used when considering eligibility.

### *Version 11 26/11/2015*

A: Change in primary endpoint

• The primary endpoint of the trial was changed from “graft failure rates over three years” to “time to graft failure with variable follow up (with a minimum of 43 months post-randomization)”. This change was required to account for the low numbers of DSA positive participants being recruited to the trial. This change will allow for a reduction in the number of DSA patients to be recruited, and a significant shortening in the expected study duration whilst maintaining the power of the study. Section 8 on sample size and statistical analyses were changed accordingly. The new primary endpoint was to be assessed remotely from patient notes once 43 months post-randomisation was achieved by all. All patients already recruited were to be re-consented to allow this change.

B: Changes to reduce costs associated with extension of the trial

• Follow-up visits changed from 4-monthly to 8-monthly to reduce nurse workload.

• End visit for each participant changed from 36 to 32 months.

• Secondary endpoint assessment changed from 36 to 32 months, except for health economics which was moved from 36 to 16 months.

• Major reduction of SAE reporting to sponsor incorporated into sections 7.2 and 7.3

C: Change in trial statistician, change in site PIs and addition of a new site.

### *Version 12 1/12/16*

• Cessation of collection of research blood samples, associated with removal of the secondary experimental ‘scientific’ endpoints. This was required by the funder, who requested that the salary costs associated with the experimental aspects of the trial be re-allocated towards supporting the primary endpoint data collection.

### *Version 13 21/11/18*

• Change to reflect inclusion of albumin:creatinine ratio as a measure of proteinuria in addition to protein:creatinine ratio and clarification that one or the other (not both) are required as one assessment of graft dysfunction.

• Change to the way that change in eGFR was to be compared between arms.

• Inclusion of proposed details for how the primary outcome data was to be collected during the period March 2020- June 2020.

• Inclusion of collection of a final sample for HLA antibody screening from all participants at their final research clinic visit at month 32.

• Clarification that results from the trial will be presented as estimates and 95% confidence intervals

• Clarification that baseline covariates were to be included in the statistical model for the primary outcome.

### *Version14 08/07/2020*

• Change to the timing of the collection of the primary endpoint as a result of the COVID-19 pandemic, in addition to the proposal to included additional sensitivity analysess for the primary endpoint and extension of the study end date.

• Inclusion of a thank you card for all participants who have taken part in the OuTSMART trial.

# A randomised controlled trial of a combined screening /treatment programme to prevent premature failure of renal transplants due to chronic rejection in patients with HLA antibodies (OuTSMART trial)

# Statistical Analysis Plan

# Version 2.4

# Version 2.4 started: 09/02/2021

ISRCTN number: ISRCTN46157828

Trial Statistician: Dominic Stringer

Signature................................................. Date......................................

Chief Investigator: Professor Anthony Dorling

Signature................................................. Date......................................

Trial Steering Committee Chair: Professor Chris Watson

Signature................................................. Date......................................

**Purpose and Scope of Statistical Analysis Strategy**

This document details the presentation and analysis strategy for the primary papers reporting results from the OUTSMART trial. It is intended that the results reported in these papers will follow the strategy set out herein; subsequent papers of a more exploratory nature will not be bound by this strategy but will be expected to follow the broad principles laid down for the principal paper(s). These principles are not intended to curtail exploratory analysis or to prohibit sensible statistical and reporting practices, but they are intended to establish the strategy that will be followed as closely as possible, when analysing and reporting the trial. Reference was made to the trial protocol (OUTSMART Trial Protocol V14 08_07_2020), ICH [[1](#_ENREF_1)] guidelines on Statistical Principles (E9) and CONSORT [[2](#_ENREF_2)] guidelines.

Contents

[1 Description of the trial 5](#_Toc531176976)

[1.1 Principal research objectives to be addressed 5](#_Toc531176977)

[1.2 Trial design including blinding 5](#_Toc531176978)

[1.3 Method of allocation of groups 7](#_Toc531176979)

[1.4 Duration of the treatment period 8](#_Toc531176980)

[1.5 Frequency and duration of follow-up 8](#_Toc531176981)

[1.6 Data collection 9](#_Toc531176982)

[1.7 Sample size estimation (including clinical significance) 12](#_Toc531176983)

[1.8 Brief description of proposed analyses 13](#_Toc531176984)

[2 Data analysis plan – Data description 14](#_Toc531176985)

[2.1 Descriptives by HLA status 14](#_Toc531176986)

[2.2 Recruitment and representativeness of recruited patients 14](#_Toc531176987)

[2.3 Baseline comparability of randomised groups 15](#_Toc531176988)

[2.4 Adherence to allocated treatment and treatment fidelity 15](#_Toc531176989)

[2.5 Loss to follow-up and other missing data 16](#_Toc531176990)

[2.6 Adverse event reporting 16](#_Toc531176991)

[2.7 Assessment of outcome measures (unblinding) 16](#_Toc531176992)

[2.8 Descriptive statistics for outcome measures 16](#_Toc531176993)

[2.9 Description of therapists/therapies 16](#_Toc531176994)

[3 Data analysis plan – Inferential analysis 16](#_Toc531176995)

[3.1 Main analysis of treatment differences 16](#_Toc531176996)

[3.1.1 Analysis of primary outcomes 16](#_Toc531176997)

[3.1.2 Analysis of secondary outcomes 17](#_Toc531176998)

[3.1.3 Statistical considerations 17](#_Toc531176999)

[3.1.4 Sensitivity analyses 18](#_Toc531177000)

[3.1.5 Planned subgroup analyses 18](#_Toc531177001)

[3.2 Exploratory analyses 18](#_Toc531177002)

[3.3 Exploratory mediator and moderator analysis 18](#_Toc531177003)

[3.4 Interim analysis 18](#_Toc531177004)

[4 Software 18](#_Toc531177005)

[B) ECONOMIC ANALYSIS AND ADHERENCE/RISK ASSESSMENT PLANS 19](#_Toc531177006)

[C) SCHEDULE OF ASSESSMENTS AND MEASURES 21](#_Toc531177007)

[D) Reference list 24](#_Toc531177008)

[E) Amendments to version 1.0 24](#_Toc531177009)

A) QUANTITATIVE ANALYSIS PLAN

Investigators:

Principal investigator: Anthony Dorling

Trial manager: Leanne Gardner

Trial statisticians: Dominic Stringer, Janet Peacock

Trial health economist: Paul McCrone

## Description of the trial

Protocol Publication:

Anthony Dorling, Irene Rebollo Mesa, Rachel Hilton, Janet Peacock, Robert Vaughan, Richard Baker, Brendan Clarke, Raj Thuraisingham, Matthew Buckland, Michael Picton, Susan Martin, Richard Borrows, David Briggs, Robert Horne, Paul McCrone and Caroline Murphy. Can a combined screening /treatment programme prevent premature failure of renal transplants due to chronic rejection in patients with HLA antibodies: Study protocol for the multicentre randomised controlled OuTSMART trial. (Trials. 2014 Jan 21; 15:30. doi: 10.1186/1745-6215-15-30)

### Principal research objectives to be addressed

Primary objective;

Compare the time to graft failure in patients with HLA Ab who receive an optimized anti-rejection medication intervention (‘treatment’), with that in a control group with HLA Ab who remain on their established immunotherapy and whose clinicians are not aware of their Ab status.

Secondary objectives;

a) Determine the time to graft failure in patients randomized to ‘unblinded’ HLA Ab screening, compared to a control group randomized to ‘blinded’ HLA Ab screening.

b) Determine whether ‘treatment’ influences patient survival

c) Determine whether ‘treatment’ influences the development of graft dysfunction as assessed by presence of proteinuria (Protein:Creatinine Ratio > 50 or Albumin:Creatinine Ratio > 35) and change in estimated Glomerular Filtration Rate (eGFR).

d) Determine whether ‘treatment’ influences the rates of acute rejection in these groups

e) Determine the adverse effect profiles of ‘treatment’ in this group, in particular whether they are associated with increased risk of infection, malignancy or Diabetes Mellitus (DM).

f) Determine the cost effectiveness of routine screening for HLA Ab and prolonging transplant survival using this screening/treatment protocol.

g) Determine the impact of biomarker screening and “treatment” on the patients’ adherence to drug therapy and their perceptions of risk to the health of the transplant.

### Trial design including blinding

This is a prospective, open labelled, randomised marker-based strategy (hybrid) trial design, with two arms stratified by biomarker (HLA Ab) status. Recruitment will take place in 12 renal transplant units, recruiting for 45 months with recruits followed up intensively for at least 32 months (maximum 64 months) and primary endpoint assessed by remote evaluation after 43 months post-randomisation is achieved by all. The trial design is represented in the flow diagram in section 2.3, showing the number of patients anticipated to be in each group by the end of the trial, based on sample size calculations, consent rates, eligibility and estimated fall-out. Using the flow diagram (top-to-bottom) as a guide: recipients of cross-match negative transplants aged 18-75, > 1-year post-transplant with an eGFR ≥ 30 will consent to the screening/treatment process.

The first stratification will result from blood test screening for HLA Ab. Approximately 35% will be HLA positive, with ~65% negative. The HLA Ab+ patients will be further screened with single antigen beads to determine whether DSA are present (~1/6 DSA and 5/6 non-DSA). Thus, biomarker stratification leads to three groups (DSA+, non-DSA+ and HLA Ab-neg).

The second stratification will be based on current immunosuppression, to ensure balanced numbers already on Tac or MMF in each group. The final stratification will be by site.

HLA Ab positive patients will be randomized 1:1 into either Blinded Standard Care or Unblinded Biomarker led-care. Patients in the former (groups A1 & A2 in the flow chart in 2.3) will be blind to their biomarker status and will remain on baseline immunotherapy, whereas patients in the latter (groups B1 and B2 in the flow chart) will know their HLA Ab status and will be offered “treatment”.

HLA Ab-negative patients will remain on their existing immunotherapy and randomized 1:1 into either Blinded (group C) or Unblinded groups (D), with only the latter knowing their HLA Ab status. Both these groups will receive regular Ab status monitoring for the first 3 years. Those patients who become positive during subsequent screening rounds (~10% per year) will be moved to the appropriate HLA Ab positive groups (DSA+ or non-DSA+) for final data analysis.

All patients in group D found to be positive on second or subsequent rounds will be offered the same “treatment” as those patients who were positive in the first screening round and be intensively followed up for an additional 32 months from the time they become positive. Thus, the maximum amount of time any single patient may remain in intensive follow up is 64 months^^[[26]](#footnote-26)^^. New patients will be recruited to the study at each successive screening round.


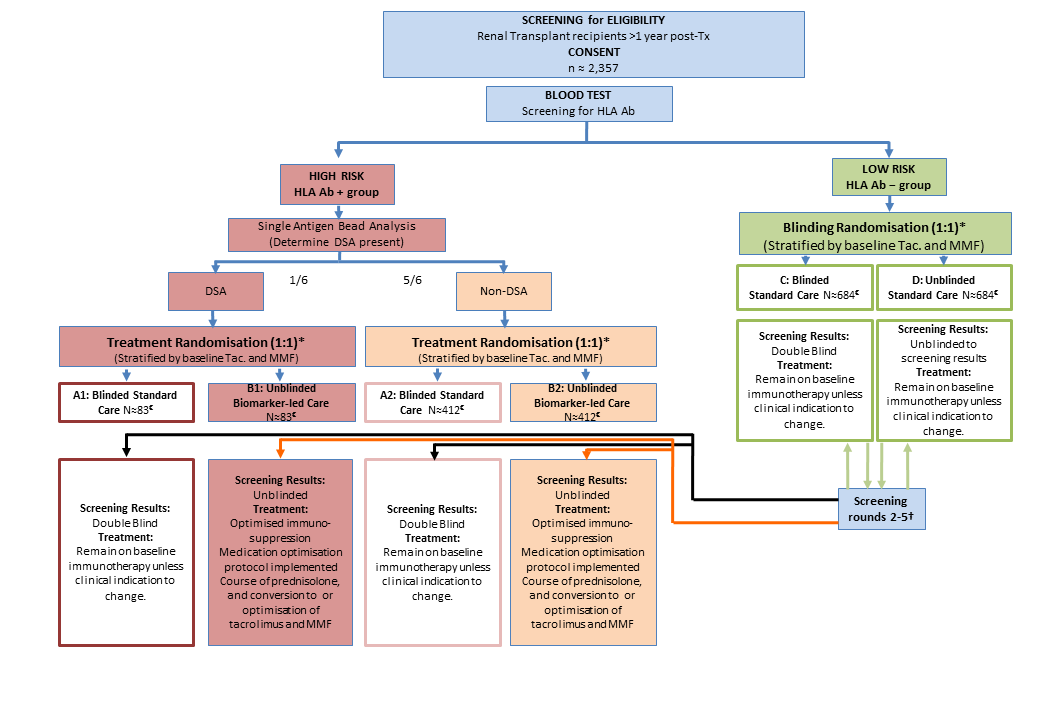


*Randomisation performed on results of a recruit’s first screening test. Those with HLA Ab undergo no further screening as part of the trial (but serum will be stored for analysis of HLA Ab profiles later). **^†^**Those initially HLA Ab-negative undergo routine screening every 8 months. THERE IS NO SECOND RANDOMIZATION: If a recruit allocated to Blinded standard care (group C) becomes HLA Ab positive (black lines), he/she remains in Standard care group (group A1 or A2). If in unblinded standard care group (D), they change to unblinded biomarker-led treatment care (group B1 or B2) (orange lines). € Numbers in each group are those anticipated at the end of study.

Figure 1. Trial design flow diagram

### Method of allocation of groups

Prior to randomisation but after consent, site staff will register all recruits online and each will be assigned a MACRO PIN. Samples from all recruits will be sent to the HLA laboratory, along with this PIN and a sample request form containing other information required for randomisation.

Laboratory staff will screen for HLA Ab and perform single antigen bead testing on positive screening samples to check for the presence of DSA. Once this information is known, the lab staff will access the randomisation system and randomise the patient, using the HLA Ab results and information on the sample form to stratify. Randomisation will be further stratified by centre and previous immunosuppression. In all sites, the PI’s and nurses will be automatically emailed and the system will tell them whether the patient is in the blinded or unblinded groups. If in the unblinded group, it will feed back HLA Ab status to the PI. Unblinded patients will be identified by blue stickers to be appended to the notes and all future clinical samples. The system will tell the trial staff to enter HLA Ab-negative patients into the subsequent 8 monthly screening rounds, and also whether the patients have been selected to provide future samples for 4 monthly scientific analysis (for transfer to the CIs laboratory). This information will be relayed using a ‘star’ on the blue labels, appended to their laboratory request forms thereafter.

Blinded patients will have green stickers/labels. HLA Ab status will not be fed back to the PIs or trial staff. A ‘star’ will be used to tell the trial staff which recruits have been selected to provide 4 monthly samples for transfer back to the CIs lab for scientific analysis. All these patients will have samples taken 8 monthly for HLA Ab screening. Once inside the lab, the lab staff will use their knowledge of the HLA status to determine those from HLA Ab-negative patients which will undergo screening. The samples from HLA Ab positive patients will be discarded.

On the second and subsequent HLA Ab screening rounds, the lab staff will update the randomisation system. The results from patients in the unblinded groups only will be forwarded to the PI and laboratory staff, via email. This will indicate whether status has changed and trigger the initiation of the treatment protocol in those that have changed from HLA Ab negative to positive.

Randomisation will be via the online King’s Clinical Trials Unit randomisation system.

Laboratory staff at each recruiting site, with access to HLA Ab results, will be provided with a unique username and password to access the randomisation system. Password access must be authorised by the trial manager in all cases and request directly from sites will not be processed. Access to the system is via <https://cturandomisation.iop.kcl.ac.uk/OUTSMART>, clicking the ‘randomisation – advanced’ link and selecting the OuTSMART Trial.

### Duration of the treatment period

All treatments will be introduced on the basis that they will be tailored to the individual patient, according to compliance, tolerance and achievement of target levels (for Tac). Failure to tolerate one or more of the components of the protocol (or refusal to take any of the agents) will not be used as a reason for withdrawal from the study.

### Frequency and duration of follow-up

Following recruitment to the trial, the patients will undergo 32 months of intensive follow up involving 8-monthly clinic visits post-randomisation, except in the following scenario; a patient in groups C or D who becomes Ab positive during the initial 32 months follow up will transfer to the relevant Ab+ group and undergo intensive follow up for a further 32 months from date of transfer. Therefore, the maximum amount of time that any single patient may remain in intensive follow up is 64 months.

The optimised treatment protocol will be introduced within the first 3 months in those HLA Ab+ patients allocated to this group. Recruits will be seen *up to* twice weekly during this period (maximum of 6 extra clinic appointments are envisaged), though they should be on maintenance dose prednisolone 7 weeks after initiating optimisation. During this period, they will have full blood count (as above), creatinine, Na^+^, K^+^, glucose, calcineurin inhibitor trough levels and blood pressure monitored according to the trial protocol. Once stabilized, they will be seen at least 8 monthlies in transplant clinic. Patients allocated to all other groups will be seen at least every 8 months in transplant clinic for formal study assessments. Patients may be seen at other times during this period, according to clinical need, but study assessments should be done within the time parameters established in the protocol.

Once every 8 months the following will be recorded. a) Weight and bp; b) Full blood count (minimum Hb, WCC, platelets); c) Biochemical series (creatinine, Na^+^, K^+^, bicarbonate, calcium, CRP, glucose); d) MDRD eGFR on latest creatinine; e) calcineurin inhibitor trough levels; f) protein: creatinine ratio on urine sample; g) total immunoglobulin levels; h) episodes of infection, malignancy or new DM; i) episodes considered to be adverse events. Every 16 months a lipid profile will be performed.

Once every 8 months, HLA Ab-negative patients will undergo further screening for HLA Ab (see above). At the end of the study, all patients will undergo a final test for HLA Ab status.

Upon completion of the 32- to 64-month intensive follow up, consisting of 8-monthly research visits, the participants will be told by the research nurses that they no longer need to attend to clinic for research visits, but their regular clinic visits will continue. At the last intensive follow up visit a HLA Ab sample will be collected from all participants. The original plan was for the study to conclude once the final participant reached 43 months post-randomisation in June 2020 and in the three months prior to the conclusion of the study, data regarding the primary endpoint was to be collected from patient notes for all participants

Due to the coronavirus pandemic in the UK in 2020 most clinical trial activity, including this trial, was severely limited and so it was not possible to rely on the original plan to obtain the primary endpoint data between April and June 2020. For this reason, the best alternative was to obtain the primary endpoint data from patients’ clinic notes once the coronavirus situation had eased and clinical trial activity re-started nationwide. Evidence for graft failure or death will now be taken from the participants’ last hospital contact prior to March 16th, 2020. These data, which will reflect participants’ pre-COVID status, will be used for the primary endpoint analysis. Evidence of graft failure or death will also be taken from participants’ notes from their most recent hospital contact at the point of a final assessment between September 1st, 2020 and November 30th, 2020. During this designated three-month window, endpoint data will be collected from each patient’s notes only once; the window is simply for pragmatic reasons to allow centre research staff sufficient time to check all patients’ notes. These data, reflecting status post-onset of COVID crisis, will be used for a sensitivity analysis. The trial will conclude on November 30th, 2020.

### Data collection

Eligibility Screening

- Potentially eligible patients will be approached at a routine clinic appointment by the PI or research nurses and given printed and verbal information about the trial. They will have the opportunity to return for a second consultation within a few days to give informed consent for recruitment into the study or to do this on their next routine appointment. Alternatively, eligible patients will be sent information about the study through the post, for discussion and consent at their next routine appointment. Following consent, full eligibility criteria will be reviewed. This may include testing for chronic viral disease (if no such test within last 5 years) or pregnancy (if history suggests possibility of pregnancy).

Inclusion Criteria

- • Sufficient grasp of English to enable written and witnessed informed consent to participate.
- • Renal transplant recipients >1 year post-transplantation, male or female
- • Aged 18-75 years
- • Estimated glomerular filtration rate (eGFR by 4 variable MDRD) of ≥30 (within the previous 6 months of signing consent or taken at screening if not done in the previous 6 months).

Exclusion Criteria

- • Recipient requiring HLA desensitisation to remove antibody for a positive XM transplant
- • Recipient known already to have HLA antibody WHO HAS RECEIVED specific intervention for that antibody or for CAMR/chronic rejection
- • Recipient of additional solid organ transplants (e.g. pancreas, heart, etc.).
- • History of malignancy in previous 5 years (excluding non-melanomatous tumours limited to skin)
- • HBsAg+, HepC IgG+ or HIV+ recipient (on test performed within previous 5 years)
- • History of acute rejection requiring escalation of immunosuppression in the 6 months prior to screening.
- • Patient enrolled in any other studies involving administration of another IMP at time of recruitment
- *The following exclusion criteria are based on information contained within the SMPcs of the IMPs*
- • Known hypersensitivity to any of the IMPs
- • Known hereditary disorders of carbohydrate metabolism
- • Pregnancy or breastfeeding females (based on verbal history of recipient)
- • Pre-menopausal females who refuse to consent to using suitable methods of contraception throughout the trial.

Baseline

Post consent, patients who have not been screened for HIV or hepatitis B/C within the last 5 years will need to have additional screening tests for these viruses. Female patients who report they may be pregnant will have a blood test for beta-HCG levels. Once eligibility criteria have been met, the following baseline data will be recorded at recruitment:

a) Weight and bp;

b) Sex and ethnicity;

c) Age and date of birth;

d) HLA type and that of donor kidney (if known);

e) Any significant past medical history, including history of diabetes mellitus, cause of renal failure, details of previous transplants and cause of graft loss, evidence of sensitisation pre-transplantation (panel reactive antibody and antibody specificities if known);

f) medication list and doses;

g) PCR on urine sample

All patients will then have blood taken for;

i) Baseline clinical parameters: a) Full blood count (minimum Hb, WCC, platelets); b) Biochemical series (creatinine, Na^+^, K^+^, bicarbonate, calcium, CRP, lipid profile, glucose); c) MDRD eGFR on latest creatinine; d) current calcineurin inhibitor 12 hour trough levels (as appropriate); e) Total Immunoglobulin levels

ii) Scientific analyses: 50-60 mls blood for separation of PBMC and 20mls for serum storage.

iii) Analysis of HLA Ab status (10mls clotted blood), as described above, which will allow randomization to proceed.

All patients will be asked to complete questionnaires to assess attitudes to risk/adherence.

Primary outcome measures

The primary endpoint is time to graft failure in HLA Ab positive patients randomized to biomarker led care groups vs. time to graft failure in HLA Ab + patients randomized to standard care groups assessed at a minimum of 43 months post-randomisation achieved by all (see above Section 1.5) for adjustment to final data collection following the coronavirus pandemic, consequently a few participants may not reach the minimum of 43 months for the primary endpoint. Graft failure will be defined as re-starting dialysis or requiring a new transplant.

Secondary outcome measures

The secondary clinical endpoints are:

• Time to graft failure in patients randomized to blinded HLA Ab screening vs those randomized to unblinded screening. Graft failure will be defined as re-starting dialysis or requiring a new transplant.

The following endpoints will be assessed at end of intensive follow up (32 months):

• Patient survival.

• Graft dysfunction, as assessed by two separate measures, presence of proteinuria (Protein Creatinine Ratio >50 or Albumin/Creatinine Ratio > 35) and change in estimated Glomerular Filtration Rates over 32 months.

• Rates of biopsy-proven rejection.

• Rates of culture- or polymerase chain reaction (PCR)-positive infection, biopsy-proven malignancy and DM.

• Health economic analysis of outcomes in intervention vs. control groups.

• Analysis of adherence and perceptions of risk in BLC groups.

Adverse Events

This trial fulfils the criteria for a ‘Type A’ trial (i.e. risk no higher than that of standard care) Therefore, there will be reduced reporting of adverse events. Definitions of expectedness reported below are based on those listed in the SmPC for each IMP.

**Adverse Event (AE)**: Any untoward medical occurrence in a subject to whom a medicinal product has been administered including occurrences which are not necessarily caused by or related to that product.

**Adverse Reaction (AR)**: Any untoward and unintended response in a subject to an investigational medicinal product which is related to any dose administered to that subject.

**Unexpected Adverse Reaction (UAR)**: An adverse reaction the nature and severity of which is not consistent with the information about the medicinal product in question set out in the summary of product characteristics (SmPC) for that product.

**Serious adverse Event (SAE)**: Serious Adverse Reaction (SAR) or Unexpected **Serious Adverse Reaction (USAR)**: Any adverse event, adverse reaction or unexpected adverse reaction, respectively, that

Results in death;

Is life-threatening;

Required hospitalisation or prolongation of existing hospitalisation;

Results in persistent or significant disability or incapacity;

Consists of a congenital anomaly or birth defect.

Although not a serious adverse event, any unplanned pregnancy should be reported via the SAE reporting system as stated below.

### Sample size estimation (including clinical significance)

The primary purpose of this trial is to demonstrate superior outcomes using a defined treatment strategy in biomarker (HLA Ab) positive patients and at the same time demonstrate non-inferior outcomes when the unblinded screening strategy is applied to the entire patient population. Time to graft failure has been chosen as a clinically relevant primary outcome. As a reference for power calculations, we have used the observed failure rates reported by Lachmann et al. [8] for HLA Ab+ and HLA Ab-neg patients. Since failure rates differ between DSA+ and non-DSA+ patients, sample size calculations have been carried out separately for these groups. Following these calculations, we have estimated the number to be screened, based on expected drop out rates, expected screening results and eligibility criteria (see below).

We have based our estimates of the differences in primary outcome between groups on two things; first, the results of our preliminary data from patients with CR treated with a similar regime as used here; second, our assessment that large differences in primary outcome will be needed to make the screening programme cost-effective.

Hypotheses and power calculations:

1) Superiority on Biomarker Positive Patients:

1.1) A1>B1:

HLA Ab+ patients, with DSA, randomized to standard care (A1) will show higher graft failure rates than patients randomized to biomarker-led care (B1). We hypothesize that the experimental treatment will bring the failure rate in group B1 down to that of non-DSA patients in standard care (A2). Assuming that 30% of patients with DSA randomised to standard care (A1) will have experienced chronic rejection (CR) by 3-years follow up, we expect treatment optimisation to reduce the rate of CR in DSA patients randomised to group B1 down to 16% at 3-years follow up (rate observed in patients with non-DSA). This corresponds to a Hazard ratio (HR) of 0.489. The expectation is for 11% and 21% of CR among patients with DSA in in group A1 at 1 and 2-years follow up respectively (as in [8]), and extrapolating based on a HR of .489, we expect BLC to reduce those CR to 5.5%, and 10.89% at 1 and 2-years.

Using a variable follow up design assuming an average accrual monthly rate of 3.6 patients per month, and a minimum follow up time of 43 months, recruiting 165 patients with DSA would allow us to observe 23/83 (28%) events of CR in patients under biomarker led care (B1), and 39/82 (47%) in the standard care group (A1). This would provide 80% power and 5% type I error, for a two-sided log-rank test.

1.2) A2>B2:

HLA Ab+ patients, with non-DSA, randomized to standard care (A2) will show higher graft failure rate than patients randomized to biomarker-led care (B2). We hypothesize that the experimental treatment will bring the failure rate in group B2 down to that of biomarker negative patients in standard care (C). Assuming that 16% of patients with NDSA randomised to SoC will have experienced chronic rejection (CR) by 3-years follow up, we expect treatment optimisation to reduce the rate of CR in NDSA patients randomised to BLC down to 6% at 3-years follow up (rate observed in patients without HLA antibodies).

This corresponds to a Hazard ratio of 0.351. Based on Lachman et al. the expectation is for 3% and 11% of CR among patients with NDSA in SoC at 1 and 2-years follow up respectively, and extrapolating based on a HR of 0.351, we expect BLC to reduce those CR to 1.1%, and 4.1% at 1 and 2-years.

Using a variable follow-up design (patients followed until failure, drop out or end of minimum follow up), assuming an average accrual monthly rate of 15.5 patients per month, and a minimum follow up time of 22.4 months, recruiting 296 patients with NDSA, would allow us to observe 8/149 (5.3%) events of CR in patients under BLC, and 21/147 (14%) in the SoC group (total duration = 41.5 months). This would provide 80% power to determine a statistically significant difference between SoC and BLC, using a log-rank test, with a 2-sided type-I error rate of 5%.

The numbers enrolled in groups A & B include those patients initially enrolled in groups C or D who become HLA Ab+ during re-screening.

2) Non-inferiority of all Unblinded patients compared to all Blinded patients:

2.1) A1+A2+C ≥ B1+B2+D:

All patients randomized to unblinded screening will show equal or lower graft failure rates than all patients randomized to blinded screening, irrespective of biomarker status. At the end of the trial, we expect 58% of patients to be in the HLA Ab negative groups, 7% DSA+ groups and 35% non-DSA+ groups (after drop-outs). At the time of planning the OuTSMART study, we calculated that based on all assumptions above, all patients randomised to SoC combined would experience 13.9% of CR.

We established a non-inferiority limit of 5% absolute difference in rate of CR at 3-years, so that the BLC group would be considered inferior to SoC with a CR rate of 18.9% or higher (expectation under the null hypothesis). This corresponds to a HR of 1.4 under the null hypothesis, and a HR of 0.63 under the alternative. Recruiting 672 patients over a period of 13.2 months, at an average accrual rate of 51 patients per month, and a minimum follow up of 18.21 months, would allow us to observe 22/337 (6.5%) events of CR in the SoC group, and 32/335 (9.5%) in the BLC group.

This would provide 90% power to demonstrate non-inferiority with a one-sided 95% Confidence Interval of the HR estimated using a Cox regression model. Given the above proportions, this requires enrolling 336 patients in each of groups C and D and this should allow 423 total patients to reach the primary endpoint (i.e. remain negative (after dropouts) at the end of their three year follow-up).

Based on an overall expected proportion of 7% DSA participants (including from re-screening rounds) we will need to recruit 2357 patients overall to recruit the target of 165 DSA patients. Because of this requirement to recruit sufficient DSA participants, the recruits to the other groups are likely to be more than the minimum required for at least 80% statistical power for the individual hypotheses.

### Brief description of proposed analyses

Analyses will be carried out by the trial statistician. In the first instance data will be analysed under intention-to-treat assumptions (i.e. analyse all those with data in groups as randomised irrespective of treatment received). As per Section 1.7, in addition using all participants, all outcomes will be analysed separately within the subgroups of HLA Positive DSA participants and HLA Positive Non-DSA participants.

Those patients who become positive during subsequent screening rounds will be moved to the appropriate HLA Ab positive groups (DSA+ or non-DSA+) for analysis. These participants will be included from the time they became HLA Ab positive (date sample for screening was taken). For the analysis using all participants, these participants will be included from the time of randomisation.

### Further changes in procedures in response to Covid-19

Subsequent to the changes outlined in Section 1.5 (which detail changes made to primary endpoint collection following the COVID-19 pandemic), database lock was intended to be completed in February 2021, and the analysis completed by April 2021, with end date for OUTSMART the 30^th^ April 2021. The remaining requirements prior to database lock taking place and analysis starting were i) that all data had been cleaned and ii) that final monitoring/source data verification of the primary endpoint had been completed as planned (100% of all graft failures, 25% of remaining participants). The monitoring is undertaken by King’s Health Partners Clinical Trials Office (KHP-CTO). The KHP-CTO had intended to visit all sites to finish final monitoring of the primary endpoint (graft failure) data by the end of January 2021. However, monitoring was suspended as the latest wave of the COVID-19 pandemic occurred in Winter 2020/2021 and this meant that research nurse teams who were responsible for making source data available for monitoring in each centre were again redeployed. For these reasons, it was foreseen that the database lock could not be completed according to the planned timeline.

A 6 month no-cost extension to the project to 31^st^ October 2021 was granted from the trial funder, National Institute for Health Research Efficacy and Mechanism Evaluation Programme (NIHR EME), with the intention that monitoring would re-commence once possible. However, the shift in timelines meant that the final cleaning and statistical analysis would be delayed which in turn impacted staffing, specifically the Trial Manager and Statistician, both essential at this key end stage of the trial but whose input could not be guaranteed through to the new study end. For these reasons a new plan was devised that optimised the time that the trial manager and statistician had available:

- The database will be ‘frozen’ (temporarily locked) following completion of all data checking apart from the remaining KHP monitoring. This will allow the statisticians to run the analyses unblinded with the aim of completing them in April 2021 in keeping with the existing timeline.
- The dated statistical report will be generated from these analyses and sent to the DMEC so they can attest to the validity of the process and confirm they are happy for this report to be shared with the Chief Investigator.
- The Chief Investigator will then receive the report in order for the team to draft a preliminary Clinical Study Report and preliminary primary publication. These will not be finalised, however.
- Once KHP monitoring of the primary endpoint is able to be completed and any queries resolved, the database will be finally locked and the analyses re-run.
- It is expected that at most there would only be minor changes to the database following monitoring queries from the KHP-CTO, and so the results and their interpretation are very unlikely to change. It will be ensured that changes to the data are only made in response to monitoring queries raised by the KHP-CTO CRA.   The MACRO EDC system has functionality to record any changes made between the database being frozen and final database lock and these will be checked by the KHP-CTO to confirm that all changes were made in response to monitoring queries only.
- The drafts of the preliminary clinical study report and preliminary primary publication will be updated with final results post database lock. The preliminary report results will be included as an appendix in both the final clinical study report and primary publication to ensure transparency.

## Data analysis plan – Data description

### Descriptives by HLA status

All descriptives will be broken down by HLA status. Where appropriate, descriptives for HLA Ab Positive participants may be further broken down by whether they were HLA Positive at randomisation or through re-screening. Those who became HLA Ab Positive at re-screening may also be described separately prior to becoming HLA Ab Positive and after becoming HLA Ab Positive.

### Recruitment and representativeness of recruited patients

CONSORT flow chart will be constructed (1) – see Figure 2. This will include the number of eligible patients, number of patients agreeing to enter the trial, number of patients refusing; number of HLA Ab positive and negative patients, then by treatment arm: the number continuing through the trial, the number withdrawn or lost to follow-up and the numbers excluded/analysed.


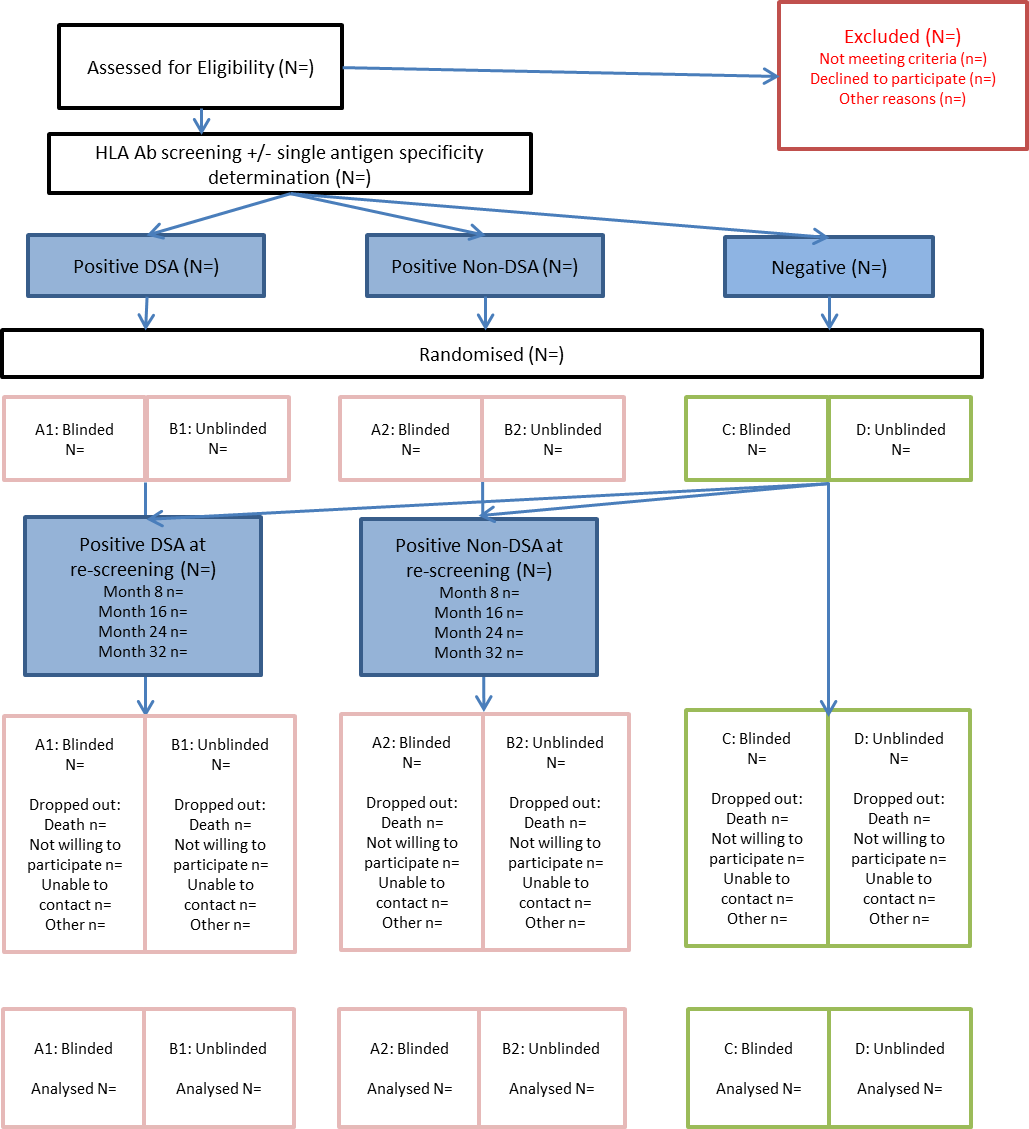


Figure 2. Template CONSORT diagram for OuTSMART trial

### Baseline comparability of randomised groups

Baseline descriptions of participants by trial arm within HLA status and overall: means and standard deviation or numbers and proportions as appropriate. Especially relevant factors are age, sex, HLA mismatches, baseline eGFR, site, previous treatment, and time from transplantation. No significance testing will be performed.

### Adherence to allocated treatment and treatment fidelity

Antibody positive patients randomized to biomarker led care who take the full optimization protocol (three drugs) will be compared to those who don’t take the full protocol (less than three drugs) with respect to baseline variables and outcomes.

### Loss to follow-up and other missing data

The proportions of participants with missing data for each variable will be summarised in each arm and at each time point. The baseline characteristics of those missing follow up will be compared to those with complete follow up. The reasons for withdrawal from the trial will be summarised.

### Adverse event reporting

Adverse events (AE), adverse reactions (AR), serious adverse events (SAE) and serious adverse reactions (SAR) will be summarised by group (within trial arm and HLA status) as proportions and 95% confidence intervals.

### Assessment of outcome measures (unblinding)

Evidence for unblinding of HLA status (within blinded arm) will not be assessed.

### Descriptive statistics for outcome measures

Frequencies and descriptive statistics of all primary and secondary outcome measures will be reported with estimates and 95% confidence intervals.

### Description of therapists/therapies

Immunosuppressive drugs and doses prescribed to each group of patients will be summarized.

## Data analysis plan – Inferential analysis

### Main analysis of treatment differences

#### Analysis of primary outcomes

Statistical analysis will be on an intention to treat basis. Patients who become positive during the follow up will be included in the appropriate group (group labels refer to flow diagram in figure 1).

1. Superiority:

H_0_: h_A1_(t)= h_B1_(t) & h_A2_(t)= h_B2_(t)

H_1_: h_A1_(t)≠ h_B1_(t) & h_A2_(t)≠ h_B2_(t)

In order to test superiority for the primary outcome in the Biomarker (HLA Ab) positive groups (Hypothesis 1.1 and 1.2), we will use Cox proportional hazards regression models to estimate the graft failure hazard ratio between the biomarker led care and standard care groups and test at the 5% level of significance. Results will be given as estimates and 95% CIs. Within the model, we will adjust for previous immunosuppression regimen and research site (as these are the randomisation stratification factors) for increased statistical efficiency.

We will check the proportional hazards assumption by examining Kaplan-Meier plots and by testing for an interaction between group (BLC or SC) and time to graft failure within the model.

1. Non-inferiority:

H_0_: h_Unblind_(t) / h_Blind_(t) ≥ δ

H_1_: h_Unblind_(t) / h_Blind_(t) < δ

In order to test for non-inferiority of the unblinded groups compared to the blinded groups (hypothesis 2.1), we will use Cox proportional hazards regression models to estimate the graft failure hazard ratio. We will adjust for the stratification factors in the model as outlined above and check the proportional hazards assumption by examining Kaplan Meier plots and by testing for an interaction between unblinded/blinded group and time to graft failure. We will conclude non-inferiority if H_0_ gets rejected at 5% significance, and the corresponding upper bound of the 95% CI for the hazard ratio excludes the limit δ (hazard ratio of 1.4).

#### Analysis of secondary outcomes

All secondary outcomes will be analysed comparing standard care versus biomarker led care groups within the HLA positive DSA participants and within HLA positive non-DSA participants as well as between unblinded and blinded groups overall, as per the primary outcome analysis. We will use a similar procedure using Cox proportional hazards regression for the analysis of secondary time to event (survival) outcomes. Where numbers allow, secondary binary outcomes will be analysed using logistic regression with adjustment for stratification factors. Where numbers are too small for this, the Z-test or Fisher’s exact will be used. For continuous secondary outcomes, linear regression will be used (or linear mixed models where accounting for repeated measures), adjusting for baseline values of the outcome and stratification factors. Transformations will be considered where data is skewed.

Results will be given as estimates (odds ratios or differences in proportions) and 95% CIs.

#### Statistical considerations

Missing baseline data

Missing baseline data should not be an issue for the primary analysis. Some extensions to this analysis may use other baseline variables; if these contain missing data, the number with complete data will be reported and they will be imputed using a method suitable to the variable as per the recommendations of White and Thompson (2).

Missing outcome data

If post treatment variables such as compliance with treatment are found to be predictive of drop out, multiple imputation will be considered.

Method for handling multiple comparisons

No formal adjustment of p-values for multiple testing is necessary. However, care will be given to the interpretation of inference for the numerous secondary outcomes.

Method for handling non-compliance (per protocol/CACE analyses)

An exploratory per protocol analysis will be carried out comparing time to graft failure in only those participants who were optimised to the full treatment protocol (as defined in Section 3.1 of the Trial Protocol) in the unblinded arm against all blinded participants, within both the HLA Ab+ DSA and HLA Ab+ non-DSA groups. These analyses will be clearly stated as exploratory in the primary paper/report and will be interpreted accordingly.

If there are concerns about compliance, complier average causal effect (CACE) analysis will also be performed.

Model assumption checks

The proportional hazards assumption will be checked for the primary outcome model by testing for an interaction with time. For secondary outcomes, where we assume normally distributed outcomes; this will have been checked when describing the data and if substantial departures from normality occur, transformations will be considered. Residuals will be plotted to check for normality and inspected for outliers.

#### Sensitivity analyses

Due to the COVID-19 pandemic, the original primary outcome endpoint was moved forward and will be collected from notes to prior to March 2020 (see section 1.5). This unavoidable protocol change may lead to a slight reduction in statistical power for the primary analysis. A sensitivity analysis will be carried out for the primary outcome using, additionally, data from participants’ most recent hospital contact as of the assessment period between September 1st, 2020 and November 30th, 2020. This sensitivity analysis may have increased power to detect a difference due to the longer observation time but might be biased due to the effects of the pandemic on the study participants. The sensitivity analysis will otherwise be carried out in exactly the same way for each of the hypotheses (for the primary outcome only). 

#### Planned subgroup analyses

An exploratory subgroup analysis will be carried out within the HLA Ab+ non-DSA group using only those participants within this group that have definite non-DSA (as opposed to those participants who are classified as non-DSA as it is unknown whether they had DSA) This analysis will be clearly stated as exploratory in the primary paper/report and will be interpreted accordingly.

There are no other planned subgroup analyses beyond those described by the stratifiers.

### Exploratory analyses

There will be no other exploratory analyses in the primary paper.

### Exploratory mediator and moderator analysis

There will be no exploratory mediator or moderator analyses in the primary paper and so these will not be covered here; subsequent papers may explore this.

### Interim analysis

There are no planned interim analyses of the primary outcome, but the statistician will carry out descriptive analysis of demographic variables, adverse events and other relevant factors to report during DMC meetings. The proportions of patients presenting HLA antibodies will be closely monitored to check that assumptions on which sample size calculations are made hold.

## Software

Data management: An online data collection system for clinical trials (MACRO; Elsevier) will be used. This is hosted on a dedicated server at KCL and managed by the KCTU. The KCTU Data Manager will extract data periodically as needed and provide these in comma separated (.csv) format.

Statistical analysis: Stata version 15.1 and/or R will be used for all statistical analyses.

# B) ECONOMIC ANALYSIS AND ADHERENCE/RISK ASSESSMENT PLANS

Heath economic objectives

Economic measures

Effectiveness: The economic evaluation will adopt an NHS perspective. Costs of intervention include the cost of screening beads and enhanced drug costs. Other costs will be calculated by combining service use data measured with the Client Service Receipt Inventory and hospital records where available with appropriate unit cost information. Costs will be compared between groups using bootstrap methods to take account of likely skewed data distributions. Costs will be combined with clinical outcomes ((1) graft failure; (2) patient survival (3) graft dysfunction (see defn. above); (4) acute rejection; (5) culture-positive infection, malignancy or diabetes) and (6) QALYs derived from the EQ-5D, a health-related quality of life measure Incremental cost-effectiveness ratios will be computed to indicate the extra cost incurred to achieve an extra unit of outcome. Uncertainty around these ratios will be assessed using cost-effectiveness planes. Net benefit per patient will be calculated by multiplying QALY gains by the assumed maximum willingness-to-pay for a QALY (£20,000) and subtracting costs. Alternative values for a QALY will also be used and a cost-effectiveness acceptability curve derived.

Adherence and Risk Assessment.

Perceived risk of transplant failure will be measured using a specifically adapted version of the illness perceptions questionnaire (IPQ-brief). Inferential statistics (independent t-test if data is approximately normally distributed, Mann-Whitney U test otherwise) will be used to determine if a difference exists between Ab positive unblinded and blinded participants. Differences between the blinded and unblinded HLA Ab screening groups will also be investigated.

Participants’ report of adherence behaviour will be assessed using the Medicines Adherence Report Scale (MARS), a valid and reliable scale that has been previously used to assess adherence in renal transplant recipients. Self-report measures have the advantage of being inexpensive and non-intrusive. However, it is known that self-report underestimates the true extent of nonadherence because of inherent self-presentational and recall biases. Self-presentational bias occurs when respondents may be reluctant to admit to nonadherence because they perceive a social contract where the expectation is one of high adherence. The MARS takes steps to diminish this bias by sanctioning and normalising reports of nonadherence. However, this does not totally remove the effect self-presentational and recall biases that are inherent in all self-report measures.

For this reason, we will apply a combined approach to adherence assessment, where initial categorisation of patients into high vs. low on the basis of self-report is revised based on calcineurin inhibitor (CNI) blood monitoring (carried out in routine management for patients prescribed tacrolimus or ciclosporin). In this approach, reports of low adherence are accepted as self-presentational biases act in the opposite direction (reports of low adherence are more reliable than reports of high adherence). Patients who report high adherence are reclassified to low adherence on the basis of CNI results (e.g. if levels are undetectable then participant is assumed to be nonadherent)). Inferential statistics (independent t-test if data is approximately normally distributed, Mann-Whitney U test otherwise) will be used to determine if a difference exists between the adherence to immunosuppressive medication by Ab positive unblinded and blinded participants.

In order to explore the potential antecedents to participants’ adherence behaviours, they will also be asked to complete specially adapted versions of questionnaires relating to treatment intrusiveness (TIQ), symptoms associated with immunosuppressants (SAQ), beliefs about medicines (BMQ), and whether they are feeling anxious and/or depressed (HADS).  Inferential statistics (independent t-test if data is approximately normally distributed, Mann-Whitney U test otherwise) will be used to determine whether systematic differences exist between Ab positive unblinded and blinded participants. Differences between the blinded and unblinded HLA Ab screening groups will also be investigated.

Adherence and perceptual data will also be linked with clinical outcome data (graft failure, patient survival, graft dysfunction, acute rejection, culture positive infection, malignancy or diabetes) in order to determine any relationships. A regression model will be used for this analysis.

All patients taking part in the trial will be asked to complete the questionnaires at baseline and then at 24 months. Participants in whom the ‘clock is reset’ will also complete the questionnaire 24 months after this point in time. Questionnaires will be administered electronically.

# C) SCHEDULE OF ASSESSMENTS AND MEASURES

| Phase | Peri-Randomization | Post-Randomization | | | | | | | | | | | |  |  |
| --- | --- | --- | --- | --- | --- | --- | --- | --- | --- | --- | --- | --- | --- | --- | --- |
|  |  | Unblinded HLA Ab+ groups – Approximate times of assessment (+/- 1 week). Once stabilised, go to month 4 assessment | | | | | | All Groups – Approximate times of assessment  (+/- 3 months) | | | | | | |  |
| Study Week/month | Day -56 to 0 | Wk 2 | Wk 4 | Wk 6 | Wk 8 | Wk 10 | Wk 12 | Month  8 | Month 16 | Month  24 | | Month 32 | | Min 43 months |  |
| Informed consent | x |  |  |  |  |  |  |  |  |  | |  | |  |  |
| Inclusion/Exclusion Criteria | x^^[[27]](#footnote-27)^^ |  |  |  |  |  |  |  |  |  | |  | |  |  |
| Medical History inc. Drugs | x^^[[28]](#footnote-28)^^ |  |  |  |  |  |  |  |  |  | |  | |  |  |
| Transplant / sensitisation Hx | x |  |  |  |  |  |  |  |  |  | |  | |  |  |
| Registration / Demographics | x^^[[29]](#footnote-29)^^ |  |  |  |  |  |  |  |  |  | |  | |  |  |
| Weight / BP | x |  |  |  |  |  |  | x | x | x | | x | |  |  |
| Urine PCR | x |  |  |  |  |  |  | x | x | x | | x | |  |  |
| Haematology^[[30]](#footnote-30)^ | x |  | x |  | x |  | x | x | x | x | | x | |  |  |
| Biochemistry | x^[[31]](#footnote-31)^ |  | x^[[32]](#footnote-32)^ |  | x^8^ |  | x^8^ | x^[[33]](#footnote-33)^ | x^[[34]](#footnote-34)^ | x^9^ | | x^10^ | |  |  |
| [Calcineurin inhibitor] trough | x | x^^[[35]](#footnote-35)^^ | x | x | x | x | x | x | x | x | | x | |  |  |
| Total immunoglobulin  (or IgG, IgM +/- IgA) | x |  |  |  |  |  |  |  | x |  | x | |  | | |
| HLA antibody screening | x^^[[36]](#footnote-36)^^ |  |  |  |  |  |  | x^12^ | x^12^ | x^12^ | x^12^ | |  | | |
| Samples for scientific analysis | x^^[[37]](#footnote-37)^^ |  |  |  |  |  |  | x^13^ | x^13^ | x^13^ | x^13^ | |  | | |
| Apply optimized treatment protocol^[[38]](#footnote-38)^ |  | x | x | x | x | X | x |  |  |  |  | |  | | |
| See Trial-specific Nurse | x |  |  |  |  |  |  | x | x | x | x | |  | | |
| Record Medications | x |  |  |  |  |  |  | x | x | x | x | |  | | |
| Adverse Events Form |  | x | x | x | x | X | x | x | x | x | x | |  | | |
| Questionnaire for analysis of adherence / risk | x |  |  |  |  |  |  |  |  | x |  | |  | | |
| Questionnaire for health economics | x |  |  |  |  |  |  |  | x |  |  | |  | | |
| Primary Endpoint  (remote data collection) |  |  |  |  |  |  |  |  |  |  |  | | x | | |

# D) Reference list

(1) Moher D, Schulz KF, Altman DG. The CONSORT statement: revised recommendations for improving the quality of reports of parallel-group randomised trials. Lancet 2001 Apr 14;357(9263):1191-4.

(2) White IR, Thompson SG. Adjusting for partially missing baseline measurements in randomized trials. Stat Med 2005 Apr 15;24(7):993-1007.

# Amendments to version 1.0

Version 2.0:

Primary endpoint was changed from graft failure rates at 3 years to time to graft failure as per OUTSMART protocol V11 26_11_2015. Changes were made to this document throughout to reflect this; the sample size calculation was amended, and the primary analysis was changed to use a Cox proportional hazards model. Changes were also made to reflect the change as per the protocol of 8-monthly follow up visits instead of 4-monthly follow up visits.

Version 2.1:

Changes were made to the definition and analysis of the graft dysfunction secondary outcome. The exploratory analyses were clarified. Amendments were made to the Adherence and Risk assessment analyses. Minor changes to wording to bring in line with OUTSMART protocol.

Version 2.2:

Clarified in some sections the three separate groups being analysed (HLA +ve DSA, HLA +ve NDSA and overall). Added more information on descriptives by HLA Status for participants who became HLA +ve through re-screening. Other minor changes to wording as per DMC comments.

Version 2.3:

Amendments made to primary analysis following the COVID-19 pandemic; change to final data collection of the primary endpoint and addition of sensitivity analysis for later data collection.

Version 2.4:

Amendment to clarify change to analysis timing (pre and post data lock) following impact of COVID-19 pandemic on KHP-CTO monitoring of primary endpoint and subsequently database lock (see section 1.8.1).

## SOP relating to HLA antibody determination

| **CATEGORY**: | Tissue Typing Laboratory Work Instructions | **SOP** **Number**: 5 | **Version** 6.0 (30/09/2019) |
| --- | --- | --- | --- |
| **TITLE**: | **Detection of HLA Antibodies in Participant Samples for OuTSMART study** | | |

| **1.0** | **Title** |  |
| --- | --- | --- |
|  | **Tissue typing for OuTSMART Trial project** | |

| **2.0** | **Purpose** |  |
| --- | --- | --- |
|  | **To describe the procedure for detection of HLA antibodies in participant samples for the OuTSMART Trial** | |
|  | Serum is collected from whole blood by centrifugation and frozen. An aliquot is taken and analysed for the presence of HLA class I and II antibodies. | |

| **3.0** | **Definitions and Abbreviations** | |  |
| --- | --- | --- | --- |
|  | IgG/PE | Goat anti-human IgG Conjugated to phycoerythrin | |
|  | PBS | Phosphate Buffer Saline | |
|  | NC | Negative control | |
|  | PC | Positive control | |
|  | MFI | Mean fluorescence intensity | |

| **4.0** | **Equipment and Reagents** | | | |  |  |  |  |  |
| --- | --- | --- | --- | --- | --- | --- | --- | --- | --- |
|  | 4.1 | Equipment | | | | | | | |
|  |  | 4.1.1 | Vacuum manifold and pump | | | |  | For filter plate method | |
|  |  | 4.1.2 | Orbital Mixer | | | |  |  | |
|  |  | 4.1.3 | Luminex Analyser | | | |  |  | |
|  |  | 4.1.4 | Bench top microcentrifuge | | | |  |  | |
|  |  | 4.1.5 | Filter Plates | | | | Millipore Multiscreen filter plates  Cat no: MABVN1250 | For filter plate method | |
|  |  | 4.1.6 | Pre-cut transparent microplate sealers | | | | Greiner Bio-one  Cat no: 676001  Supplied by Jencons-PLS  Cat no: 488-097 |  | |
|  |  | 4.1.7 | Aluminium foil | | | |  |  | |
|  |  | 4.1.8 | Swinging bucket rotor for 96 well SSP tray (1300g/2600 rpm) | | | |  | For spin and flick method | |
|  |  | 4.1.9 | Microtube plate V bottom (G&N Laboratory: MA612V96) | | | |  | For spin and flick method | |
|  |  | 4.1.10 | 96 well low profile SSP tray | | | |  | For spin and flick method | |
|  | 4.2 | Reagents | | | | | | | |
|  |  | 4.2.1 | | Whole blood | | Subject Source | | |  |
|  |  | 4.2.2 | | PBS | | MP Biomedicals | | | LCC CAT no.: 2810305  (Dissolve one tablet in 100ml distilled water and store at 4°C. Once prepared, discard after 1 month.) |
|  |  | 4.2.3 | | Goat anti-human IgG Conjugated to phycoerythrin (freeze dried 100x concentrated and stored 4°C.) | | OneLambda  Cat no: 03LSAB2 | | | Reconstituted before use by adding sterile water at least two hours prior to first use. The volume of water to be added is clearly stated on the bottle. The date must be recorded on the side of the bottle, with an expiry date of 6 months post reconstitution date, unless the expiry date provided on the stock is earlier. Once reconstituted the IgG/PE must be stored at 4°C.  For use, dilute antihuman IgG/PE 1:100 with LABScreen wash buffer i.e. 1 part IgG/PE plus 99 parts wash buffer. |
|  |  | 4.2.4 | | FlowPRA Class I and II negative control | | VHBio Ltd | | | 03FLNC  (Stored at -80oC. Once defrosted stored at 4°C.) |
|  |  | 4.2.5 | | NIBSC – negative control for FXCM and anti-HLA serology | | 09/112 | | | Reconstituted with 1ml 0.1% sodium azide and stored at 4°C for up to 1 month |
|  |  | 4.2.6 | | LABScreen Mixed Class I & II Antibody Screening kit – (500μl) | | VHBio Ltd  Cat no: 03LSM12 | | | Kit must be stored at -80^o^C. Once defrosted, store kit at 4°C. The date the vial of beads was defrosted, plus the date received in the lab should be recorded on the side of the vial. |
|  |  | 4.2.7 | | LABScreen PRA SA Combi kit (Class I)  – (125μl) | | VHBio Ltd  Cat no: 03LS1A04 | | | Kit must be stored at -80^o^C. Once defrosted, store kit at 4°C. The date the vial of beads was defrosted, plus the date received in the lab should be recorded on the side of the vial. |
|  |  | 4.2.8 | | LABScreen PRA SA Class II kit  – (125μl) | | VHBio Ltd  Cat no: 03LS2A01 | | | Kit must be stored at -80^o^C. Once defrosted, store kit at 4°C. The date the vial of beads was defrosted, plus the date received in the lab should be recorded on the side of the vial. |
|  |  | 4.2.9 | | 10 x Concentrated wash buffer (26ml). | | Provided with screening kit. | | | This must be diluted 1:10 with distilled water prior to use. i.e. 1 part wash buffer plus 9 parts water. Once diluted label with the Lot number, expiry date and initials, then store at 4°C ready for use. |

| **5.0** | **Procedures**  **Biological waste should be disposed of according to the current regulations.** | | | | | |
| --- | --- | --- | --- | --- | --- | --- |
|  | Note | Safety:   1. Gloves and lab coat must be worn at all times. 2. All pipettes and tips that have been used to transfer blood products should be discarded into double bagged clinical waste bins. 3. Spillages of blood products should be wiped up using absorbent paper. The contaminated surface should be wiped with a solution of 1% Virkon using absorbent paper. Absorbent paper should be disposed of in a clinical waste bin. | | | | |
| **5.0** | **Standard procedure** | | | | | |
|  | 5.1 | Sample Checking and Processing | | | | |
|  |  | 5.1.1 | | Samples should arrive in suitably labelled specimen bags. All specimens must be handled over a spill tray. Any soiled paperwork must be discarded in an appropriate waste sack as clinical waste. In this instance, sample details should be manually transcribed onto a clean form, indicating that the original form had to be discarded. | | |
|  |  | 5.1.2 | | The details on the sample bottle / tube should be checked against those on the accompanying request form. Any discrepancies should be noted on the form and identified to a senior member of staff, who will decide on a course of action. If there are discrepancies, details taken from the bottle should be used for data entry. | | |
|  |  | 5.1.3 | | Centrifuge clotted blood samples for 5 minutes at 1000g. | | |
|  |  | 5.1.4 | | After centrifugation of the sample, up to 2ml serum should be transferred to an appropriately labelled serum tube. This transfer should be carried out in such a way to ensure that the serum is transferred to the correct tube. | | |
|  |  | 5.1.5 | | Freeze and store serum sample at -20^o^C until required for testing. | | |
|  |  | Notes | | 1. If a serum sample is badly haemolysed, and deemed unfit for use by a qualified member of staff, the sample may be discarded. 2. Samples should NOT be ‘inactivated’ in any way including by the addition of EDTA, DTT or heat inactivation. 3. Last research visit samples: Month 32 samples and the last clock reset samples will be stored at -20oC. These samples will be sent to Guy’s Hospital Tissue Typing Laboratory for analysis. | | |
|  | 5.2 | Procedure for filter plate method | | | | |
|  |  | 5.2.1 | | Remove kit from fridge ensuring the beads and PE remain in the DARK as they are extremely light sensitive. | | |
|  |  | 5.2.2 | | Note the Lot number of the kit to be used and ensure that the template has been loaded onto the Luminex software. | | |
|  |  | 5.2.3 | | Enter on worksheet the lot numbers and expiry dates of the LABScreen kit, IgG/PE, wash buffer, positive control and negative control. Where appropriate note the date the vial was received and defrosted. | | |
|  |  | 5.2.4 | | For screening with LABScreen Mixed kit – the NIBSC negative and the positive control sample should be included.  For screening with either the Class I and II single antigen kits – the FlowPRA negative control sample plus a positive control sample should be included. | | |
|  |  | 5.2.5 | | Take a new filter plate, or the current ‘in use’ filter plate if enough unused wells are available. Label each well of the plate numerically (corresponding to the serum number on the worksheet) for each serum sample to be screened including positive and negative controls. Labelling must be in the vertical, e.g. sample 1 at A1, sample 2 at B1, sample 3 at C1 etc. | | |
|  |  | 5.2.6 | | Using cut down transparent microplate sealers cover all wells that are not being used for this test. This keeps unused wells clean for future use and ensures a good vacuum when washing with the vacuum manifold. | | |
|  |  | 5.2.7 | | For each well to be used pre wet the filter by adding 250μl of sterile water. Leave for 5 minutes. | | |
|  |  | 5.2.8 | | After this time gently aspirate the contents of the wells using the vacuum manifold. | | |
|  |  |  | | 5.2.8.1 | | Ensure all tubes are correctly attached to the vacuum pump and the reservoir is empty. |
|  |  |  | | 5.2.8.2 | | Dampen the top of the manifold by briefly running under the tap, this ensures a good seal for the vacuum. |
|  |  |  | | 5.2.8.3 | | Place filter plate on top of the manifold and press down. |
|  |  |  | | 5.2.8.4 | | Turn on the vacuum pump until the contents of the wells have been drawn out of the bottom. |
|  |  |  | | 5.2.8.5 | | Do not apply excess vacuum as this can damage the filter, and when beads are present cause them to be lost or become trapped in the filter. |
|  |  |  | | 5.2.8.6 | | Decant contents of reservoir into a slop pot containing 1% Virkon before discarding. |
|  |  | 5.2.9 | | Prepare the beads by briefly centrifuging the vial at 600-800g to remove any beads or liquid from the cap or walls of the vial, then thoroughly mix by vortexing for 30 seconds or repeat pipetting to evenly resuspend beads. | | |
|  |  | 5.2.10 | | Transfer 3μl of beads to each of the assigned wells. Addition to the wells must be performed very carefully ensuring the filter is not pierced with the pipette tip. | | |
|  |  | 5.2.11 | | Add 12μl of each serum to the appropriate wells. Mix the well contents using repeat pipetting. Again ensure the filter is not pierced with the pipette tip. | | |
|  |  | 5.2.12 | | Cover the plate with the plastic lid provided and wrap in foil to protect from light. | | |
|  |  | 5.2.13 | | Incubate for 30 minutes at room temperature (20-24°C) on the orbital mixer, set at 200 rotations per minute. | | |
|  |  | 5.2.14 | | Dilute the IgG/PE conjugate. Calculate the amount of conjugate required to add 100μl to each well plus three wells extra (with each well requiring 1μl of conjugate diluted in 99μl of wash buffer). Mix conjugate by pipetting. Cap the tube and wrap completely in foil to protect from the light. Store at room temperature until use. | | |
|  |  | 5.2.15 | | After 30 minute incubation, remove the foil and plastic lid from the plate and add 250μl of wash buffer to each of the wells. | | |
|  |  | 5.2.16 | | Gently aspirate the contents of the wells using the vacuum manifold as described in 5.2.8. | | |
|  |  | 5.2.17 | | Add 250μl of wash buffer to each well, and aspirate as described in 5.2.8. | | |
|  |  | 5.2.18 | | Repeat step 5.2.17 a further two times to give a total of three washes. | | |
|  |  | 5.2.19 | | Add 100μl of diluted conjugate to each well and cover plate with plastic lid provided and then wrap in foil to protect from light. | | |
|  |  | 5.2.20 | | Incubate plate for 30 minutes at room temperature (C) on the orbital mixer set at 200 rotations per minute. | | |
|  |  | 5.2.21 | | Remove plastic lid and add 150μl of wash buffer. Mix by gently tapping the side of the plate. | | |
|  |  | 5.2.22 | | Repeat steps 5.2.16-5.2.18. | | |
|  |  | 5.2.23 | | Add 80μl of room temperature PBS and repeat pipette to mix the well contents. | | |
|  |  | 5.2.24 | | The beads are now ready to be analysed. This must be performed within 3 hours to ensure the least chance of obtaining false positive and false negative results. | | |
|  | 5.3 | Procedure for ‘Spin and Flick’ method | | | | |
|  |  | 5.3.1 | | | Follow steps 5.2.1 – 5.2.4 | |
|  |  | 5.3.2 | | | Prepare the beads by briefly centrifuging the vial at 600-800g to remove any beads or liquid from the cap or walls of the vial, then thoroughly mix by vortexing for 30 seconds or repeat pipetting to evenly re-suspend beads. | |
|  |  | 5.3.3 | | | Add 2 µl of LABScreen beads to each test well of a V bottom plate using a multichannel dispenser. | |
|  |  | 5.3.4 | | | Add 8 µl of each test serum into the corresponding well and mix. Wrap in foil and incubate for 30 minutes at room temperature (20-24°C) on the orbital mixer. | |
|  |  | 5.3.5 | | | Dilute the IgG/PE conjugate. Calculate the amount of conjugate required to add 100μl to each well plus three wells extra (with each well requiring 1μl of conjugate diluted in 99μl of wash buffer). Mix conjugate by pipetting. Cap the tube and wrap completely in foil to protect from the light. Store at room temperature until use. | |
|  |  | 5.3.6 | | | Following incubation add 230 µl of diluted (1X) wash buffer to each well of the plate. Cover with tray seal and vortex. Centrifuge at 1300g for 5 minutes. | |
|  |  | 5.3.7 | | | Remove wash buffer from wells of plate by flicking and then blotting on absorbent paper, ensuring the plate is not re-inverted between the two actions. | |
|  |  | 5.3.8 | | | Repeat steps 5.3.6 and 5.3.7 twice to give a total of 3 washes. | |
|  |  | 5.3.9 | | | Add 100 µl of previously diluted PE conjugate to each well. Cover with plate seal and vortex. Wrap in foil and incubate for 30 minutes at room temperature (20-24°C) on the orbital mixer. | |
|  |  | 5.3.10 | | | Centrifuge plate at 1300g for 5 minutes | |
|  |  | 5.3.11 | | | Add 150ul wash buffer, cover with seal and vortex. Centrifuge at 1300g for 5 minutes. Repeat wash steps 5.3.6 – 5.3.7 twice to give a total of 3 washes. | |
|  |  | 5.3.12 | | | Add 80 µl of wash buffer to each well and re-suspend beads by pipetting up and down. Then transfer the beads to their corresponding positions in a low profile 96 well PCR tray. The samples are ready for data acquisition. | |
|  | 5.4 | Collecting data using the Luminex Analyser | | | | |
|  |  | 5.4.1 | Set up and calibrate Luminex analyser following local procedure. | | | |
|  |  | 5.4.2 | Create Luminex input file following local procedure. | | | |
|  |  | 5.4.3 | Load the patient data and create a batch on the Luminex system following local procedure. | | | |
|  |  | 5.4.4 | Run plate following local procedure. | | | |
|  |  | 5.4.5 | Export raw data for analysis. | | | |
|  | 5.5 | Analysis of data | | | | |
|  |  | 5.5.1 | The original hand signed work sheet should be filed in the research folder. | | | |
|  |  | 5.5.2 | Transfer raw data for analysis into HLA Fusion software following local procedure. | | | |
|  |  | 5.5.3 | Analysis should be performed using HLA Fusion v2.0 according to local procedure – except for the Cut off values and points detailed below. | | | |
|  |  | 5.5.4 | For Class I single antigen analysis ensure that the W6-32 box is ticked. | | | |
|  |  | 5.5.5 | For LABScreen mixed screening beads the negative control values should be taken from the NIBSC negative control serum. | | | |
|  |  | 5.5.6 | For the Single Antigen screening beads the FlowPRA negative control values should be used. | | | |
|  |  | 5.5.7 | The control values should fit in the following criteria: | | | |
|  |  |  | 5.5.7.1 | | | The bead count should be greater than 50 for each bead group. |
|  |  |  | 5.5.7.2 | | | The NC should be greater than 30 and ideally below 500, but should ALWAYS be less than 1000. |
|  |  |  | 5.5.7.3 | | | The PC should be greater than 1000 and at least twice the NC value. |
|  |  |  | 5.5.7.4 | | | The PC/NC ratio should be greater than 2. |
|  |  |  | 5.5.7.5 | | | Any values falling outside these guidelines should be flagged up and discussed with HOS or appropriate before recording results or repeating. |
|  |  | 5.5.8 | Samples with an NC value of greater than 1000 should be re-tested following treatment with Absorbout beads, produced by OneLambda and provided by VH Bio, following the manufacturers guidelines. | | | |
|  |  | 5.5.9 | For LABScreen mixed analysis a sample should be deemed positive if any Class I bead has a ratio greater than 1.3 and any Class II bead has a ratio greater than 2.5, for the Lot 18 LABScreen mixed bead kit tested using the method described above.  For Lot 19 LABScreen mixed bead kits, a sample should be deemed positive if any Class I bead has a ratio greater than 4.0 and when any Class II bead has a ratio greater than 5.5.  For Lot 20 LABScreen mixed bead kits, a sample should be deemed positive if  any Class I bead has a ratio greater than 1.6 and when any Class II bead has a  ratio greater than 4.0.  For Lot 22 LABScreen mixed bead kits, a sample should be deemed positive if  any Class I bead has a ratio greater than 1.5 and when any Class II bead has a  ratio greater than 3.0. | | | |
|  |  | 5.5.10 | Samples tested using the LABScreen single antigen beads will be regarded as positive for the trial if the mean fluorescence intensity (MFI) of any bead is ≥2000. If any of the positive beads represent a mismatched donor HLA antigen, this will be assigned as DSA+. The number of DSA with an MFI ≥2000 will be recorded to define the Ab 'burden' of an individual patient. | | | |

1. In the transplantation literature, this problem is called ‘late’ allograft failure, in which ‘late’ refers to the lifespan of the transplanted organ. We have changed the term to shift emphasis onto the recipient. [↑](#footnote-ref-1)
2. For example, a patient recruited at the beginning of the study into groups C or D, found to have developed HLA Ab on the final screening round, will transfer into groups A or B and remain in intensive follow up for another 32 months. [↑](#footnote-ref-2)
3. Including virology and pregnancy testing where appropriate. [↑](#footnote-ref-3)
4. For registration, need to know whether already on tacrolimus and / or MMF/myfortic. [↑](#footnote-ref-4)
5. Do this prior to taking blood for HLA Ab screening [↑](#footnote-ref-5)
6. Hb, WCC, platelet count at all time periods [↑](#footnote-ref-6)
7. Creatinine, Na^+^, K^+^, bicarbonate, calcium, CRP, lipid profile, glucose, HbA1c. [↑](#footnote-ref-7)
8. Creatinine, Na^+^, K^+^, glucose, HbA1c [↑](#footnote-ref-8)
9. Creatinine, Na^+^, K^+^, bicarbonate, calcium, CRP, glucose, HbA1c [↑](#footnote-ref-9)
10. As enrolment biochemistry [↑](#footnote-ref-10)
11. In those patients having optimization of tacrolimus – continue until trough levels achieved [↑](#footnote-ref-11)
12. At enrollment, on everyone. Beyond enrollment, send sample from recruits in unblinded HLA Ab-negative group and ALL blinded patients. [↑](#footnote-ref-12)
13. Ideally participant will see a physician once a month whilst being optimized. Visit details are recorded in an Optimisation Log and not in the eCRF. [↑](#footnote-ref-13)
14. Here h_A1_(t), h_B1_(t), etc. represent the graft failure hazard rates in each of the groups. [↑](#footnote-ref-14)
15. NB these should be collected as end-points and not reported as adverse reactions [↑](#footnote-ref-15)
16. As for infections and infestations [↑](#footnote-ref-16)
17. Normal transplant clinic monitoring as per unit protocol [↑](#footnote-ref-17)
18. As for infections and infestations [↑](#footnote-ref-18)
19. Patients with known hypersensitivity to any of the IMPs are excluded from the trial [↑](#footnote-ref-19)
20. NB these should be collected as end-points and not reported as adverse reactions [↑](#footnote-ref-20)
21. As for infections and infestations [↑](#footnote-ref-21)
22. Patients with known hypersensitivity to any of the IMPs are excluded from the trial [↑](#footnote-ref-22)
23. DM is one of the end-points and serum glucoses should be recorded on CRF [↑](#footnote-ref-23)
24. As for infections and infestations [↑](#footnote-ref-24)
25. NB these should be collected as end-points and not reported as adverse reactions [↑](#footnote-ref-25)
26. For example, a patient recruited at the beginning of the study into groups C or D, found to have developed HLA Ab on the final screening round, will transfer into groups A or B and remain in intensive follow up for another 32 months. [↑](#footnote-ref-26)
27. Including virology and pregnancy testing where appropriate. [↑](#footnote-ref-27)
28. For registration, need to know whether already on tacrolimus and / or MMF/myfortic. [↑](#footnote-ref-28)
29. Do this prior to taking blood for HLA Ab screening [↑](#footnote-ref-29)
30. Hb, WCC, platelet count at all time periods [↑](#footnote-ref-30)
31. Creatinine, Na^+^, K^+^, bicarbonate, calcium, CRP, lipid profile, glucose, HbA1c. [↑](#footnote-ref-31)
32. Creatinine, Na^+^, K^+^, glucose, HbA1c [↑](#footnote-ref-32)
33. Creatinine, Na^+^, K^+^, bicarbonate, calcium, CRP, glucose, HbA1c [↑](#footnote-ref-33)
34. As enrolment biochemistry [↑](#footnote-ref-34)
35. In those patients having optimization of tacrolimus – continue until trough levels achieved [↑](#footnote-ref-35)
36. At enrollment, on everyone. Beyond enrollment, send sample from recruits in unblinded HLA Ab-negative group and ALL blinded patients. [↑](#footnote-ref-36)
37. At enrollment, on everyone. Beyond enrollment, only on those identified by a * ‘star’ on trial documents / labels. Collection of these samples will continue throughout the trial as long as there are resources available. [↑](#footnote-ref-37)
38. Ideally participant will see a physician once a month whilst being optimized. Visit details are recorded in an Optimisation Log and not in the eCRF. [↑](#footnote-ref-38)
